# Natural products targeting programmed cell death: a novel therapeutic strategy for intervertebral disc degeneration

**DOI:** 10.1097/JS9.0000000000003380

**Published:** 2025-10-07

**Authors:** Shudong Li, Tao Yang, Rongchen Lu, Shang Chen, Shu Jia, Lu Zhang, Xu Gao, Sheng Gao, Zikun Wang, Chunyang Meng

**Affiliations:** a The First Clinical Medical School, Shandong University of Traditional Chinese Medicine, Jinan, Shandong, China; b Department of Spine Surgery, Affiliated Hospital of Jining Medical University, Jining, Shandong, China; c Department of clinical medicine, Qingdao Medical College of Qingdao University. Qingdao, Shandong, China

**Keywords:** apoptosis, intervertebral disc degeneration, natural products, programmed cell death, pyroptosis

## Abstract

Intervertebral disc degeneration (IVDD) serves as a central pathological mechanism in the development of spinal degenerative disorders. Its progression is closely associated with the dysregulated activation of various programmed cell death (PCD) pathways, including apoptosis, pyroptosis, necroptosis, ferroptosis, and autophagy. Recent investigations have highlighted the potential of natural products to modulate these cell death processes through multitarget mechanisms, making them promising candidates for IVDD intervention. In this study, a comprehensive literature search was conducted using the PubMed database (up to 2025), applying a dual search strategy to systematically identify relevant publications. Following the screening of titles and abstracts and the removal of nonoriginal publications, a total of 194 eligible studies were retained, covering 134 distinct natural products. The analysis revealed that these products are capable of targeting multiple PCD pathways in intervertebral disc cells and exhibit considerable therapeutic potential. These findings offer a theoretical framework and methodological reference for the development of novel treatments for IVDD. Nevertheless, limitations remain regarding natural products, particularly in terms of bioavailability and safety. Future efforts should focus on optimizing screening platforms and drug delivery strategies to enhance therapeutic efficacy while minimizing potential toxicity.

**HIGHLIGHTS**

- This is the first systematic review focusing on the targeted regulation of intervertebral disc degeneration (IDD) by natural products, filling a critical gap in the literature.
- A total of 14 861 IDD-related articles were screened and reviewed, with 126 promising natural products ultimately included.
- A validated candidate library of natural compounds effective against IDD was established, providing a solid foundation for therapeutic development.
- The review provides in-depth analysis of the key mechanisms by which natural products act on IDD, including anti-inflammatory, antioxidant, anti-apoptotic, and extracellular matrix-regulating pathways.
- It offers a comprehensive “compound–mechanism” framework, serving as a valuable theoretical reference for future drug development and the formulation of novel therapeutic strategies for IDD.

## Introduction

Intervertebral disc degeneration (IVDD) is a common, chronic disorder marked by the gradual loss of structural integrity in spinal discs. This progressive deterioration is a key factor underlying numerous spinal degenerative conditions – including lumbar pain, disc herniation, and spinal canal stenosis – that compromise spinal function. As a widespread health concern, IVDD poses a considerable threat to public well-being^[1]^. Its onset and advancement are closely associated with multiple factors, notably senescence, biomechanical overload, and persistent inflammation. Among these, aging is recognized as the predominant etiological factor, with studies showing that nearly 90% of individuals over the age of 50 years exhibit varying degrees of disc degeneration^[2]^. According to the China Labor Disability Burden Report, spinal conditions, including IVDD, now represent the foremost cause of disability and significantly hinder workforce productivity^[3]^. In light of an increasingly aging population, the incidence of IVDD continues to rise sharply, placing a growing strain on healthcare systems and economic resources^[4]^. IVDD is a complex pathological process characterized by a decrease in the number of nucleus pulposus cells (NPCs), annulus fibrosus cells (AFCs), and cartilage endplate cells (CEPCs), along with dysfunction and abnormal interactions among these cells. The unique structure of intervertebral discs (IVDs) leads to an internal environment characterized by low pH (acidic), high osmotic pressure, hypoxic conditions, and poor nutrient availability, resulting in suboptimal outcomes for disc tissue repair and regeneration^[5]^. Steroids, nonsteroidal anti-inflammatory drugs, analgesics, and physical therapy are traditional treatments for IVDD. While these methods can alleviate symptoms in the short term, they fail to address the root cause of the condition. Surgical treatments for IVDD carry various risks, including spinal instability, recurrence, and vascular or nerve damage. Emerging technologies such as stem cell therapy and extracellular vesicle therapy remain prohibitively expensive and require further safety evaluation. Therefore, further elucidation of the molecular pathways that govern IVDD is essential, as is the pursuit of innovative and efficacious treatment strategies^[6]^.

Programmed cell death (PCD) is a strictly controlled, genetically preordained process by which cells actively trigger their own demise. This essential mechanism not only underpins normal embryonic development and maintains tissue homeostasis but also enables adaptive responses to both physiological and pathological challenges. PCD can be instigated by intrinsic developmental signals or extrinsic environmental stressors, which operate through the activation of specific cell surface receptors and subsequent intracellular signaling cascades. These signals induce a cascade of transcriptional reprogramming and post-translational alterations, ultimately culminating in the execution of cellular demise^[7]^. Unlike necrosis caused by external damage or pathological factors, PCD is an internally programmed activation process characterized by highly regulated and functional mechanisms, and different forms of PCD exhibit distinct molecular pathways and morphological features (Table 1). PCD influences IVD through multiple pathways such as apoptosis, autophagy, and necroptosis. Targeting specific regulatory molecules within PCD pathways can effectively modulate the degenerative process by altering the PCD progression in cells^[8]^. Over the past decade, natural products derived from fruits, vegetables, and medicinal plants have garnered attention in preventing and treating IVDD. These bioactive compounds exhibit low toxicity, high accessibility, long-term applicability, and patient compliance, making them promising candidates for IVDD management^[9]^. Therefore, elucidating the mechanisms by which natural products target and regulate PCD to prevent and treat IVDD holds significant importance. This review systematically summarizes common forms of PCD in IVDD, including apoptosis, pyroptosis, necroptosis, ferroptosis, and autophagy, exploring their relationships with IVDD. We also summarize the roles and mechanisms of natural products. This work has been reported in line with the TITAN criteria. No artificial intelligence tools were used in the research and manuscript writing process^[10]^.

**Table 1:** Mechanisms and morphological characteristics of common PCD

Type of PCDs	Apoptosis		Pyroptosis	Necroptosis	Ferroptosis	Autophagy
	Intrinsic apoptosis	Exogenous apoptosis				
Date	1972		2001	2005	2012	1963
Author	J. Kerr/A. Wyllie/A. Currie		B.Cookson	JY. Yuan	B.Stockwell	Christian de Duve
Main pathways	Mitochondrial pathway, endoplasmic reticulum stress pathway	Death receptor pathway	Caspase-4/5/11; Caspase-1	PIPK1/RIPK3/MLKLpathway	GPX4-dependent pathway, System Xc- pathway, Lipid metabolism pathway	Macroautophagy, Microautophagy, Chaperone-mediated autophagy
Cell volume	Shrinkage	Shrinkage	Swell	Swell	Shrinkage	Nonsignificant
Nuclear membrane integrity	No	No	Yes	No	No	Yes
Chromatin Margination	Yes	Yes	No	No	No	No
DNA fragmentation	Yes	Yes	Yes	No	No	No
Plasma Membrane Disruption	Blebbing	Blebbing	Yes	Yes	Yes	No
Mitochondria damage	Yes	Yes	Yes	Yes	Yes	Yes
Biochemical features	Slow formation of a limited number of apoptotic bodies	Rapid formation of a significant number of apoptotic bodies	Formation of pyroptotic bodies	Formation of the Necrosome Complex	Lipid Peroxidation	Formation of autophagosome and autolysosome

## Method

**Figure 1.**
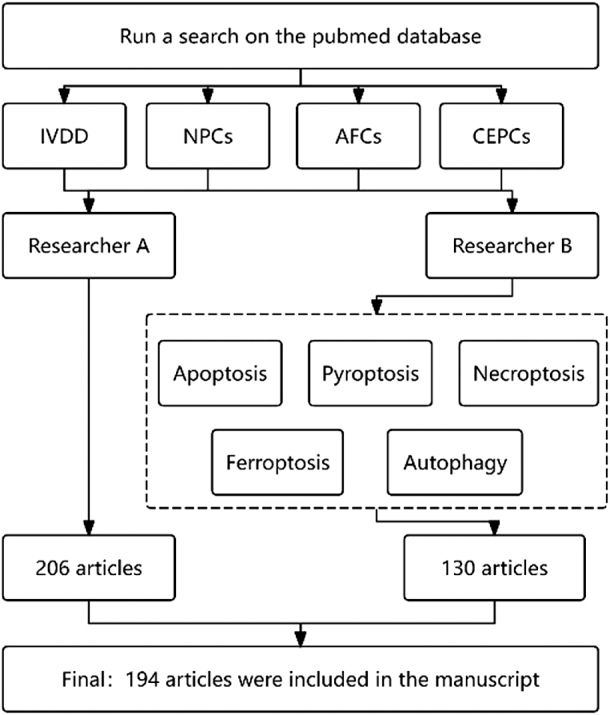
Research Protocol Design Framework. Based on the research design, Researcher A initially retrieved 14 861 articles, which were narrowed down to 206 articles after screening. Researcher B further refined the search by combining keywords with Researcher A’s strategy, yielding 1635 articles, of which 130 were retained after full-text review. Ultimately, 194 articles were included in the study. Despite overlapping search scopes, this dual-researcher approach aimed to minimize human bias and maximize the inclusion of relevant studies in the review.

We conducted a comprehensive search in the PubMed database for studies on IVDD published prior to 2025. To ensure maximum inclusion of relevant research, the study employed the following strategies (Fig. 1): (1) Researcher A conducted individual searches using the following keywords: IVD degeneration, NPCs, AFCs, and CEPCs; (2) Researcher B conducted a combined search using the following keywords in conjunction with those employed by Researcher A: apoptosis, autophagy, pyroptosis, necroptosis, and ferroptosis. (3) Articles on natural product therapies for IVDD were manually screened through the abstract and title, with case reports, letters, reviews, and meta-analyses excluded. (4) Merge the search results from both researchers and remove duplicate articles. The final version of this review is based on an analysis of 194 relevant sources.

## Natural products targeting apoptosis for IVDD

### Apoptosis

**Figure 2.**
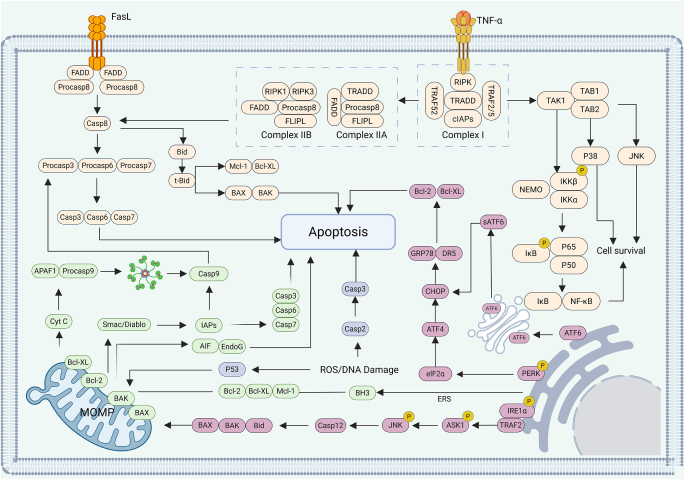
Molecular mechanisms of the major pathways of apoptosis created in BioRender (2025) https://BioRender.com/fdsusxu.

Apoptosis stands as the earliest characterized form of PCD. In 1972, Kerr described this phenomenon and coined the term “apoptosis,” initiating extensive research into PCD^[11]^. Consequently, apoptosis was initially used interchangeably with PCD. As one of the most common forms of PCD, apoptosis can be triggered by membrane receptors or internal stress signals. Upon receiving apoptotic signals, a cascade of cysteine-aspartic proteases (caspases) is activated, leading to cytoplasmic condensation, mitochondrial swelling, and cellular shrinkage. The nucleus undergoes pyknosis with chromatin margination. Nuclear DNA degrades into nucleosome-sized fragments, and the plasma membrane invaginates to encapsulate cellular debris, forming apoptotic bodies. These Apoptotic bodies formed during PCD are ultimately phagocytosed and degraded by neighboring immune cells, thereby completing the apoptotic process. Apoptosis is essential for numerous physiological events, including embryonic development, tissue remodeling, aging, and the preservation of cellular equilibrium^[12]^. The apoptotic cascade is primarily driven by the orderly activation of caspases. Initiator caspases (caspase-8/9) are activated through conformational changes or by assembling into multiprotein complexes in response to specific stimuli. These initiator caspases subsequently activate downstream effector caspases via proteolytic cleavage. Effector caspases then target a range of intracellular substrates. In addition, effector caspases may enhance the apoptotic signal by further activating other caspases, thereby accelerating cell death. The key signaling pathways regulating apoptosis include the mitochondrial (intrinsic) pathway, the endoplasmic reticulum stress (ERS)-mediated pathway, and the death receptor (extrinsic) pathway (Fig. 2).

The mitochondrial (intrinsic) pathway plays a pivotal role in PCD. Caspase-9 acts as the initiator caspase. Upon receiving apoptotic stimuli, mitochondria undergo MOMP, leading to the release of Cyt C into the cytosol. Cyt C binds to APAF-1, triggering its oligomerization and the exposure of its CARD. The APAF-1 recruits procaspase-9 and forms the apoptosome, which facilitates the activation of caspases. Within this complex, procaspase-9 undergoes autocleavage to become active caspase-9, which then triggers the activation of effector caspases such as caspase-3/7 to execute apoptosis^[13]^. MOMP is intricately regulated by the Bcl-2 protein family, which comprises three functional groups: (1) pro-apoptotic proteins (such as BAX and BAK) that induce MOMP; (2) anti-apoptotic proteins (such as Bcl-2 and Bcl-xL) that inhibit apoptosis by sequestering BAX and BAK; and (3) BH3-only proteins, which modulate apoptosis by regulating the activity of the other two groups^[14]^. Together, these proteins coordinate Cyt C release and apoptotic progression. In addition to Cyt C, MOMP also facilitates the release of other mitochondrial proteins such as Smac (also named Diablo). In vertebrate cells, XIAP suppresses apoptosis by binding to caspase-9. Smac antagonizes XIAP by competitively binding to it, thereby sustaining caspase activity^[15]^. OMI (HTRA2) exerts a similar inhibitory effect on XIAP. A variant of the mitochondrial apoptotic pathway involves MOMP without caspase activation, resulting in CICD. While factors like endonuclease G and AIF are believed to mediate CICD, their roles remain incompletely understood. Notably, although MOMP occurs in CICD, this process is distinct from classical apoptosis and exhibits significantly lower efficiency.

The ERS pathway constitutes a vital cellular mechanism for responding to disturbances such as protein misfolding and calcium dysregulation within the endoplasmic reticulum (ER). Although typically categorized under the intrinsic apoptotic pathway, ERS possesses distinct regulatory features. ER homeostasis results in an accumulation of misfolded proteins, which in turn triggers the activation of the UPR^[16]^. The UPR is orchestrated by three major ER membrane-resident sensors: IRE1α, PERK, and ATF6^[17]^. IRE1α detaches from the chaperone GRP78 (also known as BiP) under stress conditions. It subsequently oligomerizes, undergoes autophosphorylation, and activates its RNase domain. This activation triggers the noncanonical splicing of XBP1 mRNA, generating the transcriptionally active XBP1s. In turn, XBP1s upregulates the capacity of the endoplasmic reticulum to correctly fold proteins and facilitates the degradation of misfolded proteins. IRE1α also facilitates the selective decay of certain mRNAs and precursor microRNAs via a mechanism termed RIDD^[18]^. Under prolonged ER stress, IRE1α recruits TRAF2 and ASK1, activating the JNK and caspase-12 pathways, thereby initiating apoptosis^[19]^. Similarly, PERK dissociates from GRP78, dimerizes, and undergoes autophosphorylation. It then phosphorylates eIF2α, resulting in a global reduction in protein synthesis while paradoxically enhancing the selective translation of ATF4. ATF4 increases the transcription of CHOP – a transcription factor that favors apoptosis by upregulating pro-apoptotic mediators such as GRP78 and DR5 and downregulating antiapoptotic Bcl-2 proteins^[20,21]^. Unlike other ER stress sensors, ATF6 responds to endoplasmic reticulum stress by relocating to the Golgi apparatus, where site-specific proteolytic cleavage activates it, yielding the functional transcription factor ATF6p50. Once activated, ATF6p50 translocates into the nucleus and modulates the expression of target genes associated with protein folding homeostasis and programmed cell death, such as CHOP and MCL-1^[22]^. Collectively, these pathways illustrate the dual role of the ERS response: Initially, it functions to re-establish cellular homeostasis; however, prolonged or unresolved stress leads to a functional transition, whereby it begins to facilitate apoptotic pathways.

The death receptor (extrinsic) pathway is triggered by the binding of specific ligands to death receptors situated on the cell’s plasma membrane. These receptors, classified under the TNFR superfamily, are defined by the inclusion of a cytoplasmic DD that facilitates specific protein-protein interactions. Prominent ligand–receptor pairs include FasL and FasR, TNF-α and TNFR1, as well as TRAIL and DR4/5^[23]^. In the FasL/FasR signaling system, FasL binds to FasR in a trimeric form, exposing the DD and facilitating interaction with the FADD. FADD, through its DED, recruits procaspase-8, culminating in the assembly of the DISC. Within the DISC, procaspase-8 undergoes self-cleavage to become activated, initiating a cascade of downstream effector caspases that ultimately execute the apoptotic program^[24]^. The TNF-α/TNFR1 pathway operates similarly, where TNF-α binds to TNFR1, exposing the binding site of the DD and the TRAF2. TNFR1 then undergoes dimerization, associating with the TRADD, which stabilizes TRAF2 binding and recruits RIPK1, as well as the cIAP1 and cIAP2 to form complex I. This complex activates NF-κB, initially preventing apoptosis^[25]^. However, when NF-κB signaling is disrupted or its survival genes are inactive, a secondary signaling event occurs. This process involves the formation of either complex IIA, through the recruitment of FADD and procaspase-8, or complex IIB, involving RIPK1, FADD, and procaspase-8, both of which lead to apoptosis via caspase-8 activation^[26]^. The apoptotic mechanism initiated by TRAIL receptor ligation closely mirrors that of FasR. TRAIL receptor engagement recruits FADD to its DD, binding and dimerizing procaspase-8, which then activates apoptosis^[27]^. Interestingly, cells deficient in FADD or caspase-8 exhibit resistance to TRAIL-induced apoptosis. Furthermore, the TRAIL receptor DR5 is capable of independently initiating apoptotic signaling, regardless of TRAIL binding. Under ERS, DR5 expression is upregulated, and spontaneous oligomerization of DR5 within the ER can trigger caspase-8 activation, leading to apoptosis. Collectively, these pathways highlight the complexity and specificity of the death receptor-mediated apoptosis, emphasizing the essential roles of death receptors, adaptor proteins, and caspases in regulating this process.

### Apoptosis in IVDD

Apoptosis is a key contributor to the structural and functional degeneration of IVDs. The apoptotic pathways in IVD cells lead to cellular loss and ECM degradation, thereby accelerating disease progression^[28]^. Notably, apoptosis follows stage-specific regulatory patterns during IVDD. Experimental evidence shows that death receptor-mediated pathways and ERS pathways predominate in early-stage IVDD, while mitochondrial-dependent apoptosis becomes the primary driver in moderate-to-severe IVDD. This dynamic shift in apoptotic regulation during disease progression emphasizes the need for stage-specific therapeutic strategies, offering a molecular foundation for precision treatments tailored to distinct pathological stages^[29]^.

ERS-induced apoptosis serves as an early warning signal in the early stages of IVDD. The degeneration of NPCs marks the onset of IVDD and is primarily driven by ERS-mediated disruptions in protein homeostasis and inflammatory cascades. Several studies have reported significantly elevated levels of ERS-related proteins – such as GRP78 and CHOP – as well as apoptosis markers, including caspases, in the NP of IVDD patients. Furthermore, the expression levels of these markers show a positive correlation with the extent of disc degeneration, as classified by the Pfirrmann grading system^[30]^. In NPCs, ERS is mainly triggered by the accumulation of AGEs and proinflammatory cytokines such as IL-1β and TNF-α. AGEs activate GRP78, upregulate CHOP expression, and subsequently initiate caspase-3/12-dependent apoptotic pathways. This cascade reduces the synthesis of ECM components – such as type II collagen and proteoglycans – while enhancing catabolic activity^[30]^. Similarly, IL-1β and TNF-α exacerbate ERS-induced apoptosis in NPCs, further promoting IVDD progression^[31]^. *In vitro* studies have shown that excessive accumulation of ROS also upregulates GRP78, CHOP, and caspase-12, thereby activating ERS and inducing apoptosis in NPCs^[32]^. While hypoxia mitigates ERS in NPCs, ERS exacerbates the impairment of ECM secretion under hypoxic conditions^[33]^. Mechanical stress is another apoptosis-inducing factor. Wang *et al*^[34]^. demonstrated that inhibiting the mechanosensitive ion channel protein Piezo1 suppresses NPCs apoptosis. In AFCs, mechanical stress and oxidative stress synergistically activate ERS. Abnormal mechanical loads, such as compression and tension, significantly impact AFCs. Under static tension, the expression of ERS markers, such as CHOP and caspase-12, is significantly increased^[35]^. Additionally, cyclic tensile forces induce ERS through the ROS/NF-κB axis, thereby accelerating the process of apoptosis^[36]^. ERS is closely associated with calcium imbalance and metabolic abnormalities in CEPCs. Mechanical loading activates the Piezo1 ion channel, inducing calcium influx, which subsequently triggers the PERK/ATF4/CHOP signaling pathway, ultimately leading to cell apoptosis and ECM calcification^[34]^. The ERS pathway exhibits extensive crosstalk with both the mitochondrial and death receptor apoptotic pathways. Under oxidative stress conditions, excessive Ca^2^⁺ release from the ER can lead to enhanced Cyt C efflux from the mitochondrial matrix, thereby amplifying the activation of the intrinsic (mitochondrial) apoptotic pathway^[37]^. Furthermore, ERS can independently activate TRAIL receptors, triggering the caspase-8/FADD/RIPK1/NF-κB signaling cascade. This activation not only enhances the production of pro-inflammatory cytokines but also accelerates PCD^[38]^.

The death receptor-mediated apoptotic pathway is considered to be a major contributor to the progression of mild to moderate IVDD. Current studies on apoptosis in IVDD primarily concentrate on the FasL/FasR signaling axis^[39]^. Studies have demonstrated that FasL mRNA expression is comparatively low in normal lumbar IVD, while caspase-8 is nearly undetectable. However, in degenerative discs, the FasL/FasR pathway accelerates disease progression by promoting the apoptosis of NPCs and AFCs^[40,41]^. Furthermore, this pathway regulates CEPCs, as evidenced by significantly elevated FasL expression and increased apoptosis in CEPCs from degenerative IVD compared to nondegenerated discs. Notably, both FasL expression and the proportion of apoptotic CEPCs positively correlate with patient age^[42]^. In recent years, the FasL/FasR pathway in IVDD has become a subject of debate. Research indicates that FasL exhibits a dual “dose-effect” and “microenvironment-dependent” function in disc cell apoptosis in vitro: under specific conditions, it can either induce apoptosis to accelerate degeneration or exert protective effects by modulating alternative signaling pathways^[43]^. Importantly, the death receptor pathway is closely linked to inflammatory factor-induced microenvironmental deterioration. TNF-α and IL-1β can bind to FasR or TNFR, activating downstream caspases via caspase-8 and ultimately promoting apoptosis^[40]^. Additionally, IL-2 has been found to activate the death receptor pathway in a dose-dependent manner, inducing apoptosis and ECM degradation, thereby exacerbating IVDD progression^[44]^. These findings highlight the complexity of the death receptor pathway’s regulatory mechanisms in IVDD, suggesting that its specific effects are influenced by a dynamic interplay of microenvironmental factors and signaling pathways.

The mitochondrial pathway plays a central role in the moderate to advanced stages of IVDD, serving as a primary mechanism for apoptosis induced by oxidative stress and metabolic dysregulation. BAX facilitates apoptosis by triggering the opening of voltage-dependent anion channels in the mitochondria, which increases mitochondrial membrane permeability and leads to rupture. In contrast, Bcl-2 protects normal disc tissue from apoptosis by maintaining mitochondrial membrane stability. Studies have shown that in the degenerated NP tissue of IVDD patients, he ratio of Bcl-2 to BAX is markedly decreased, and the proportion of BAX-positive cells is positively correlated with the severity of IVDD^[45]^. Genetic polymorphism analysis further reveals that the −938C>A variant in the Bcl-2 gene may significantly increase the susceptibility of the Chinese Han population to IVDD by affecting its transcriptional activity^[46]^. The inflammatory microenvironment is a hallmark of IVDD. IL-1β and TNF-α exacerbate mitochondrial dysfunction through paracrine or autocrine mechanisms. Shen *et al*^[47]^ reported that IL-1β is significantly elevated in degenerated IVDs, where it facilitates Cyt C release by downregulating Bcl-2 and upregulating BAX. This subsequently triggers the caspase cascade, leading to apoptosis. In addition, oxidative stress is regarded as a primary “initiator” of IVDD. AOPPs, novel biomarkers of oxidative stress, are significantly elevated in IVDD patients. Inhibition of the MAPK pathway has been demonstrated to protect NPCs from oxidative damage and mitochondrial dysfunction induced by AOPP^[48]^. Moreover, the buildup of AGEs in NP tissue is positively associated with the progression of IVDD. AGEs promote mitochondrial ROS accumulation, upregulate BAX expression, and downregulate Bcl-2, thereby accelerating apoptosis^[49]^. It also plays a role in the apoptosis of AFCs in rabbits^[50]^. The exacerbation of oxidative stress leading to AFCs apoptosis is another critical pathological mechanism of IVDD. Studies have demonstrated that H_2_O_2_ markedly decreases the viability of AFCs in a dose- and time-dependent manner, inducing apoptosis through the mitochondrial pathway. Notably, TGF-β1 attenuates H_2_O_2_-induced autophagy and apoptosis by downregulating the ERK pathway in AFCs, suggesting its potential cytoprotective role^[51]^. Additionally, Excessive mechanical stress triggers apoptosis in AFCs by activating the mechanosensitive ion channel Piezo1, which subsequently engages the Calpain2/BAX/Caspase-3 signaling cascade, thereby contributing to the advancement of IVDD^[52]^.

### The regulation of apoptosis by natural products

In the field of innovative drug development, natural products have garnered significant attention due to their long-standing applications in ethnopharmacology and are widely adopted as crucial intervention strategies in maintenance therapy. Currently, numerous natural products have been screened as effective apoptotic regulators for treating IVDD. These bioactive molecules offer novel therapeutic targets for delaying IVDD progression by regulating apoptosis.This study examined 95 research articles on natural products that regulate apoptosis in IVD cells, identifying 76 distinct compounds. These were categorized into 31 flavonoids, 24 terpenoids, 7 phenolic acids, 7 alkaloids, and smaller groups of coumarins, lignans, among others. The analysis indicated that the primary focus is on targeting nucleus pulposus cell apoptosis to delay IVDD progression, accounting for 90.5% of the studies (86 articles). Other cell types investigated include annulus fibrosus cells (3.1%, 3 articles), cartilage endplate cells (4.2%, 4 articles), and nucleus pulposus-derived mesenchymal stem cells (2.1%, 2 articles). Four natural products – resveratrol, paeoniflorin, naringin, and baicalin – demonstrated regulatory effects on two or more cell types. This systematic categorization offers valuable insights into current research trends and highlights potential multitarget therapeutic candidates for IVDD intervention. The study demonstrated that resveratrol inhibits IVDD through multipathway regulation of apoptosis in NPCs, AFCs, and CEPCs^[53–55]^. Paeoniflorin mitigates NPC apoptosis through modulation of Bcl-2/BAX expression and suppression of the mitochondrial apoptotic initiator caspase-9^[56]^, while also inhibiting AFC apoptosis via the FasL/FasR signaling pathway^[41]^. Naringin inhibits apoptosis in NPMSCs via activation of the PI3K/Akt pathway, thereby mitigating intervertebral disc degeneration^[36]^. AFC apoptosis via mitochondrial pathway suppression^[57]^. Baicalin attenuates oxidative stress to inhibit apoptosis in both NPCs and CEPCs^[58]^. Additionally, two natural products specifically targeting CEPC apoptosis were identified: chlorogenic acid delays cartilage endplate degeneration and alleviates IVDD by suppressing the NF-κB signaling pathway^[59]^, while amygdalin inhibits CEPC apoptosis through synergistic effects with hydroxysafflor yellow A^[60]^.

Given the extensive number of natural products regulating NPC apoptosis and overlapping mechanisms, a subset of these compounds and their regulatory pathways is summarized in Table 2. Detailed information on all 76 natural products, including their molecular mechanisms, is provided with additional data available in the supplementary materials (Supplementary Digital Content Table 1, available at: http://links.lww.com/JS9/F228).

**Table 2:** Natural products targeting apoptosis for IVDD treatment (partial)

	Natural products	Chemical class	Sources	Structure	Medianlethal dose (Mice)	Safety	Target cell types	Apoptosis-related mode of action	Therapeutic dose	Key regulatory mechanisms	References
1	Ginsenoside Rg3	Terpenoids	Panax ginseng, Panax japonicus	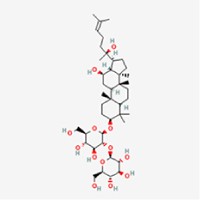	1250 mg/kg (Intraperitoneal)	Harmful if swallowed	NPC	BAX, Bcl-2,	60 μM (*In vitro*)	By inhibiting the phosphorylation level of AMPK, it ameliorates IL-1β-induced cell apoptosis.	^[61]^
									20 mg/kg/d (In vivo/Rat/Intraperitoneal injection)		
2	Ginsenoside Rg1	Terpenoids	Panax ginseng, Panax japonicus	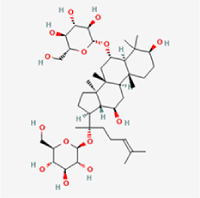	405 mg/kg (Intraperitoneal)	Harmful if swallowed	NPC	BAX, Bcl-2	100 μM (*In vitro*)	Inhibition of NF-κB signaling pathway activation suppresses IL-1β-induced NPC apoptosis.	^[62]^
									80 mg/kg/d (In vivo/Rat/Intraperitoneal injection)		
3	Kaempferol	Flavonoids	Hydrangea serrata, Caragana frutex	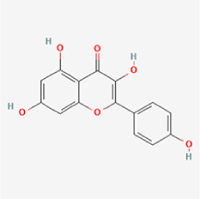	5 g/kg	IARC Carcinogenic Classes:Group 3:	NPC	BAX	10 μM (In vitro)	By inhibiting the phosphorylation level of AMPK, it ameliorates inflammatory factors-induced cell apoptosis	^[63]^
									50 mg/kg/d (*In vivo*/Rat/Intragastrically)		
4	Phillyrin	Phenolic acids	Bupleurum wenchuanense, Osmanthus fragrans	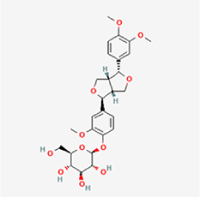	Unknown	Unknown	NPC	BAX, Bcl-2, Caspase-3	10 μM,20 μM (*In vitro*)	Inhibition of NF-κB pathway activation and suppression of ROS generation effectively alleviates IL-1β-induced extracellular matrix degradation and mitigates apoptotic responses.	^[64]^
5	Isoliquiritigenin	Flavonoids	Glycyrrhiza, pallidiflora、Morus cathayana	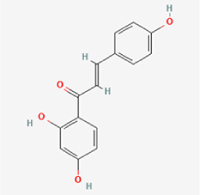	626 mg/kg (Speciesunspecified)	Causes skin and serious eye irritation, May cause respiratory irritation	NPC	BAX, Bcl-2, caspase-3, caspase-9,	5 μM (*In vitro*)	Through a PPARγ-dependent pathway, it restores ECM balance by reducing oxidative stress, improving mitochondrial function, and inhibiting apoptosis in nasopharyngeal carcinoma cells.	^[65]^
									40 mg/kg/d (*In vivo*/Rat/Intraperitoneal injection)		
6	Hyperoside	Flavonoids	Camellia sinensis, Geranium carolinianum	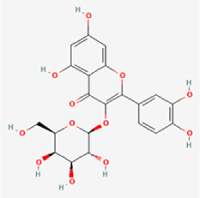	Unknown	Harmful if swallowed	NPC	BAX, Bcl-2, GPR78, PERK, CHOP, caspase-12	50 μM (*In vitro*)	SIRT1/NF-κB and Nrf2 pathways mitigate TNF-α-induced inflammation, ECM degradation, and ER stress-related apoptosis.	^[66]^
7	Taurine	Amino Acids	marine fish, shellfish	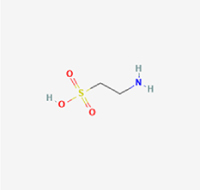	7 g/kg (Oral)	Causes skin and serious eye irritation. May cause respiratory irritation	NPC	GRP78, CHOP, caspase-12	40 μM (*In vitro*)	Suppression of ERS orchestrates cellular proteostasis in NPCs, effectively attenuating apoptotic cascades and ECM catabolic processes.	^[67]^
					6630 mg/kg (Intraperitoneal)						
					6 g/kg (Subcutaneous)						
8	Sulforaphane	Isothiocyanates	Broccoli, cabbage	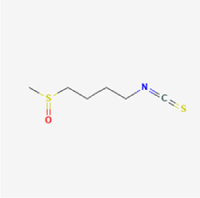	Unknown	Harmful if swallowed, Causes skin and serious eye irritation. May cause respiratory irritation	NPC	GRP78, CHOP, PERK, Eif2α, caspase-3, caspase-12	10 μM (*In vitro*)	Activation of the Nrf2/HO-1 signaling axis ameliorates ERS and suppresses apoptosis by scavenging intracellular ROS accumulation.	^[68]^
									10 μM/2 μL/qow (*In vivo*/Rat/Intradiscal injection)		
9	Fucoxanthin	Terpenoids	Jania, Corbicula sandai	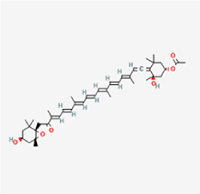	>10 g/kg	Causes serious eye irritation.	NPC	PERK, eIF2α, ATF4, CHOP	30 μM (*In vitro*)	By upregulating SIRT1, it inhibits the activation of the PERK-eIF2α-ATF4-CHOP pathway, reduces ECM degradation, and suppresses apoptosis induced by ERS.	^[69]^
									10 mg/kg/w (*In vivo*/Rat/Intraperitoneal injection)		
10	Berberine	Alkaloids	goldenseal, barberry, Oregon grape	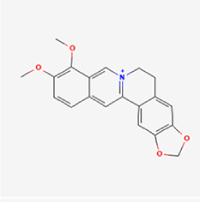	329 mg/kg (Oral)	Decreased fetal weight (Maybe)	NPC	Bcl-2, caspase-3, caspase-9, GRP78, caspase-12, CHOP	8 μM (*In vitro*)	Modulation of ERS and autophagy prevents oxidative stress-induced apoptosis.	^[32]^
					18 mg/kg (Intraperitoneal)				150 mg/kg/d (*In vivo*/Rat/Intraperitoneal injection)		
11	Orientin	Flavonoids	Cecropia hololeuca, Gentiana algid	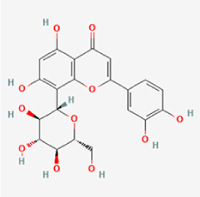	>2 g/kg	Unknown	NPC	BAX, Bcl-2, caspase-3, GRP78, CHOP	40 μM (*In vitro*)	Upregulation of AMPK/SIRT1 restores ECM and ER homeostasis, thereby attenuating oxidative stress.	^[70]^
									(In vivo/Rat/Intragastrically)		
12	Paeoniflorin	Terpenoids	Paeonia emodi, Paeonia obovata	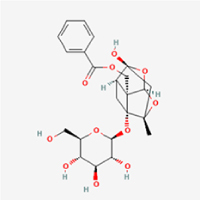	3530 mg/kg (Intraperitoneal)	Harmful if swallowed, somnolence (General depressed activity)	AFC	Fas, caspase-3,	8 μM (*In vitro*)	Inhibition of the Fasl—FasR signaling pathway activation reduces fasl-induced apoptosis in annulus fibrosus cells.	^[41]^
					9530 mg/kg (Intravenous)						
13	Naringin	Flavonoids	Salvia officinalis, Citrus reticulata	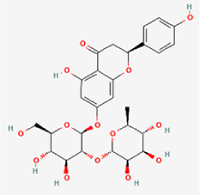	2 g/kg (Rat:Intraperitoneal)	Causes skin and serious eye irritation. May cause respiratory irritation	AFC	Cyt C,	100 μg/ml (*In vitro*)	Suppression of NF-κB signaling activation attenuates oxidative stress and restores mitochondrial homeostasis, thereby counteracting cyclic tensile loading-induced apoptosis in AFCs via coordinated modulation of redox imbalance and bioenergetic recovery.	^[36]^
									200 mg/kg/d (*In vivo*/Rat/Intragastrically)		
14	Resveratrol	Stilbenes	Polygonum cuspidatum	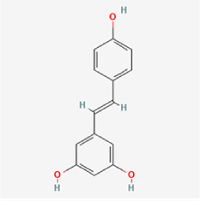	2 g/kg (Rat:Oral)	Causes skin and serious eye irritation. May cause respiratory irritation	AFC	BAX, Bcl-2, caspase-3	100 μM (*In vitro*)	Targeted attenuation of oxidative stress response in vitro counteracts TNF-α-mediated apoptotic signaling in AFCs.	^[55]^
15	Chlorogenic Acid	Phenolic Acids	Camellia sinensis, Meum athamanticum	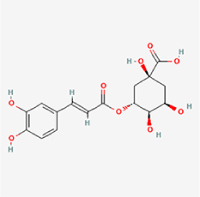	4 g/kg(Rat:Intraperitoneal:LDLo)	Causes skin and serious eye irritation. May cause respiratory irritation	CEPC	/	100 μM (*In vitro*)	Suppression of NF-κB signaling axis in the cartilaginous endplate milieu confers multifaceted cytoprotection by counteracting endplate chondrocyte apoptosis and attenuating dedifferentiation-associated phenotypic regression.	^[59]^
									100 mg/kg/d (*In vivo/*Mice/Intragastrically)		
16	Resveratrol	Stilbenes	Polygonum cuspidatum	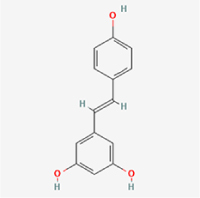	2 g/kg (Rat:Oral)	Causes skin and serious eye irritation. May cause respiratory irritation	CEPC	BAX, Bcl-2	30 μM (*In vitro*)	Modulation of TNF-α release and augmentation of IL-10 production significantly attenuate apoptosis in CEPCs through coordinated immunoregulatory mechanisms.	^[53]^
17	Amygdalin	Glycosides	Malus, Prunus salicina	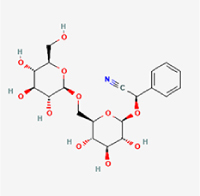	443 mg/kg (Oral)	Harmful if swallowed, Dyspnea, nausea or vomiting	CEPC	/	10 μM (*In vitro*)	Combined with hydroxysafflor yellow inhibits IL-1β-induced apoptosis in NPCs	^[60]^
18	Baicalin	Flavonoids	Scutellaria prostrata, Scutellaria scandens	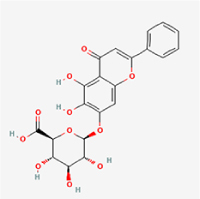	3081 mg/kg (Oral,Baicalein)	Causes skin and serious eye irritation. May cause respiratory irritation	CEPC	BAX, caspase-3	100 μM (*In vitro*)	Reduction of MDA levels and elevation of SOD/NO levels inhibits H2O2-induced oxidative stress in endplate chondrocytes and reduces apoptosis.	^[71]^

Considering the predominance of distinct apoptotic pathways at different stages of IVDD, we systematically reviewed the mechanisms of relevant bioactive compounds. This preliminary stage-specific classification seeks to facilitate precise modulation of IVDD progression and enhance therapeutic efficacy. Emerging evidence suggests that compounds from various chemical classes preferentially, though not exclusively, target specific apoptotic pathways. Phenolic acids, rich in hydroxyl groups, readily participate in intracellular redox reactions, triggering the UPR and thereby inducing ERS-mediated apoptosis, which may offer therapeutic benefits in early-stage IVDD. Certain terpenoids, including triterpenoid saponins, can modulate inflammation-associated apoptosis by inhibiting death receptor pathways such as Fas/FasL, highlighting their potential in early interventions. Flavonoids, widely investigated among natural products, combine strong antioxidant and anti-inflammatory effects, protecting cells from oxidative stress–induced mitochondrial apoptosis, and thus appear promising for moderate to advanced disc degeneration. Despite these insights, research on natural compounds that act via ERS and death receptor pathways in IVDD remains limited, underscoring the need for further validation and refinement of this classification through additional studies.

## Natural products targeting pyroptosis for IVDD

### Pyroptosis

Pyroptosis, identified as the first form of PCD following apoptosis, has reshaped the traditional binary classification of cell death, highlighting the diversity of PCD mechanisms. In 1986, Friedlander reported that exposing primary murine macrophages to anthrax lethal toxin triggered swift cytotoxicity, characterized by the discharge of intracellular components^[72]^. Six years later, in 1992, Zychlinsky observed a comparable response in macrophages infected with *Shigella flexneri*, a Gram-negative bacterium, although this process was initially misinterpreted as apoptosis^[73]^. By 2000, Brennan *et al*^[74]^ found that Salmonella infection led to macrophage death through a caspase-1-dependent inflammatory necrosis mechanism, characterized by membrane rupture and cytokine release – distinct features from apoptosis. In 2001, Cookson and Brennan introduced the term “pyroptosis” to define this caspase-1-mediated inflammatory cell death^[75]^. Early misclassification of pyroptosis as apoptosis was due to observable cellular swelling and membrane blebbing^[76]^. However, pyroptosis exhibits unique morphological characteristics. Upon pyroptotic signaling, inflammasomes assemble within cells^[77]^, leading to the oligomerization of ASC, which forms large protein complexes known as “pyroptotic bodies.” This mechanism leads to the creation of small pores in the plasma membrane, which in turn causes the leakage of cytoplasmic contents^[78]^. Additionally, mitochondrial swelling occurs, cristae structures disintegrate, and the UPR in ER is activated. These events culminate in rapid cell swelling and extensive membrane blebbing. When the density of membrane pores exceeds a critical threshold, the plasma membrane ruptures, causing cell disintegration and the release intracellular contents such as inflammatory mediators, thereby completing the pyroptotic process. In contrast to apoptosis, pyroptotic cells display minimal DNA fragmentation and maintain nuclear integrity; however, their plasma membrane integrity is significantly disrupted^[79]^.

**Figure 3.**
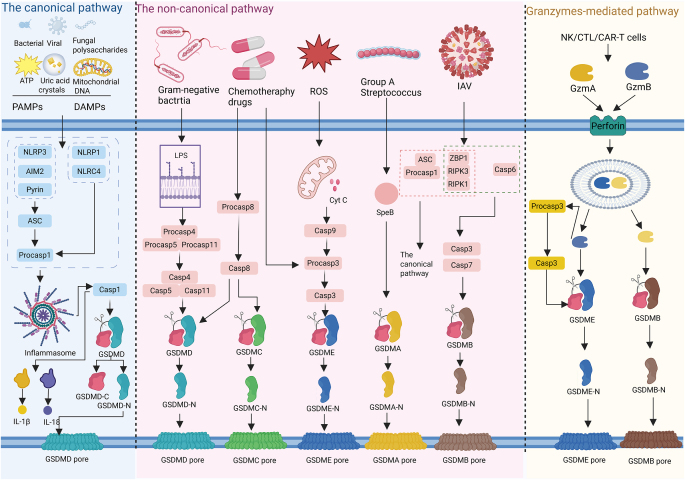
Molecular mechanisms of the major pathways of pyroptosis created in BioRender (2025) https://BioRender.com/01o4tqc.

The core regulatory mechanism of pyroptosis involves caspase family proteins, including caspase-1/11 in mice, and caspase-1/4/5 in humans, which activate pro-inflammatory factors such as IL-1β to initiate inflammatory responses^[80,81]^. The GSDM family proteins are central to this process, with GSDMD being the most extensively studied and functionally critical member. Upon pathogen infection or exposure to danger signals, GSDMD is cleaved, releasing the NT fragment, which oligomerizes to form pores in the plasma membrane^[78,82]^. The formation of pores disrupts osmotic equilibrium, leads to cell lysis, and promotes the release of pro-inflammatory mediators, thereby intensifying the inflammatory response^[83]^. Based on GSDMD cleavage mechanisms, pyroptosis is categorized into canonical and noncanonical pathways (Fig. 3).

The canonical pathway is inflammasome-mediated. The danger signals that induce pyroptosis primarily fall into two categories: PAMPs and DAMPs. PAMPs include bacterial components, viral nucleic acids, and fungal polysaccharides, while DAMPs consist of endogenous molecules such as ATP, uric acid crystals, and mitochondrial DNA^[84]^. Pyroptosis is initiated when PRRs, particularly NLRs, detect these signals. NLRs have a domain, known as NACHT, flanked by C-terminal LRRs and N-terminal CARD or PYD. Based on the composition of these domains, NLRs are classified into subtypes such as NLRP1, NLRP3, and NLRC4^[85]^. The ASC is a critical inflammasome component, possessing both PYD and CARD domains. NLRs recruit ASC to form an ASC-ASC helical fibril network, which subsequently recruits pro-caspase-1 via ASC’s CARD domain, assembling the inflammasome. Some PRRs, like NLRC4, which contain CARD domains, can directly recruit pro-caspase-1^[86]^. Following inflammasome assembly, caspase-1 is activated, cleaved into two subunits, and subsequently dimerizes to form the active mature enzyme^[87]^. Caspase-1 subsequently cleaves GSDMD, generating the N-GSDMD and C-GSDMD fragments. The N-GSDMD fragment then translocates to the plasma membrane, where it forms nonselective pores with an inner diameter of approximately 10–14 nm, resulting in cell swelling and pyroptosis^[76,88]^. Simultaneously, caspase-1 processes pro-IL-1β and pro-IL-18 into their active forms, which are subsequently released through the pores formed by GSDMD, thereby further amplifying the inflammatory response^[89]^.

The noncanonical pathway primarily distinguishes itself from the canonical pathway by its independence from PRRs. Caspase-4/5 in humans, and caspase-11 in mice, directly bind to intracellular LPS via their NT CARD, leading to their activation^[89]^. In contrast to caspase-1, these caspases do not directly process IL-1β and IL-18. Instead, these caspases cleave GSDMD, leading to potassium ion (K^+^) efflux, which in turn triggers the assembly of the NLRP3 inflammasome and facilitates the maturation and release of IL-1β and IL-18 via the NLRP3/caspase-1 pathway^[90,91]^. Advancements in pyroptosis research have revealed that certain chemotherapeutic agents can activate apoptosis-related caspases, such as caspase-3 and caspase-8, to cleave GSDME, producing its N-terminal fragment and inducing pyroptosis^[92,93]^. Recent studies indicate that chimeric antigen receptor T cells release granzyme B (GzmB), which can activate caspases to cleave GSDME, thereby triggering pyroptosis^[94]^. Furthermore, GzmB has been shown to directly cleave GSDME, leading to pyroptosis^[95]^. These findings challenge the traditional view that pyroptosis is exclusively activated by caspases, suggesting alternative pathways for its induction. However, further experimental validation is necessary to fully elucidate these mechanisms.

### Pyroptosis in IVDD

Pyroptosis, a pro-inflammatory type of PCD that has attracted significant attention in recent years, plays a pivotal role in the initiation and progression of IVDD. NPCs are the main targets of pyroptosis in IVDD, with their pyroptotic activation resulting in the accumulation of inflammatory mediators and the degradation of ECM within the IVDs^[96]^. Clinical analyses have shown markedly increased expression levels of pyroptosis-related markers, such as NLRP3, caspase-1, and IL-1β, in degenerated NP tissues, with these levels positively correlating with the severity of disc degeneration^[97]^. Inhibiting NPC pyroptosis effectively reduces the production and accumulation of pro-inflammatory cytokines, prevents ECM degradation, and decelerates IVDD progression^[98]^. The inflammasome has become a major focus of research^[99]^. Studies have demonstrated that suppressing NLRP1 or NLRP3 expression significantly downregulates the expression of cell death-associated genes and proteins, including ASC and caspase-1, thereby inhibiting NPC pyroptosis^[100]^. The complex regulatory mechanisms governing inflammasome activation offer numerous potential strategies for targeting inflammasome synthesis to suppress NPC pyroptosis and delay IVDD. Research suggests that enhancing PINK1/Parkin-mediated mitophagy counteracts LPS-induced mitophagy inhibition and mitochondrial dysfunction, effectively suppressing NLRP3 inflammasome activation^[101]^. Additionally, MFG-E8 exhibits therapeutic potential by mitigating H_2_O_2_-induced oxidative stress, mitochondrial dysfunction, and NLRP3 inflammasome activation through the Nrf2/TXNIP/NLRP3 axis, ultimately inhibiting NPCs pyroptosis and ECM degradation while alleviating IVDD progression^[102]^. Notably, verapamil has demonstrated therapeutic efficacy via this mechanism, emerging as a promising pharmacological candidate for IVDD treatment^[103]^. Furthermore, the cGAS-STING-NLRP3 axis and acid-sensing ion channels have been identified as effective targets for suppressing NLRP3 inflammasome activation and pyroptosis^[104,105]^. Despite these advancements, the role of the noncanonical pyroptosis pathway in NPCs death remains poorly understood in IVDD research. Although elevated caspase-5 expression has been observed in degenerative discs^[106]^, its precise function requires further investigation. Future studies focusing on the regulation of noncanonical pyroptosis pathways may yield novel therapeutic strategies for mitigating the pathological progression of IVDD.

Compared to NPCs, CEPCs and AFCs have received relatively less attention in current research. Studies on pyroptosis in CEPCs and AFCs remain limited, with most investigations confined to correlative observations, while their underlying molecular mechanisms remain largely unexplored. Fu *et al*^[107]^ established a mouse model of IVDD induced by lumbar spine instability surgery to examine the relationship between pyroptosis pathway activation and AFCs/CEPCs. Their results showed a notable upregulation of pyroptosis-associated factors, including NLRP3, caspase-1, and IL-1β, in these cell populations. Notably, these pathological changes were also validated in human degenerated IVD samples. Immunohistochemical analysis demonstrated a marked increase in Interleukin – 1α (IL-1α)- and IL-1β-positive cells within AFCs of degenerated discs, with expression levels correlating with IVDD severity^[108]^. Tang *et al*^[109]^ further verified the elevated expression of NLRP3, caspase-1, and IL-1β in human degenerated cartilage endplate tissues, reinforcing the involvement of pyroptosis in IVDD pathology.

### The regulation of pyroptosis by natural products

This study examined 9 natural products with the potential to inhibit IVDD by regulating pyroptosis. These compounds included 4 terpenoids (paeoniflorin, notoginsenoside R1, gamma-oryzanol, and aucubin), 2 alkaloids (Sinapine thiocyanate and magnoflorine), 1 flavonoid (morin), 1 quinone (rhein), and 1 pyranone (maltol). Although the specific molecular targets and mechanisms varied across studies, a common outcome was observed: inhibition of NLRP3 inflammasome activation via the canonical pyroptosis signaling cascade effectively suppressed pyroptotic activity in intervertebral disc (IVD) cells and attenuated disc degeneration. Notably, the NF-κB/NLRP3 pathway emerged as the most frequently explored regulatory axis in these studies (Table 3).

**Table 3:** Natural products targeting pyroptosis for IVDD treatment

	Natural products	Chemical class	Sources	Structure	Median lethal dose (Mice)	Safety	Target cell types	Pyroptosis -related mode of action	Therapeutic dose	Key regulatory mechanisms	References
1	Sinapine thiocyanate	Alkaloids	Brassica oleracea, Raphanus sativus	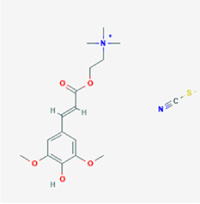	Unknown	Unknown	NPC	NLRP3, caspse-1	200 μM (*In vitro*)	Pathways involving NLRP3-mediated pyroptosis inhibition and JAK1/STAT3 signaling suppression were excluded from this analysis.	^[116]^
									8 mg/kg/d (*In vivo*/Rat/Intraperitoneal injection)		
2	Maltol	Pyranones	Bolbostemma paniculatum, Streptomyces	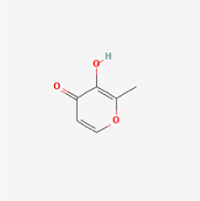	820 mg/kg (Subcutaneous)	Harmful if swallowed, Causes skin, eye and respiratory irritation	NPC	NLRP3, caspse-1, IL-1β	80 μM (*In vitro*)	Suppression of ECM degradation and inflammatory responses can be achieved through inhibition of the PI3K/AKT/NF-κB signaling cascade and NLRP3 inflammasome-driven pyroptosis.	^[118]^
									30 mg/kg/biw (*In vivo*/Mice/Intragastrically)		
3	Paeoniflorin	Terpenoids	Paeonia emodi, Paeonia obovata	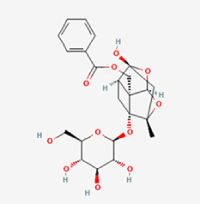	3530 mg/kg (Intraperitoneal)	Harmful if swallowed	NPC	NLRP3, caspase-1, GSDMD-N, ASC	400 μM (*In vitro*)	Alleviates extracellular matrix degradation and NLRP3 inflammasome-mediated pyroptosis in nucleus pulposus cells under acidotic conditions.	^[56]^
									20 mg/kg/d (*In vivo*/Rat/Intraperitoneal injection)		
					9530 mg/kg (Intravenous)						
4	Morin	Flavonoids	Maclura pomifera, Petasites formosanus	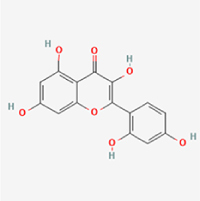	555 mg/kg (Intraperitoneal)	Toxic if swallowed, Toxic to aquatic life with long lasting effects	NPC	NLRP3, caspse-1, IL-1β	50 nM (In vitro)	Modulation of the TXNIP/NLRP3/Caspase-1 pathway contributes to the attenuation of pyroptosis in NPCs and exerts protective effects against IVDD.	^[119]^
									30 mg/kg/3d (*In vivo*/Mice/Intraperitoneal injection)		
5	Notoginsenoside R1	Terpenoids	Panax japonicus, Panax notoginseng	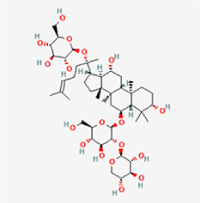	447 mg/kg (Intraperitoneal)	Harmful if swallowed	NPC	NLRP3, caspse-1, IL-1β, GSDMD-N,	50 μM (*In vitro*)	Promotes ECM release and reduces pro-inflammatory cytokine mRNA expression; inactivation of the NF-κB/NLRP3 pathway ameliorates cellular functional status and suppresses pyroptosis.	^[112]^
									20 mg/kg/w (*In vivo*/Rat/Intraperitoneal injection)		
					396 mg/kg (Intravenous)						
					1667 mg/kg (Subcutaneous)						
6	Magnoflorine	Alkaloids	Stephania tetrandra, Aquilegia eximia	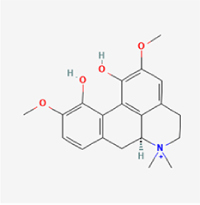	19 600 μg/kg (Intraperitoneal)	Harmful if swallowed, in contact with skin or if inhaled	NPC	NLRP3, ASC, caspase-1	300 μM (*In vitro*)	Concurrent suppression of HMGB1-MyD88-NF-κB signaling and NLRP3 inflammasome activation.	^[117]^
					20 mg/kg (Intravenous)						
					138 mg/kg (Subcutaneous)						
7	Rhein	Quinones	Rheum palmatum, Cassia tora, Polygonum multiflorum	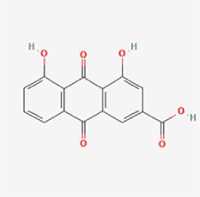	>5 g/kg (Oral)	Causes skin and serious eye irritation, May cause respiratory irritation.	NPC	IL-1β, NLRP3, caspase-1, GSDMD	25 μM (*In vitro*)	Amelioration of the LPS-induced inflammatory microenvironment modulates ECM metabolic imbalance and NLRP3 inflammasome assembly in NPCs, thereby suppressing pyroptosis.	^[120]^
					>25 mg/kg (Intravenous)						
8	Gamma-oryzanol	Terpenoids	Oryza sativa, Ophiocordyceps sinensis	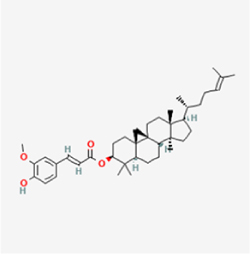	>25 g/kg (Oral,Rat)	Fatal in contact with skin	NPC	NLRP3, IL-1β, caspase-1,ASC	40 μM (*In vitro*)	Disruption of NF-κB signaling and suppression of ROS overproduction synergistically attenuate IL-1β-mediated NLRP3 inflammasome activation in NPCs.	^[113]^
									20 mg/kg/d (*In vivo*/Rat/Intragastrically)		
					100 mg/kg (Skin,Rat)						
					382 mg/kg (Intravenous)						
9	Aucubin	Terpenoids	Veronica kellereri, Plantago uniflora	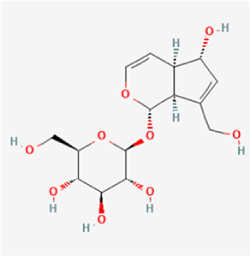	Unknown	Harmful if swallowed	CEPC	NLRP3, caspse-1	200 μM (*In vitro*)	Inhibition of NF-κB-NLRP3 inflammasome activation in chondrocytes ameliorates cartilaginous endplate degeneration in IVDD.	^[115]^
									10 mg/kg/d (*In vivo*/Mice/Intraperitoneal injection)		

Paeoniflorin, a monoterpene glycoside extracted from the root of Paeonia lactiflora (Ranunculaceae), has exhibited notable antidepressant effects through the suppression of pyroptosis. It also alleviates neuropathic pain by inhibiting spinal cord NLRP3 inflammasome activation^[110,111]^. Research by Dai *et al*^[56]^ suggests that a sustained abnormal elevation of intracellular calcium levels can excessively activate the NLRP3 inflammasome, thereby promoting pyroptosis. Paeoniflorin significantly reduces calcium ion levels in NPCs, inhibiting NLRP3 inflammasome activation. Calcium-regulating channels in acidic environments may serve as novel therapeutic targets. Notoginsenoside R1 has been reported to attenuate inflammatory signaling and suppress pyroptosis in NPCs via inhibition of the NF-κB/NLRP3 axis^[112]^. Gamma-Oryzanol, primarily found in vegetable oils such as rice bran and corn oil, can also be extracted from wheat bran, certain fruits, and vegetables. It exhibits potent anti-inflammatory, antioxidant, and antitumor effects, in addition to lowering cholesterol levels, alleviating anxiety, and improving gastritis and diabetes. In the context of IVDD, gamma-oryzanol prevents ECM degradation and oxidative injury by disrupting IL-1β/NLRP3-mediated feedback via downregulation of NF-κB signaling and ROS accumulation^[113]^. Aucubin, widely distributed in the Plantaginaceae family and also present in Eucommiaceae, Caprifoliaceae, and Scrophulariaceae species, possesses diverse pharmacological properties, including damp-heat clearance, diuretic promotion, analgesia, antihypertensive effects, hepatoprotection, and antitumor activity. Preliminary studies indicate that it delays IVDD by inhibiting NPCs (Supplementary Digital Content Table 1, available at: http://links.lww.com/JS9/F228)^[114]^ and mitigates cartilage endplate degeneration in IVDD by suppressing NF-κB/NLRP3 inflammasome activation in CEPCs^[115]^. Sinapine thiocyanate, an alkaloid extracted from seeds of the Brassicaceae family, exhibits anti-inflammatory, antioxidant, and antitumor effects. Studies show that it inhibits pyroptosis by downregulating LPS-induced NLRP3, IL-1β, and IL-18 expression in NPCs^[116]^. Unlike Sinapine thiocyanate, magnoflorine inhibits pyroptosis through M1 macrophage polarization. Treating LPS-induced Tohoku Hospital Pediatrics-1 (THP1) cells with magnoflorine and using their conditioned medium for NPCs treatment revealed that magnoflorine suppresses HMGB1 expression in NPCs while inactivating the MyD88/NF-κB pathway and the NLRP3 inflammasome^[117]^.Maltol suppresses ECM degradation and inflammatory responses by inhibiting NLRP3 inflammasome-mediated pyroptosis via the PI3K/AKT/NF-κB signaling pathway^[118]^. Found in larch bark, pine needles, and food products such as roasted malt, caramel, roasted coffee, cocoa, and bread crust, maltol is an approved food flavoring. Its cost-effectiveness, availability, and lack of toxic side effects make it a promising candidate for further development. Morin mitigates NPC pyroptosis and improves IVD degeneration by inhibiting the TXNIP/NLRP3/Caspase-1/IL-1β signaling pathway^[119]^. Another promising application of natural products involves novel targeted drug delivery systems that combine bioactive compounds with nanomaterials. Bao *et al*^[120]^ developed a fibrin glue-based delivery system incorporating rhein and experimentally demonstrated its efficacy in ameliorating LPS-induced inflammatory microenvironments, regulating ECM metabolic disorders, and inhibiting NLRP3 inflammasome aggregation and pyroptosis in NPCs *in vitro*. However, no studies have yet explored natural products targeting pyroptosis in AFCs to delay IVDD.

Pyroptosis, also known as inflammatory cell necrosis, is a pro-inflammatory form of PCD that plays a key role in inflammatory responses. In this study, we identified 37 natural compounds – including myricetin, evodiamine, and ganoderic acid A – that inhibit inflammatory cell death. However, because these studies did not explicitly assess pyroptosis levels, a detailed discussion of these compounds is omitted from the main text. A comprehensive list of these compounds and their regulatory mechanisms is provided in supplementary materials (Supplementary Digital Content Table 2, available at: http://links.lww.com/JS9/F229).

## Natural products targeting necroptosis for IVDD

### Necroptosis

Traditional academic perspectives considered cell necrosis to be an uncontrolled form of cell death. However, in 2005, Professor Yuan and her team identified a novel cell death modality in a mouse cerebral ischemia model that exhibited features of both classical necrosis (e.g., plasma membrane rupture and release of cellular contents) and apoptosis (via regulated signaling pathways)^[121]^. They termed this process “necroptosis.” Necroptosis is the first PCD mechanism known to mediate necrosis. Upon activation of death receptors or other inducers, cells undergo osmolytic edema characterized by cellular swelling, cytoplasmic vacuolization, and plasma membrane blebbing. This is accompanied by increased mitochondrial membrane permeability, with blurred cristae and partial mitochondrial rupture, as well as transient dilation and subsequent fragmentation of the rough endoplasmic reticulum, lysosomal rupture, and the formation of membrane pores that ultimately compromise membrane integrity. The release of intracellular contents subsequently triggers a robust inflammatory response^[122]^.

**Figure 4.**
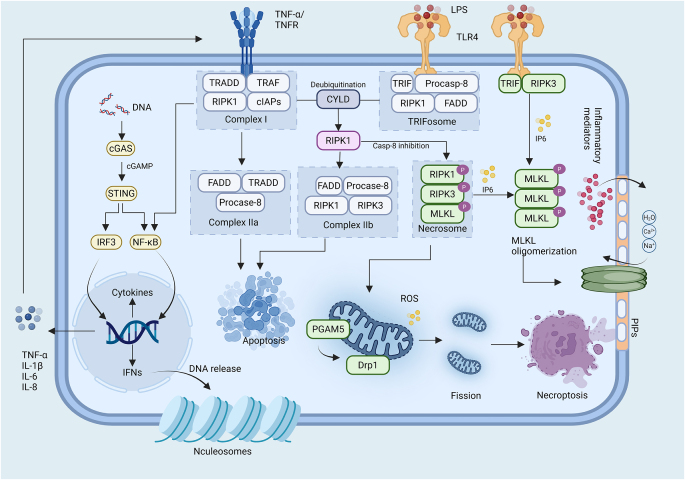
Molecular mechanisms of necroptosis. Created in BioRender (2025) https://BioRender.com/vzhd341.

The core mechanism of necroptosis lies in activation of the RIPK1/ RIPK3/MLKL pathway (Fig. 4). Various stimuli activate death receptors such as TNFR and Fas, as well as TLR3 and TLR4. Among these, the TNF/TNFR1 pathway is the most extensively studied classical necroptosis pathway^[123]^. Upon TNFR1 activation, conformational changes trigger TNFR1 trimerization, exposing the DD, which facilitates the recruitment of RIPK1, TRADD, IAPs, TRAF2, and TRAF5, forming Complex I. Within this complex, RIPK1 is ubiquitinated by IAPs, rendering it inactive while simultaneously activating pro-inflammatory downstream effects via the NF-κB signaling pathway^[124,125]^. When cells receive a “death signal,” RIPK1 undergoes deubiquitination by cylindromatosis, which limits sustained NF-κB activation and redirects the pathway toward cell death. In this context, RIPK1, FADD, and procaspase-8 form Complex IIa, which activates downstream caspase cascades via caspase-8 to initiate apoptosis. In the absence or inactivation of caspase-8, RIPK1 becomes phosphorylated, exposing its RHIM (RIP homotypic interaction motif), which recruits RIPK3 to form the RIPK1–RIPK3 complex^[126]^. This complex subsequently recruits and phosphorylates MLKL. In the presence of inositol hexaphosphate (IP6), phosphorylated MLKL oligomerizes to form Complex IIb, known as the necrosome^[127]^. The MLKL oligomers then translocate to phosphatidylinositol phosphate (PIP)-enriched membrane domains, where they assemble into large pores. These pores facilitate the uncontrolled influx of ions, resulting in cell swelling, membrane rupture, and the indiscriminate release of intracellular contents, which are characteristic features of necroptosis^[128]^.

### Necroptosis in IVDD

Necroptosis has been identified as a significant driver of IVDD. Double immunofluorescence staining of RIP3/MLKL and RIP3/p-MLKL in degenerated and normal intervertebral discs from discectomy patients demonstrated a significant increase in the expression of RIPK3 and MLKL in the degenerated discs compared to the healthy ones^[129]^. Necroptosis in NPCs is crucial in IVDD progression, with current research primarily targeting these cells. Abnormal mechanical loading, particularly sustained compression, is a major cause of NPC necroptosis. Studies indicate that, under sustained compression, the expression levels of necroptosis-related proteins—RIPK1, RIPK3, and MLKL—are markedly elevated. Additionally, the upregulation of HSP90 has been linked to compression-induced NPC death. Inhibiting HSP90 can modulate the expression and activity of the RIPK1/RIPK3/MLKL signaling pathway, thereby significantly reducing compression-induced necroptosis in NPCs^[130]^. Mitochondrial dysfunction, caused by excessive ROS accumulation, is another significant factor driving NPC necroptosis. This process is potentially regulated by proteins such as Drp-1, the MyD88 complex, and the PARP-AIF pathway^[129,131,132]^. To further validate the roles of RIPK1, RIPK3, and MLKL in NPC necroptosis, Chen *et al*^[133]^ demonstrated that the use of specific inhibitors – Nec-1 (RIPK1 inhibitor), GSK’872 (RIPK3 inhibitor), and NSA (MLKL inhibitor) – significantly reduced necrotic changes and cell death in NPCs. Nec-1 blocked the RIPK1/RIPK3/MLKL pathway in a dose- and time-dependent manner, leading to a decrease in necroptosis. Further validation using RIPK1, RIPK3, and MLKL siRNA showed that cell viability improved significantly in the RIPK3-siRNA and MLKL-siRNA groups. Interestingly, the RIPK1-siRNA group exhibited increased cell mortality, suggesting that inhibiting RIPK1 expression may not prevent necroptosis – possibly due to reduced NF-κB pathway activation. This apparent contradiction with the core mechanism of necroptosis requires further investigation.

Fan *et al*^[134]^ reported significantly elevated expression levels of RIPK3, MLKL, and pro-MLKL in AFCs, indicating that these cells might be involved in the pathological process of IVDD through necroptosis. However, the specific mechanism remains to be elucidated. Currently, no mechanistic studies have addressed necroptosis in CEPC, which may represent potential therapeutic targets for IVDD treatment.

### The regulation of necroptosis by natural products

Compared to apoptosis and pyroptosis, necroptosis in NPCs remains relatively understudied in the context of IVDD. Following rigorous screening, only one compound, limonin, was selected for investigation (Table 4). Gong *et al*^[135]^ established both an IL-1β-induced inflammatory cell model and a lumbar instability model (induced by spinous process resection) to evaluate the therapeutic effects of limonin. The study demonstrated that limonin delayed the progression of IVDD in the lumbar instability model. Mechanistically, it suppressed the production of pro-inflammatory mediators such as iNOS and COX-2, while downregulating key components of the MAPK/NF-κB and RIPK1/RIPK3/MLKL pathways in NPCs, and inhibited NPC necroptosis. These findings suggest that limonin’s ability to inhibit NPC necroptosis may represent a promising direction for future research in delaying IVDD progression.

**Table 4:** Natural products targeting necroptosis for IVDD treatment

	Natural products	Chemical class	Sources	Structure	Median lethal dose (Mice)	Safety	Target cell types	Necroptosis-related mode of action	Therapeutic dose	Key regulatory mechanisms	References
1	Limonin	Terpenoids	Raulinoa echinata, Citrus tankan	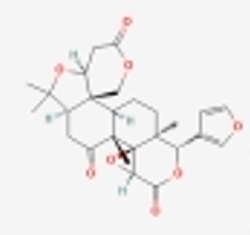	Unknown	Causes skin and serious eye irritation	NPC	RIPK1, RIPK3, MLKL	50 μM (*In vitro*)	Inhibition of MAPK/NF-κB pathway and RIP1/RIP3/MLKL activation suppresses necroptosis inNPCs.	^[135]^
									20 mg/kg/w (*In vivo*/Mice/Intraperitoneal injection)		

## Natural products targeting ferroptosis for IVDD

### Ferroptosis

In the 1970s, researchers observed that removing cysteine – a sulfur-containing amino acid – from the culture medium induced a nonapoptotic form of cell death. However, due to technological and conceptual limitations at the time, the underlying mechanism remained unclear^[136]^. It wasn’t until 2003 that Dolma *et al*^[137]^ identified *erastin*, a compound capable of selectively killing RAS-mutant cancer cells, which initiated a new wave of inquiry. This cell death mode lacked hallmarks of apoptosis – such as nuclear condensation, DNA fragmentation, and caspase activation – and could not be reversed by caspase inhibitors. Subsequent investigations revealed that iron chelators could suppress this process, while agents like RSL3 were capable of triggering it^[138,139]^. In 2012, Brent R. Stockwell and colleagues formally designated this new mode of cell death as ferroptosis, distinguishing it from apoptosis, necrosis, and other traditional forms^[140]^. Although initially classified as morphologically distinct from other death types, ferroptotic cells often exhibit necrosis-like characteristics – such as plasma membrane rupture, cytoplasmic and organelle swelling, and partial chromatin condensation^[141]^. At the same time, ferroptosis displays unique characteristics, including mitochondrial shrinkage, increased membrane density, and reduced or absent mitochondrial cristae^[139]^. While early research on ferroptosis focused on mammalian systems, subsequent studies have expanded its scope, revealing that ferroptosis also occurs in evolutionarily distant species, including plants, protists, and fungi^[142,143]^. Today, ferroptosis is widely acknowledged as a highly conserved and evolutionarily ancient mode of RCD.

**Figure 5.**
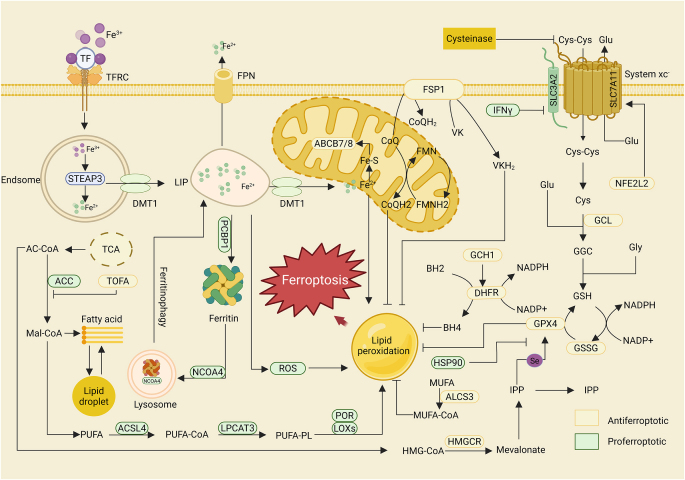
Molecular mechanisms of ferroptosis. Created in BioRender (2025) https://BioRender.com/z00b9hj.

Ferroptosis is a regulated, iron-dependent form of cell death that is principally driven by the accumulation of lipid peroxides (Fig. 5). Its initiation and progression are intricately governed by iron and lipid metabolic pathways, alongside a network of regulatory signaling cascades. Iron redox cycling and uptake are critical initiating steps. Extracellular Fe^3^⁺ binds to TF and is internalized via TFRC-mediated endocytosis, a process facilitated by actin cytoskeleton dynamics that promotes Fe^3^⁺ transmembrane transport and intracellular accumulation^[144]^. Meanwhile, PKC-mediated HSPB1 inhibits cytoskeletal function, thereby reducing iron uptake^[145]^. The balance between the TF–Fe^3^⁺–TFRC–actin axis (positive regulation) and the PKC–HSPB1 signaling pathway (negative feedback) maintains iron homeostasis. Once internalized, Fe^3^⁺ is reduced to ferrous iron (Fe^2^⁺) by the metalloreductase STEAP3 and transported into the cytosol via the divalent metal transporter SLC11A2 (also known as DMT1), where it engages in diverse metabolic activities. Fe^2^⁺ functions both as a cofactor for iron-dependent enzymes (supporting cell survival) and as a redox regulator that triggers lipid peroxidation, thereby promoting ferroptosis. Fe–S cluster homeostasis is crucial in this process: disruption of Fe–S cluster synthesis due to NFS1 deficiency exacerbates ferroptosis, whereas ISCU overexpression mitigates cell death through compensatory synthesis^[146]^. Additionally, mitochondrial membrane proteins CISD1/CISD2 protect cells by restricting local iron availability^[147,148]^. Conversely, excessive Fe^2^⁺ buildup in lysosomes and the endoplasmic reticulum amplifies oxidative stress through Fenton chemistry. The highly sophisticated and precise iron metabolism system regulates intracellular iron levels, thereby modulating the onset of ferroptosis.

Lipid peroxidation represents a defining feature of ferroptosis, with lipid metabolism being regulated through a highly intricate, multitiered network. PUFAs containing bis-allylic groups – such as LA, AA, and their secondary products (e.g., AdA)^[149]^. Their release depends on PLA2 activity and the esterification process catalyzed by ACSL4 and LPCAT3^[150–152]^. In contrast, MUFAs exert antiferroptotic effects by competitively inhibiting PUFA oxidation and activating the ACSL3/SCD-1 pathway^[153]^. Lipid peroxidation in ferroptosis is regulated by multiple mechanisms. ALOX family proteins (ALOXE3, ALOX5, ALOX12, ALOX12B, ALOX15 and ALOX15B)^[154]^ induce ferroptosis by tissue-specific regulation of PUFA peroxidation to generate AA/AdA-PE-OOH^[155]^. Additionally, POR, together with its cofactors FMN and FAD, promotes PUFA peroxidation via NADPH, thereby accelerating ferroptosis^[156]^.

### Ferroptosis in IVDD

The role of ferroptosis in IVDD has been systematically elucidated through multidimensional studies. Its core mechanisms involve a complex network comprising iron metabolism imbalance, oxidative stress cascades, regulation of key signaling pathways, and responses to mechanical stress. During IVDD, intracellular iron overload triggers ferroptosis, which exhibits distinct molecular and pathological characteristics in NPCs, AFCs, and CEPCs.

Studies have shown that in degenerated nucleus pulposus tissues, iron ion concentrations are significantly elevated while the expression of GPX4 and FTH1 is markedly reduced, correlating positively with the degree of degeneration. This indicates that increased extracellular iron ions induce ferroptosis in NPCs^[157]^. NPCs are particularly vulnerable to mechanical stimuli, and Piezo1, a mechanosensitive ion channel, has emerged as a crucial iron transporter in this context. Unlike the classical TFRC-dependent pathway, Piezo1 mediates iron influx independently, thereby disrupting iron homeostasis and facilitating excessive ferroptosis. Inhibition or conditional knockout of Piezo1 effectively reduces iron accumulation, decreases mitochondrial ROS generation, and suppresses ferroptosis, alleviating mechanical injury-induced IVDD^[158]^. Excessive mechanical loading can also induce ERS in NPCs, which increases intracellular free Ca^2^⁺ levels via the Piezo1 channel. Calcium overload impairs GPX4 production and function, further promoting ferroptosis in NPCs. Selenium supplementation mitigates ERS by upregulating SelK expression, reducing intracellular free Ca^2^⁺ levels, and improving ferroptosis via the Se–GPX4 and Se–SelK axes, thereby stabilizing the ECM^[159]^. Moreover, promoting the expression of SLC7A11 to enhance GPX4 levels has been confirmed as an effective strategy to inhibit NPC death and improve IVDD^[160]^. Oxidative stress is a core driver of ferroptosis. Glutamine has been shown to alleviate this stress in NPCs by Nrf2 through deubiquitination, thus protecting against ferroptotic injury ^[161]^. The USP11 modulates oxidative stress-induced ferroptosis by stabilizing SIRT3^[162]^. In AFCs, oxidative stress-induced ferroptosis has also been documented. Yang *et al*^[163]^ demonstrated *in vitro* that TBHP induces ferroptosis in annulus fibrosus cells, suggesting that alleviating oxidative stress to inhibit ferroptosis may offer a potential therapeutic strategy for IVDD. Huo *et al*^[164]^ further identified MGST1 as a promising target for inhibiting ferroptosis in annulus fibrosus cells through bioinformatics analysis and experimental validation, highlighting its therapeutic potential for IVDD. Wang *et al*^[165]^. simulated iron overload in CEPCs using ferric ammonium citrate *in vitro* and found that excessive iron promoted pathological mineralization, while treatment with iron chelators, antioxidants, or ferroptosis inhibitors effectively mitigated these degenerative changes. Zhang’s group further demonstrated that EGR1 promotes ferroptosis and cartilage endplate degeneration via the MAPK–NF-κB signaling axis ^[166]^, and Jiang *et al*^[167]^ demonstrated that HIF-2α/TFR1-mediated disruption of iron homeostasis exacerbates cartilage endplate degeneration via ferroptosis-related damage and mitochondrial DNA release, thereby promoting IVDD progression

Unlike apoptosis, pyroptosis, and necroptosis, the roles of AFCs and CEPCs in ferroptosis are only beginning to attract research attention. As a relatively new cell death mechanism, the molecular pathways underlying ferroptosis remain insufficiently explored, marking it as a promising area for future IVDD research.

### The regulation of ferroptosis by natural products

The study included eight natural products targeting ferroptosis for the prevention and treatment of IVDD. Among these, six act on NPCs, two on CEPCs, and no experimental studies were found regarding natural products acting on AFCs (Table 5). The eight natural products comprise four flavonoids (fisetin, hesperidin, puerarin, and pinocembrin), two terpenoids (hinokitiol and astaxanthin), one alkaloid (tomatidine), and one phenolic acid (cynarin).

**Table 5:** Natural products targeting ferroptosis for IVDD treatment

	Natural products	Chemical class	Sources	Structure	Median lethal dose (Mice)	Safety	Target cell types	Ferroptosis-related mode of action	Therapeutic dose	Key regulatory mechanisms	References
1	Fisetin	Flavonoids	Strawberries, apples, persimmons, cucumbers, Onions	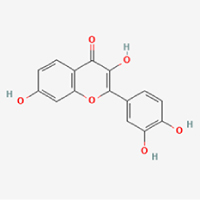	180 mg/kg (Intravenous)	Causes skin and serious eye irritation, May cause respiratory irritation	NPC	ACSL4, FTH, GPX4,	20 μM (*In vitro*)	Stimulation of the Nrf2/ HO-1 signaling cascade alleviates ferroptosis in NPCs triggered by oxidative stress.	^[172]^
									20 mg/kg/d (*In vivo*/Rat/Intragastrically)		
2	Hesperidin	Flavonoids	Humulus lupulus, Ficus erecta var. beecheyana	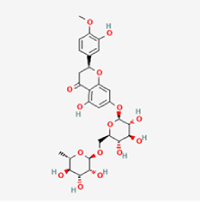	1 g/kg (Intraperitoneal)	Safety	NPC	ACSL4, FTH, GPX4, PTGS2	10 μM (*In vitro*)	Dual modulation of Nrf2 upregulation and NF-κB pathway inhibition suppresses oxidative stress-dependent ferroptosis.	^[173]^
									50 mg/kg/d (*In vivo*/Mice/Intragastrically)		
3	Puerarin	Flavonoids	Bupleurum chinense, Pueraria calycina	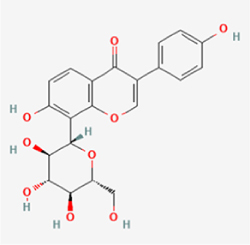	783 mg/kg (Intravenous)	Causes skin and serious eye irritation, May cause respiratory irritation	NPMSC	ACSL4, PTGS, GPX4	10 μM (*In vitro*)	Modulation of LINC01534 suppresses ferroptosis, thereby attenuating inflammatory responses and ECM degradation.	^[175]^
4	Tomatidine	Alkaloid	Solanum tuberosum, Solanum kieseritzkii	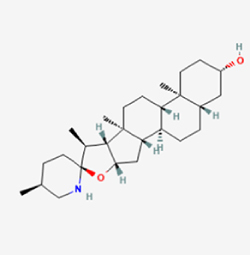	>100 mg/kg (Oral, Red-winged blackbird)	Toxic if swallowed, Harmful if swallowed, Toxic if inhaled	NPC	GPX4, GSH, FTL, TRF	4 μM (*In vitro*)	Activation of the Nrf2/HO-1/GPX4 pathway inhibits ferroptosis in NPCs.	^[180]^
									10 mg/kg/w (*In vivo*/Mice/Intraperitoneal injection)		
5	Hinokitiol	Terpenoids	Thujopsis dolabrata, Chamaecyparis formosensis	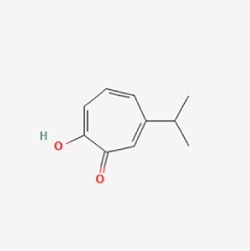	396 mg/kg (Oral)	Harmful if swallowed	NPC	TFRC, ROS, FTL	8 μM (*In vitro*)	Inhibition of the JNK pathway enhances nuclear translocation of MTF1 and ameliorates IVDD progression *in vivo*.	^[181]^
									150 mg/kg/d (*In vivo*/Rat/Intraperitoneal injection)		
					781 mg/kg (Oral, Rat)						
6	Cynarin	Phenolic Acids	Echinacea angustifolia, Solanum tuberosum	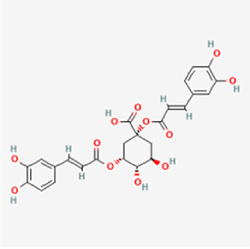	Unknown	Safety	NPCs	GPX4, NRF2	50 μM (*In vitro*)	Upregulation of GPX4 expression attenuates intracellular Fe^2^⁺ accumulation, curbs the build-up of lipid peroxides and ROS.	^[178]^
									50 μM/2 μl/qow (*In vivo*/Rat/Intradiscal injection)		
7	Astaxanthin	Terpenoids	Agrobacterium aurantiacum, Phaffia rhodozyma	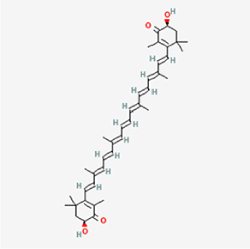	>2000 mg/kg (Oral, Rat)	May cause long lasting harmful effects to aquatic life	CEPC	GPX4, FTH, SLC7A11	20 μM (*In vitro*)	Enhancing Nrf2/HO-1 signaling facilitates mitophagy, alleviates oxidative stress, and suppresses ferroptosis in CEP chondrocytes.	^[182]^
									30 mg/kg/qod (*In vivo*/Mice//Intraperitoneal injection)		
8	Pinocembrin	Flavonoids	Apis, Populus yunnanensis	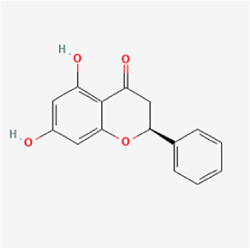	>1500 mg/kg (Oral)	Safety	CEPC	GPX4, SLC7A11, FTH1	50 μM (*In vitro*)	Activation of Nrf2-mediated mitophagy suppresses ferroptosis through coordinated mitochondrial quality control and iron homeostasis regulation.	^[183]^
									20 mg/kg/d (*In vivo*/Mice//Intraperitoneal injection)		

Fisetin, a natural antioxidant widely found in fruits and vegetables such as strawberries and apples, exhibits excellent anti-aging properties^[168]^ and has shown significant therapeutic potential in kidney diseases, neurological disorders, and cancer^[169–171]^. Hesperidin, a flavonoid present in citrus peels, displays various biological activities, including the reduction of inflammatory mediator expression and antioxidant effects. Its mechanism is similar to that of fisetin; both inhibit ferroptosis in NPCs by regulating Nrf2 to improve oxidative stress. However, fisetin acts downstream via HO-1^[172]^, whereas hesperidin signals through NF-κB^[173]^. Puerarin, a flavonoid isolated from kudzu root (*Pueraria lobata*), has been shown to treat IVDD by inhibiting apoptosis in NPCs^[174]^. Cui *et al*^[175]^ further validated its regulatory effect on NPMSCs, demonstrating that puerarin significantly promotes NPMSC proliferation, suppresses the expression of COX-2 and the pro-inflammatory cytokine IL-6, and inhibits ferroptosis by modulating key genes (ACSL4 and GPX4). Cynarin possesses free radical scavenging and antioxidant properties, along with cholagogic, hepatoprotective, and cholesterol-lowering effects, and is clinically used to treat liver insufficiency^[176]^. Zhang *et al* showed that cynarin inhibits microglia-induced neuroinflammation after spinal cord injury via the Nrf2/ROS/NLRP3 axis^[177]^. Further research indicates that cynarin can prevent and treat IVDD by inhibiting ferroptosis in NPCs^[178]^. Research targeting CEPCs for IVDD treatment is limited, with only two natural products identified: astaxanthin and pinocembrin. Astaxanthin, a potent antioxidant carotenoid found in shrimp, crabs, fish, birds, certain algae, and fungi, exhibits antioxidant, anti-aging, and antitumor effects, and is used in health foods, high-end cosmetics, and pharmaceuticals. However, its low stability – due to susceptibility to oxidation and photodegradation – necessitates its formulation in gel form to preserve activity. In contrast, pinocembrin, primarily obtained from cherry tree heartwood, Swiss stone pine, and propolis, is relatively scarce. Both astaxanthin and pinocembrin regulate ferroptosis in CEPCs via the Nrf2 signaling pathway, thereby contributing to the prevention and treatment of IVDD. In addition to directly isolated natural products, Zhang *et al*^[179]^ investigated their derivatives. The study found that nordihydroguaiaretic acid ameliorates IVDD by inhibiting ferroptosis. This discovery offers new perspectives and directions for the clinical application and innovative development of natural products.

Targeting ferroptosis in IVD cells has become an emerging focus in the field of degenerative disc disease research. Increasing evidence highlights the regulatory potential of natural compounds in modulating ferroptosis, offering promising avenues for the development of novel therapeutic agents for IVDD.

## Natural products targeting autophagy for IVDD

### Autophagy

The history of cellular autophagy dates back to 1963, when C. de Duve observed double-membrane vesicles in eukaryotic cells encapsulating intracellular components for lysosomal degradation, a phenomenon he later termed “autophagy.” However, due to the limitations of molecular biology techniques at the time, research on autophagy was restricted to morphological observations. In 1992, Japanese scientist Yoshinori Ohsumi used baker’s yeast as a model system to directly observe autophagosome formation under starvation conditions and cloned the first autophagy-related gene, autophagy-related gene 1 (Atg1), thereby opening the door to molecular autophagy research^[184]^. Subsequent investigations have classified autophagy into three subtypes based on substrate transport mechanisms: macroautophagy, microautophagy, and chaperone-mediated autophagy. Macroautophagy is the most widely studied form and involves the formation of double-membrane autophagosomes that mature and fuse with lysosomes, resulting in autolysosomes where the degradation of intracellular contents occurs. Microautophagy, by contrast, involves direct engulfment of cytoplasmic components through invagination or protrusion of the lysosomal membrane. Chaperone-mediated autophagy does not involve vesicle formation; instead, it facilitates the translocation of unfolded proteins directly across the lysosomal membrane for degradation^[185]^.Among these, macroautophagy is the predominant form, regulated by multiple signaling pathways, and is capable of both bulk degradation of cytoplasmic components and selective clearance of specific substrates, such as damaged mitochondria and misfolded proteins. Consequently, macroautophagy is the central focus of autophagy research and the primary direction of this study.

Macroautophagy is executed through a highly coordinated, multistage process comprising four distinct phases: initiation, autophagosome formation, autophagosome–lysosome fusion, and cargo degradation^[186]^. During the initiation phase, the ULK1/2 kinase complex, which consists of ULK1/2, ATG11, ATG13, and ATG101, is activated in response to nutrient deprivation or stress signals. Under such conditions, mTORC1 activity is inhibited, resulting in the dephosphorylation and activation of the ULK1/2 complex, which then translocates to the ER or other membrane compartments to initiate autophagy. In parallel, AMPK can directly phosphorylate and activate the ULK1/2 complex independently of mTORC1. Additionally, damaged mitochondria and other stimuli can activate the ULK1/2 complex via ATG11^[187]^. Subsequently, ULK1/2 phosphorylates the class III PI3K complex – comprising ATG14, Beclin 1, VPS34, and VPS15 – thereby catalyzing the production of PI3P and promoting nucleation of the autophagosome precursor membrane. ATG9 vesicles provide membrane sources and contribute to membrane elongation^[188]^. PI3P recruits WIPI family proteins (e.g., WIPI2) and lipid transfer proteins ATG2A/B, facilitating membrane expansion. WIPI2, recruits the ATG12–ATG5–ATG16L1 complex, promoting the conjugation of ATG8 family proteins, such as microtubule-associated LC3, with PE to form lipidated LC3-II, a hallmark of autophagosome membranes^[189]^. Finally, the endosomal sorting complex required for transport mediates membrane closure, completing autophagosome formation^[190]^.Mature autophagosomes fuse with lysosomes through interactions between soluble NSF attachment protein receptors (such as Syntaxin 17 and yeast karyopherin-like transport protein 16) and lysosomal proteins such as vesicle-associated membrane protein 7and 8, and Syntaxin 17, with the homotypic fusion and protein sorting complex, ectopic P-granules 5 autophagy tethering factor, and proline-and tyrosine-rich kinase substrate 1 facilitating the process^[191]^. Moreover, selective degradation relies on direct recognition by ATG8 proteins or on the recruitment of ubiquitinated substrates by adaptor proteins such as P62 and NDP52. For instance, damaged mitochondria labeled by the PINK1/Parkin pathway are specifically targeted for degradation through ubiquitination^[192]^. Precise regulation of these processes is essential for maintaining cellular homeostasis.

For a long time, autophagy has been a controversial topic. Many researchers consider autophagy a metabolic process in which cells degrade damaged components via lysosomes for recycling, thereby maintaining intracellular homeostasis and adapting to environmental stress^[193]^. Essentially, it functions as the cell’s “production-promoting mechanism.” However, substantial evidence indicates that autophagy also serves as a multifaceted regulator of cell death. Consequently, autophagy-related cell death is primarily classified into two categories: ADCD and AMCD^[194,195]^. In 2018, the NCCD defined RCD in its complete physiological form as PCD and formally incorporated ADCD into the scope of RCD^[196]^. Attributing cell death to ADCD imposes stringent criteria: (1) autophagic flux must increase during the the process of cell death; (2) inhibition of autophagy by genetic or pharmacological means must render the cell death process reversible; (3) the process must involve at least two autophagy-related genes or proteins, ensuring that no single molecule alone can mediate cell death; and (4) the process must be independent of other known cell death pathways. Whether ADCD represents an entirely independent type of PCD remains a subject of debate^[197]^. To ensure the comprehensiveness of our study and to identify additional natural products capable of targeting IVDD for therapeutic purposes, we have included all natural products that can target and inhibit cellular autophagy. This approach aims to provide drug developers with a broader range of candidate compounds and new research directions.

### Autophagy in IVDD

IVD cells are continuously exposed to multiple stressors – including nutrient deprivation, hypoxia, mechanical stress, and accumulated damage – that create a microenvironment similar to laboratory conditions used to induce autophagy artificially. This “natural stress combination” has positioned autophagy as a focal point in IVD cell biology research. Emerging evidence indicates that autophagy plays a crucial role in the pathogenesis of IVDD. In particular, dysregulated autophagy and impaired nutrient metabolism in IVD cells have been identified as key contributors to the development and progression of IVDD^[198–200]^. Nevertheless, the precise role of autophagy in IVDD remains a topic of debate, with studies reporting both protective and detrimental effects. Given the current paucity of research on autophagy in AFCs and CEPCs, the present study focuses primarily on NPCs.

Autophagy exhibits a dual role in IVDD. Some researchers argue that excessive autophagy may contribute to the pathological progression of IVDD. Under prolonged extreme stress, overactivation of autophagy can shift toward pathological processes, leading to autophagic cell death and thereby accelerating IVDD. Numerous studies support this conclusion. Numerous studies support this conclusion. Evidence indicates that autophagy marker expression in IVD cells increases with the degree of degeneration. In human degenerated IVD tissues, autophagy-related genes – such as Beclin-1, Atg8, Atg12, Cathepsin B, and Presenilin-1 – are significantly upregulated compared to healthy discs^[201]^. Animal studies reveal similar findings; with aging, rat IVD tissues display increased autophagic vacuoles and elevated expression of LC3-II and LAMP-2A^[202]^. In moderately degenerated human discs, the expression and phosphorylation levels of mTOR, a key negative regulator of autophagy, decrease with age^[203]^. Moreover, autophagy may exacerbate IVDD by interacting with other cell death pathways, such as pyroptosis and apoptosis. For instance, Sun et al.^[204]^ demonstrated that LncRNA H19 promotes both autophagy and apoptosis in nucleus pulposus cells via the miR-139/NF-κB axis, thereby worsening disc degeneration. Additionally, ERS mediates crosstalk between autophagy and apoptosis through ROS-dependent pathways, further aggravating IVDD^[205]^. Collectively, these findings suggest that autophagy may have deleterious effects in IVDD, and its widespread activation could accelerate disc cell apoptosis and senescence.

Recent investigations have challenged earlier assumptions by proposing that the induction of cellular autophagy plays a protective role in IVDD by exerting its effects through multiple pathways. For instance, cartilaginous endplate-derived stem cells have been shown to attenuate disc degeneration through the secretion of exosomes that trigger the Akt-mediated autophagic response in NPCs^[206]^. Glutamine promotes autophagy and suppresses NPC senescence by reducing AMPKα lactylation via glycolysis inhibition, thereby preventing IVDD^[207]^. Additionally, Mitofusin 2 overexpression enhances ROS-dependent mitophagy through the PINK1/Parkin pathway, effectively mitigating IVDD^[208]^. As a result, improving cellular autophagy has gradually emerged as the mainstream strategy for suppressing IVDD.

Based on the “pro-survival nature” of autophagy and its potential role in “autophagy-dependent cell death,” we hypothesize that autophagy may exhibit a stage-dependent, double-edged sword effect in IVD degeneration. In the early stages, autophagy is protective by maintaining metabolic homeostasis and clearing damaged components, whereas in later stages, dysfunctional or excessive autophagy may exacerbate pathology. This hypothesis, however, requires further experimental validation.

### The regulation of autophagy by natural products

The study analyzed 21 articles on the prevention of IVDD via cellular autophagy and identified 19 natural compounds as potential candidate drugs. These include taurine, quercetin, apigenin, naringin, and resveratrol. Flavonoids emerged as the predominant category with eight compounds, followed by terpenoids (three compounds), lignans (two compounds), alkaloids (two compounds), and one compound each from the stilbenes, amino acids, gingerols, and diarylheptanoids groups Table 6). Unlike other forms of PCD, autophagy exhibits unique characteristics. Functionally, autophagy is primarily an adaptive survival mechanism that degrades damaged organelles, misfolded proteins, and pathogens to recycle resources and maintain metabolic homeostasis, whereas PCD (e.g., apoptosis) actively eliminates abnormal cells via genetic regulation to preserve tissue integrity. Consequently, autophagy interacts in complex and dynamic ways with other PCD pathways (e.g., apoptosis, necroptosis, pyroptosis), exhibiting both cooperative and antagonistic effects that collectively maintain cellular homeostasis or drive pathological progression.

**Table 6:** Natural products targeting autophagy for IVDD treatment

	Natural products	Chemical class	Sources	Structure	Median lethal dose (Mice)	Safety	Target cell types	Autophagy -related mode of action	Therapeutic dose	Key regulatory mechanisms	References
1	Taurine	Amino Acids	marine fish, shellfish	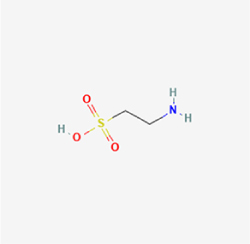	6630 mg/kg (Intraperitoneal)	Causes skin and serious eye irritation, May cause respiratory irritation	NPC	PINK1, Parkin, LC3	0.3 mM/2 μl (*In vivo*/Rat/Intradiscal injection)	Activation of PINK1/Parkin-mediated mitophagy ameliorates mitochondrial homeostasis.	^[209]^
					6 g/kg (Subcutaneous)						
					>7 g/kg(Oral)						
					>5 g/kg(Oral, Rat)						
2	Procyanidin C1	Flavonoids	Camellia sinensis, Camellia reticulata	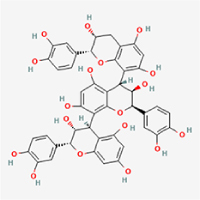	Unknown	Unknown	NPC	PINK1, Parkin, LC3	40 μM (*In vitro*)	Modulation of the SIRT3/FOXO3 signaling pathway regulates mitochondrial homeostasis to ameliorate IVDD.	^[210]^
									8 mg/kg/d (*In vivo*/Rat/Intraperitoneal injection)		
3	Andrographolide	Terpenoids	Andrographis paniculata, Cymbopogon schoenanthus	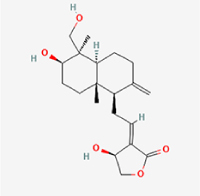	11 460 mg/kg (Intraperitoneal)	Causes skin and serious eye irritation, May cause respiratory irritation	NPC	PINK1, Parkin, LC3	25 μM (*In vitro*)	Activation of the miR-9/FoxO3/PINK1/Parkin axis promotes proliferation and autophagy flux in NPCs, while suppressing apoptosis and oxidative stress.	^[211]^
									150 mg/kg/d (*In vivo*/Rat/Intragastrically)		
4	Apigetrin	Flavonoids	Acanthus ebracteatus, Coreopsis lanceolata	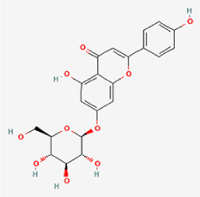	Unknown	Unknown	NPC	P62, ATG5, ATG12, LC3	50 μM (*In vitro*)	Inhibition of the NF-κB and AMPK signaling pathways helps reduce inflammatory responses, whereas activation of the PI3K/AKT/mTOR pathway promotes increased autophagic flux.	^[214]^
									50 μM/2 μl (*In vivo*/Rat/Intradiscal injection)		
5	Myricetin	Flavonoids	Caragana frutex	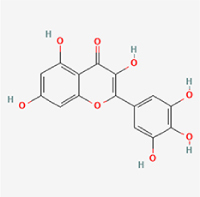	Unknown	Causes skin and serious eye irritation, May cause respiratory irritation	NPC	LC3, Beclin-1, P62	28 μM (*In vitro*)	Blockade of the JAK2/STAT3 signaling pathway enhances autophagy flux, thereby attenuating apoptosis and ECMdegradation in NPCs.	^[219]^
									20 mg/kg/d (*In vivo*/Rat/Intraperitoneal injection)		
6	Isoginkgetin	Flavonoids	Podocarpus neriifolius, Sequoiadendron giganteum	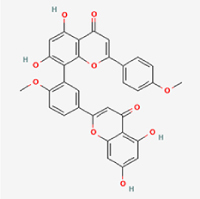	Unknown	Unknown	NPC	LC3, Beclin-1, ATG7, P62	/	Enhancement of autophagy flux in NPCs suppresses ECM degradation and attenuates apoptosis.	^[220]^
7	Scutellarin	Flavonoids	Perilla frutescens, Scutellaria indica	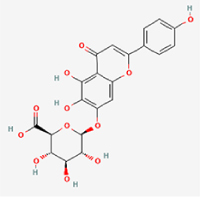	1314 mg/kg (Intravenous)	Unknown	NPC	LC3, ATG7, P62, ATG5	20 μM (*In vitro*)	Targeting the PI3K/PTEN/Akt signaling axis activates autophagy flux in NPCs, upregulates Rab8A-mediated exosomal secretion, and pioneers a novel therapeutic strategy for IVDD prevention.	^[221]^
8	Achyranthoside D	Terpenoids	Beta vulgaris, Achyranthes bidentata	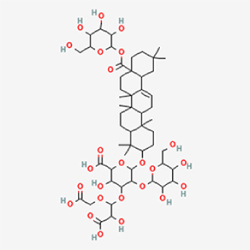	Unknown	Unknown	NPC	LC3, Beclin-1, P62	35 μM (*In vitro*)	Modulation of the PI3K/Akt/mTOR signaling axis enhances autophagy flux to ameliorate IVDD.	^[222]^
									240 μg/g/d (Rat)		
9	Apigenin	Flavonoids	Camellia sinensis, Apis	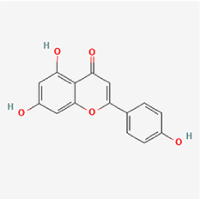	Unknown	Causes skin and serious eye irritation, May cause respiratory irritation	NPC	LC3, P62, LAMP2	50 μM (*In vitro*)	Restoring autophagic flux helps protect NPCs from apoptosis, senescence, and ECM degradation induced by TBHP.	^[213]^
									10 mg/kg/d (*In vivo*/Rat/Intraperitoneal injection)		
10	Naringin	Flavonoids	Salvia officinalis, Citrus reticulata	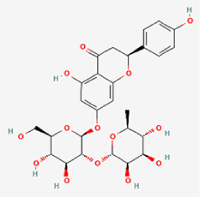	2 g/kg (Rat:Intraperitoneal)	Causes skin and serious eye irritation. May cause respiratory irritation	NPC	LC3, Beclin-1, P62, Beclin-2	17 μM (*In vitro*)	AMPK-mediated activation of SIRT1 promotes autophagic flux, thereby protecting NPCs from inflammatory responses, oxidative stress, and disruptions in cellular homeostasis.	^[223]^
11	Quercetin	Flavonoids	Tea, tomatoes, cherries, grapes, apples,onions	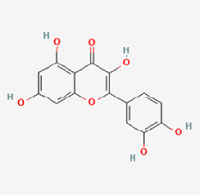	3 g/kg (Intraperitoneal)	Toxic if swallowed	NPC	LC3, Beclin-1, P62,	25 μM (*In vitro*)	Activation of the p38 MAPK-mediated autophagic pathway reduces apoptosis in NPCs and alleviates ECM degeneration.	^[216]^
									(*In vivo*/Rat/Intraperitoneal injection)		
					18 mg/kg (Intravenous)						
					97 mg/kg (Subcutaneous)						
					159 mg/kg(Oral)						
12	Sinomenine	Alkaloids	Stephania cephalantha, Sinomenium acutum	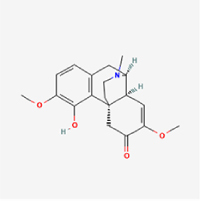	285 mg/kg (Intraperitoneal)	Harmful if swallowed, Causes skin and serious eye irritation. May cause respiratory irritation	NPC	Beclin-1, ATG5,	3.33 mM (*In vitro*)	Sinomenine mitigates TBHP-induced growth inhibition and apoptosis in NPCs by promoting autophagic flux, both in vitro and in vivo.	^[224]^
									75 mg/kg/d (*In vivo*/Rat/Intraperitoneal injection)		
					580 mg/kg (Oral)						
13	Moracin M	Lignans	Morus lhou, Morus cathayana	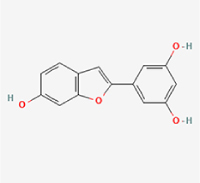	Unknown	Unknown	NPC	LC3, Beclin-1	20 μM (*In vitro*)	Inhibition of LPS-induced PI3K/Akt phosphorylation promotes autophagy flux in NPCs while suppressing pro-inflammatory mediator production.	^[225]^
14	Resveratrol	Stilbenes	Polygonum cuspidatum	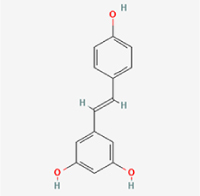	2 g/kg (Rat:Oral)	Causes skin and serious eye irritation. May cause respiratory irritation	NPC	LC3, P62	50 μM (*In vitro*)	Enhancement of autophagy flux alleviates oxidative stress-associated mitochondrial dysfunction and apoptosis, thereby ameliorating IVDD progression.	^[218]^
15	Resveratrol	Stilbenes	Polygonum cuspidatum	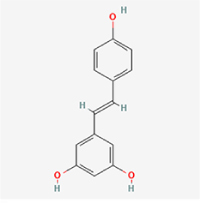	2 g/kg (Rat:Oral)	Causes skin and serious eye irritation. May cause respiratory irritation	NPC	LC3, Beclin-1	24 μM (*In vitro*)	Activation of the AMPK/SIRT1 signaling axis enhances autophagy flux, thereby suppressing TNF-α-induced MMP-3 expression in HNPCs.	^[217]^
16	Panax notoginseng saponins	Terpenoids	Panax ginseng, Panax japonicus	/	Unknown	Unknown	NPC	LC3, ATG7, P62	/	Activating the Akt/mTOR pathway inhibits cellular autophagy, thereby alleviating apoptosis of NPCs	^[226]^
17	Berberine	Alkaloids	goldenseal, barberry, Oregon grape	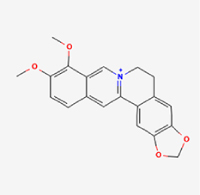	329 mg/kg (Oral)	Decreased fetal weight (Maybe)	NPC	ATG7, P62, LC3, Beclin-1	25 μM (*In vitro*)	Inducing autophagy acts as a protective mechanism, preventing NPC apoptosis and ECM degeneration.	^[227]^
					18 mg/kg (Intraperitoneal)				(*In vivo*/Rat/Intragastrically))		
18	6-gingerol	Gingerols	Cuminum cyminum, Aframomum melegueta	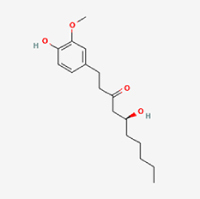	58 100 μg/kg (Intraperitoneal)	Unknown	NPMSC	LC3, Beclin-1,P62	30 μM (*In vitro*)	Activation of the PI3K/AKT signaling pathway enhances autophagic flux and activates autophagy, thereby inhibiting H₂O₂-induced apoptosis in NPMSCs.	^[228]^
					25 500 μg/kg (Intravenous)						
					250 mg/kg (Oral)						
19	Curcumin	Diarylheptanoids	Curcuma longa	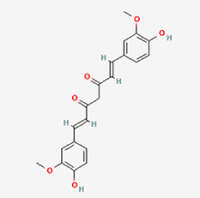	1500 mg/kg (Intraperitoneal)	Causes skin and serious eye irritation. May cause respiratory irritation	CEPC	LC3, Beclin-1	20 μM (*In vitro*)	Augmentation of autophagy flux enhances the adaptability of CEPCs to high-magnitude tensile stress, thereby ameliorating IVDD.	^[229]^
									50 mg/kg/d (*In vivo*/Rat/Intragastrically)		
					>2 g/kg (Oral)						
					>2 g/kg (Oral, Rat)						
20	Naringin	Flavonoids	Salvia officinalis, Citrus reticulata	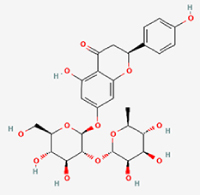	2 g/kg (Rat:Intraperitoneal)	Causes skin and serious eye irritation. May cause respiratory irritation	CEPC	LC3, Beclin-1, P62, Parkin	50 μM (*In vitro*)	Activation of the SIRT3/FOXO3a/Parkin signaling axis enhances mitophagy and suppresses NLRP3 inflammasome activation, thereby attenuating chondrocyte apoptosis.	^[230]^
									50 mg/kg/qod (*In vivo*/Mice/Intraperitoneal injection)		
21	Sesamin	Lignans	Otanthus maritimus, Apis	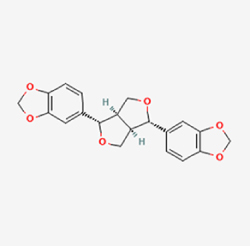	Unknown	May cause an allergic skin reaction	CEPC	LC3, P62	1 μM (*In vitro*)	Targeting BECN2 downregulates the expression of ATG14, VPS34, and GASP1, thereby inhibiting autophagosome formation. Reducing the expression of NLRP3, NLRC4, and AIM2 alleviates pan-inflammasome activation and inflammatory responses.	^[231]^
									80 mg/kg/d (*In vivo*/Rat/Intragastrically)		

Studies have demonstrated that, despite differences in their specific molecular mechanisms, compounds such as taurine, proanthocyanidins, and andrographolide improve mitochondrial homeostasis and reduce oxidative stress in NPCs by regulating mitophagy via the PINK1/Parkin pathway, thereby suppressing apoptosis. Similarly, naringin targets chondrocyte mitophagy in the CEPCs through the same mechanism to inhibit apoptosis^[209–211]^. Apigenin, a flavonoid abundantly present in fruits and vegetables from temperate and tropical regions, with particularly high concentrations in celery. Extensive research has demonstrated that apigenin possesses multiple pharmacological effects, including antitumor, cardiovascular protective, and antiviral activities^[212]^. It is inexpensive, readily available, and characterized by low toxicity and nonmutagenicity. In vitro studies indicate that apigenin protects NPCs against apoptosis, senescence, and ECM degradation by restoring autophagic flux^[213]^. Its glycoside form, Apigetrin, alleviates IVDD by targeting and regulating autophagy in NPCs, thereby improving inflammatory responses^[214]^. Quercetin, a natural flavonol, is widely found in the flowers, leaves, buds, seeds, and fruits of various plants. It has been shown to possess antioxidant and anti-inflammatory properties, demonstrating efficacy in degenerative diseases^[215]^. Research indicates that quercetin prevents ECM degradation and protects NPCs from apoptosis by modulating the p38MAPK signaling pathway. Its well-established preparation methods and low cost make it a promising anti-IVDD agent. However, its classification as a Group 3 carcinogen raises concerns regarding potential toxic side effects that warrant further investigation^[216]^. Resveratrol, the most frequently cited natural product in this study, is a phytoalexin produced by plants in response to stress and is found in grape skins and leaves. It is a bioactive component of wine and grape juice. Resveratrol has been shown to inhibit apoptosis in both NPCs and AFCs^[54,55]^ while also suppressing inflammatory responses in CEPCs^[53]^. Regarding autophagy, resveratrol activates the AMPK/SIRT1 signaling pathway, reducing TNF-α-induced MMP-3 expression in human NPCs. Additionally, it improves mitochondrial dysfunction by enhancing autophagy in NPCs^[217,218]^. Collectively, these findings indicate that resveratrol alleviates IVDD through multiple mechanisms, highlighting its significant therapeutic potential.

Due to space constraints, details on other effective natural compounds are provided in Table 6. To further explore the role of natural products in IVDD treatment and offer a comprehensive reference for new drug development, additional compounds with therapeutic potential are included in this study. Detailed information can be found in the supplementary materials (Supplementary Digital Content Table 3, available at: http://links.lww.com/JS9/F228 and Supplementary Digital Content Table 4, available at: http://links.lww.com/JS9/F228).

## Discussion

IVDD is one of the most prevalent degenerative disorders in clinical practice, driven by complex multilevel, multifactorial, and multitarget mechanisms. With the global population aging and IVDD increasingly affecting younger individuals, the associated workforce loss and economic burden have become major societal challenges. Given the limitations of current pharmacological and surgical treatments, there is an urgent need to identify novel, accessible, and effective compounds to lay the molecular foundation for new drug development and therapeutic strategies. PCD plays a central role in regulating cellular homeostasis and function. As a precisely controlled process, PCD maintains tissue equilibrium through intricate molecular networks. In the context of IVDD, the survival, activity, and functional state of IVD cells are critical, making targeted modulation of PCD a promising therapeutic strategy. Natural products, as inducers or inhibitors of PCD, have demonstrated significant therapeutic potential across various diseases, including IVDD. Nature provides a vast reservoir of bioactive compounds, and as our study illustrates, numerous natural products effectively modulate PCD through common signaling pathways, offering new therapeutic avenues for IVDD. These findings pave the way for future drug development and more effective treatment strategies.

A total of 134 compounds were analyzed, comprising 39 flavonoids, 32 terpenoids, 13 alkaloids, 9 phenolic acids, and a smaller number of additional compounds. The chemical structures of these natural products dictate both the shared mechanisms and distinct functional roles in regulating PCD. Predominant natural products, including flavonoids and terpenoids, confer protection against IVDD by indirectly mitigating oxidative stress and inflammation-induced PCD. This is achieved through scavenging ROS and suppressing inflammatory signaling pathways, such as NF-κB. Crucially, structural differences also underlie functional specialization among compound classes. Flavonoids exhibit diverse properties, with potent free radical scavenging and antioxidant activity representing their hallmark characteristics^[232]^. Characterized by an almost universal C6–C3–C6 core skeleton, their radical scavenging capacity is dictated by the number and position of hydroxyl (–OH) groups. Through resonance stabilization, flavonoids donate electrons or hydrogen atoms to free radicals, generating relatively stable flavonoid radicals^[233]^. This mechanism inhibits oxidative stress-induced cell death pathways such as apoptosis and ferroptosis. Terpenoids, amphiphilic compounds built from isoprene (C_5_H_8_) units, possess hydrophilicity conferred by hydroxyl and carboxyl groups, and hydrophobicity determined by their carbon skeleton. Increasing carbon chain length enhances hydrophobicity, enabling long chains to bind phospholipid fatty acid tails within the membrane, thereby reducing the lipid bilayer’s free energy^[234]^. Embedding this hydrophobic skeleton within the membrane lipid layer increases phospholipid spacing and disrupts membrane microdomains. Consequently, membrane fluidity and permeability decrease, impacting the function of embedded proteins (e.g., receptors, ion channels). These alterations contribute significantly to terpenoids’ role in death receptor-mediated apoptosis and inflammasome receptor-mediated pyroptosis. Alkaloids, nitrogen-containing organic compounds with diverse structures and potent biological activities, exhibit PCD-regulatory effects intrinsically linked to their molecular frameworks. Major classes based on skeleton include indole, quinoline, and isoquinoline alkaloids^[235]^. Indole alkaloids leverage the indole ring’s hydrophobicity to bind tubulin, arresting mitosis and triggering the caspase cascade to induce apoptosis. Isoquinoline alkaloids frequently feature quaternary ammonium salts that bind kinase active sites, inducing apoptosis via pathways like PI3K/Akt inhibition, making them common anticancer drug candidates. Intriguingly, isoquinoline compounds can exhibit concentration-dependent duality – inhibiting apoptosis at low levels and promoting it at high levels^[236,237]^. Their regulation of other PCD modalities primarily involves functional group modifications, an area warranting further investigation. Carboxyl groups directly attached to the benzene ring. The carboxyl groups chelate metal ions (e.g., Mn^2^⁺, Fe^3^⁺), disrupting the Fenton reaction through metal valence state transitions (e.g., Mn^2^⁺/Mn^3^⁺) to achieve multipathway ROS scavenging, thereby inhibiting ferroptosis^[238]^. This structural diversity allows distinct natural products to act synergistically on the multitarget pathological network underpinning IVDD. Future research should systematically investigate structural modifications and target interactions to optimize the therapeutic specificity of natural products for IVDD.

The founder of toxicology, Paracelsus, famously stated: “The dose makes the poison.” This principle underscores the importance of defining the safety margin, effective therapeutic dose, and the dose–response relationship – critical prerequisites in the development of any novel drug.In this study, we systematically evaluated the pharmacological toxicity of a series of natural products. Preliminary screening revealed that nearly all tested compounds exhibited acute irritant toxicity affecting the skin, eyes, and respiratory tract. These findings highlight the necessity of strict adherence to protective protocols during experimental procedures. In contrast, knowledge regarding the chronic toxicity of natural products remains limited, with a notable lack of systematic studies and validated data. To quantitatively assess drug safety, we determined the median lethal dose (LD_50_) for different classes of natural products. Our results show that flavonoids and terpenoids generally have wider safety margins than alkaloids. Most compounds exhibited LD_50_ values exceeding 1 g/kg in mice via intraperitoneal injection or oral administration, indicating favorable safety profiles and promising potential for clinical translation. However, it is important to note that not all flavonoids and terpenoids possess high safety profiles. Therefore, it is essential to carefully determine the safe dosage range for each individual compound. Additionally, we conducted a comprehensive analysis of the dose–response relationships of all tested natural products in cell-based models. Experimental results indicated that the optimal concentration range for significant pharmacological activity in vitro was consistently between 25 and 50 μM. Accordingly, we documented the optimal effective concentration for each compound in specific cellular models. Despite exhaustive analysis, we were unable to identify a consistent pattern or systematic correlation in optimal bioactive concentrations across structural classes such as flavonoids, terpenoids, and alkaloids. This suggests that the required concentrations are likely dependent on the unique molecular characteristics of individual compounds and their specific biological targets.

Despite the promising therapeutic potential of numerous natural products for IVDD, significant challenges and controversies remain due to technical limitations and gaps in theoretical research on the underlying mechanisms. While targeting PCD in IVD cells has been widely acknowledged as a viable therapeutic approach, the quantitative relationship between PCD levels and IVDD progression remains unexplored. As a result, the absence of an effective quantitative evaluation system continues to hinder the diagnosis, treatment, and assessment of IVDD based on PCD levels. Moreover, PCD is a dynamic process influenced by multiple factors, and its effects may vary under different conditions. For instance, autophagy exhibits a dual role in IVDD, where indiscriminate promotion or inhibition may inadvertently accelerate disc degeneration. Therefore, a deeper understanding of the molecular regulatory mechanisms of PCD and their precise correlation with IVDD pathogenesis is crucial for developing novel therapies targeting nucleus pulposus cells to prevent and treat IVDD.

The interplay between different forms of PCD is an area of growing research interest. Cellular pathophysiological processes are inherently interconnected. Apoptosis and necroptosis are mutually antagonistic processes. Caspase-8 plays a central regulatory role in both processes. In the TNF signaling pathway, activated caspase-8 cleaves RIPK1 and RIPK3, thereby suppressing necroptosis and promoting classical apoptosis. Conversely, when caspase-8 activity is inhibited, RIPK1 interacts with RIPK3 to phosphorylate MLKL, leading to membrane disruption, cellular rupture, and the release of DAMPs, ultimately triggering necroptosis^[239]^. Lv *et al*^[240]^ reported elevated expression of RIPK3 and MLKL in degenerated nucleus pulposus tissue, alongside apoptotic markers such as caspase-3. This suggests that cells exist in a transitional state characterized by “incomplete apoptosis and partial necrosis,” releasing a mixture of apoptotic bodies and DAMPs that amplify inflammatory cascades. The coexistence of these markers provides direct evidence of dysregulated pathway competition and identifies a critical therapeutic target in IVDD. The disrupted balance between PCD pathways drives a shift from regulated apoptosis to inflammatory necrosis, thereby accelerating disc degeneration. The interaction between apoptosis and pyroptosis amplifies inflammatory cascades. The key apoptotic effector caspase-3 cleaves GSDME at conserved aspartate residues, releasing its GSDME-N. GSDME-N oligomerizes on the plasma membrane, forming 10–20 nm pores that disrupt ion homeostasis and osmotic balance, thereby exacerbating pyroptosis and completing the switch from apoptosis to pyroptosis^[92,93]^. This transition triggers a pronounced inflammatory response within the intervertebral disc microenvironment, accelerates ECM degradation, and marks a critical shift from “compensation” to “decompensation” during IVDD^[241]^. In contrast to the relationship between necroptosis and pyroptosis, apoptosis and autophagy exhibit bidirectional antagonism. Protective autophagy inhibits apoptosis by clearing damaged mitochondria and reducing Cyt C release^[242]^, thereby suppressing apoptosome formation. However, under severe stress, autophagy may promote apoptosis by supplying ATP to support energy-demanding apoptotic processes Studies have shown that in IVDs, impaired chaperone-mediated autophagy leads to the accumulation of the pro-apoptotic factor PLCG1. Disruption of calcium homeostasis subsequently induces apoptosis in NPCs, thereby accelerating IVDD^[243]^. Apoptosis and ferroptosis also exhibit complex cross-regulation. The tumor suppressor p53 promotes apoptosis via BAX/BAK activation and bidirectionally regulates ferroptosis. Under conditions of elevated ROS or GPX4 inhibition, p53 promotes ferroptosis through the canonical pathway by modulating iron metabolism and ferredoxin reductase expression. Alternatively, p53 can enhance ferroptosis through the noncanonical pathway by downregulating ALOX12 and activating SLC7A11^[244]^. Conversely, p53 may confer resistance to ferroptosis by inducing p21, which promotes GSH synthesis, enhances GPX4 activity, and detoxifies lipid peroxides and ROS^[245]^. Therefore, elucidating p53 expression dynamics and its regulatory role in apoptosis–ferroptosis interplay is critical.

The synergy between necroptosis and pyroptosis resembles that between apoptosis and pyroptosis. RIPK3, a necroptosis executor, phosphorylates MLKL, promoting its oligomerization and translocation to plasma or lysosomal membranes, where it forms transmembrane pores. This results in ion efflux (e.g., K⁺, Na⁺, Ca^2^⁺), a sharp reduction in intracellular K⁺, activation of the NLRP3 inflammasome, and initiation of a strong inflammatory response that further amplifies pyroptosis. Autophagy plays a dual role in necroptosis: it inhibits necroptosis by degrading key mediators such as RIPK1, RIPK3, and TRIF, but excessive mitophagy or ER-phagy may promote necroptosis via increased ROS production and death signaling. Necroptosis and ferroptosis also share common drivers and regulatory mechanisms. Iron overload is a key contributor to both forms of cell death, while ROS generated during ferroptosis can activate RIPK1, and DAMPs such as HMGB1 released during necroptosis can exacerbate oxidative stress, forming a positive feedback loop.

The relationship between pyroptosis and autophagy is both antagonistic and synergistic. Autophagy alleviates pyroptosis by eliminating intracellular pathogens, while excessive autophagy can cause lysosomal rupture and the release of cathepsins, which activate the NLRP3 inflammasome and promote pyroptosis^[246]^. Similarly, ferroptosis and pyroptosis share regulatory links – lipid peroxidation byproducts from ferroptosis activate NLRP3, promote IL-1β secretion, and amplify inflammation^[247]^. Autophagy also contributes to ferroptosis by mediating ferritin degradation via NCOA4, increasing free iron levels and enhancing lipid peroxidation. Conversely, knockout of the autophagy-related gene ATG7 blocks GPX4 degradation and confers resistance to ferroptosis^[248]^.

The interplay among various PCD pathways has become a major focus of current biomedical research. Studies have shown that the apoptotic executioner caspase-8 inhibits necroptosis by directly cleaving RIPK1, CYLD, and the necroptotic effector kinase RIPK3. Conversely, RIPK3 can activate the NLRP3 inflammasome, thereby promoting caspase-1-dependent pyroptosis, establishing a functional axis that interlinks apoptosis, necroptosis, and pyroptosis^[249]^. Enhancing mitophagy, reducing oxidative stress, and inhibiting ferroptosis in chondrocytes can alleviate ECM degradation and apoptosis in cartilage endplate cells, thereby delaying the progression of IVDD^[182]^. The complex crosstalk among PCD pathways forms ternary or multiplex regulatory networks that are essential for maintaining tissue and organismal homeostasis.

Overall, PCD pathways form a dynamic regulatory network through shared molecular mediators (e.g., caspase family proteins, RIPK1, and Bcl-2 proteins), metabolic processes (e.g., iron homeostasis and lipid peroxidation), and inflammatory signaling cascades, and recent studies increasingly focus on the molecular mechanisms and therapeutic interventions that influence multiple PCD pathways rather than a single mode of cell death. This comprehensive approach allows for a more holistic evaluation of drug efficacy and the identification of optimal mechanisms of action.

In recent years, the advancement of cutting-edge technologies such as gene editing and single-cell omics has ushered research on IVDD and PCD into a new era of precise regulation and high-resolution analysis. The CRISPR-Cas system, as a powerful gene-editing tool, has been extensively employed for the functional validation and targeted modulation of key PCD-related genes, laying a theoretical foundation for developing targeted therapies for IVDD. CRISPR-Cas9-mediated knockout of pro-apoptotic or inflammasome-related genes has been shown to effectively reduce NPC death, thereby mitigating disc degeneration^[250]^. Moreover, the combination of CRISPR with nano-delivery systems (e.g., liposomes, AAV vectors) holds promise for achieving precise *in vivo* regulation of PCD pathways, offering new opportunities for IVDD treatment^[251]^. Simultaneously, single-cell omics technologies, particularly scRNA-seq, have revealed the substantial heterogeneity of intervertebral disc cells and identified specific subpopulations that are more susceptible to PCD during degeneration^[252]^. scRNA-seq enables high-resolution tracking of PCD dynamics across diverse disc cell types and provides critical insights into cell subsets characterized by stemness, inflammation, or degeneration-prone properties^[253]^. Further integration with single-cell ATAC-seq and spatial transcriptomics^[254]^ will facilitate a comprehensive understanding of chromatin accessibility changes and spatial localization of PCD-regulatory elements, enabling detailed mapping of PCD regulatory networks within disc tissues. Looking ahead, the synergistic application of CRISPR-based gene editing, single-cell omics, and natural product-derived therapeutic strategies is expected to enable precise and individualized intervention in PCD, thereby opening new avenues for personalized treatment of IVDD.

While natural products hold significant promise for treating IVDD, their clinical application remains challenging. These compounds often exhibit inherent limitations, including low oral bioavailability, rapid metabolism, and poor tissue-specific distribution. Additionally, their narrow therapeutic windows and potential toxicity further constrain their use. To address these challenges, researchers are exploring innovative drug delivery systems to optimize their therapeutic efficacy. Given current advancements, it is crucial to identify natural modulators of PCD – or even synergistic regulators that balance multiple PCD pathways – tailored to different stages of IVDD. Moreover, further investigation into their pharmacokinetics, metabolism, and pharmacodynamic properties is essential. Strategies such as nano-drug delivery systems and structural modifications should be employed to enhance bioavailability and improve therapeutic outcomes.

## Summary

In conclusion, numerous natural products have exhibited significant therapeutic potential for IVDD by modulating PCD pathways. These bioactive compounds can precisely regulate various cell death mechanisms in disc cells, including apoptosis, necroptosis, pyroptosis, ferroptosis, and autophagy. Their multitarget properties enable the synergistic regulation of multiple PCD pathways, resulting in unique pharmacological effects. However, several challenges remain in this field, including elucidating the molecular mechanisms underlying different PCD types in IVDD, establishing screening systems for natural products based on PCD regulation, optimizing drug delivery to enhance bioavailability, minimizing potential toxicity, and facilitating clinical translation. Future research should prioritize investigating the regulatory mechanisms governing PCD crosstalk, while advancing precision therapy through structural modifications and nano-drug delivery technologies. These advancements will provide novel therapeutic strategies and methodological foundations for the clinical management of IVDD.

## Supplementary Material

## Data Availability

Data sharing is not applicable to this article as no new data were generated or analyzed in this study. This is a review based on previously published literature.

## Research registration unique identifying number (UIN)

This article is a review of previously published studies and does not involve prospective enrollment of human participants. Therefore, registration in a public trials registry is not required, and a Unique Identifier Number (UIN) is not applicable.
