# Supplementary material for: Natural products targeting programmed cell death: a novel therapeutic strategy for intervertebral disc degeneration
Source: Int J Surg. 2025 Oct 7;112(1):1601–34. doi: 10.1097/JS9.0000000000003380 (PMC12825861; doi:10.1097/JS9.0000000000003380)
Supplement: Supplementary file 1 [file js9-112-1601-001.docx]

Table 1 Natural Products Targeting Apoptosis for IVDD

|  | Natural products | Chemical Class | Sources | Structure | Target cell types | Apoptosis-related mode of action | Key Regulatory Mechanisms | References |
| --- | --- | --- | --- | --- | --- | --- | --- | --- |
| 1 | Ginsenoside Rg3 | Terpenoids | Panax ginseng, Panax japonicus | 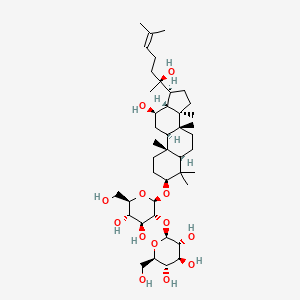 | NPC | BAX, Bcl-2, | By inhibiting the phosphorylation level of AMPK, it ameliorates IL-1β-induced cell apoptosis and alleviates IVDD | [1] |
| 2 | Ginsenoside Rg1 | Terpenoids | Panax ginseng, Panax japonicus | 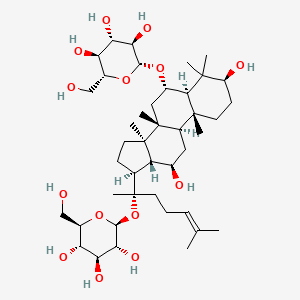 | NPC | BAX，Bcl-2 | Inhibition of NF-κB signaling pathway activation suppresses IL-1β-induced NP cell apoptosis, inflammatory response, and ECM degradation | [2] |
| 3 | Kaempferol | Flavonoids | Hydrangea serrata, Caragana frutex | 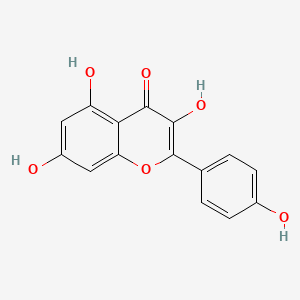 | NPC | BAX | By inhibiting the phosphorylation level of AMPK, it ameliorates IL-1β and other inflammatory factors-induced cell apoptosis | [3] |
| 4 | Phillyrin | Phenolic acids | Bupleurum wenchuanense, Osmanthus fragrans | 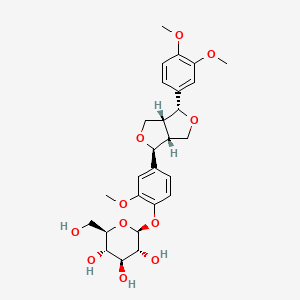 | NPC | BAX, Bcl-2，Caspase-3 | Attenuating the activation of the NF-κB inflammatory pathway and ROS production significantly inhibits IL-1β-mediated ECM degradation and reduces apoptosis. | [4] |
| 5 | Isoliquiritigenin | Flavonoids | Glycyrrhiza, pallidiflora, Morus cathayana | 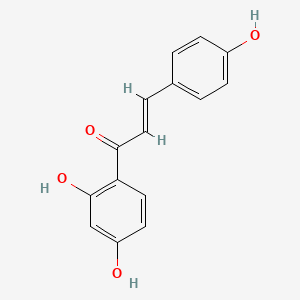 | NPC | BAX, Bcl-2, caspase-3, caspase-9, | Through a PPARγ-dependent mechanism, it balances ECM synthesis and degradation by inhibiting oxidative stress, restoring mitochondrial function, and reducing apoptosis in nasopharyngeal carcinoma cells | [5] |
| 6 | Alpinetin | Flavonoids | Katsumadai seed, Boesenbergia rotunda, Alpinia pinnanensis | 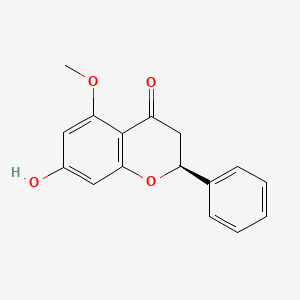 | NPC | BAX, Bcl-2, caspase-3 | Alleviating IL-1β-induced inflammatory response and oxidative stress enhances Aggrecan and Collagen II expression, reduces MMP-3 levels, and ameliorates ECM degradation. Additionally, it inhibits IL-1β-induced activation of the TLR4/MyD88 pathway and apoptosis of NPCs. | [6] |
| 7 | Acetylshikonin | Quinones | Shikonin, Echium italicum, Onosma paniculata | 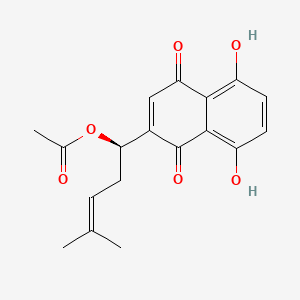 | NPC | BAX, Bcl-2, caspase-3 | Inhibiting SOX4 expression through the PI3K/Akt pathway delays IVDD. | [7] |
| 8 | Fucoxanthin | Terpenoids | Jania, Corbicula sandai | 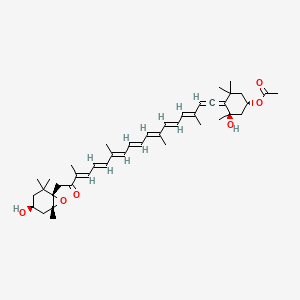 | NPC | PERK, eIF2α, ATF4, CHOP | By upregulating SIRT1, it inhibits the activation of the PERK-eIF2α-ATF4-CHOP pathway, reduces ECM degradation, and suppresses apoptosis induced by ERS. | [8] |
| 9 | puerarin | Flavonoids | Bupleurum chinense, Pueraria calycina | 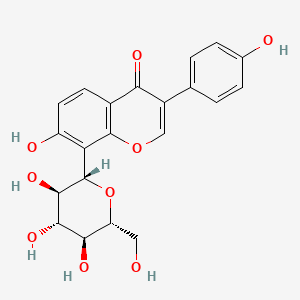 | NPC | Bcl-2, caspase-3, caspase-8, | By inhibiting LPS-induced expression of IL-1β, TNF-α, and Caspase-3, NPCs apoptosis is alleviated. | [9] |
| 10 | Quercetin | Flavonoids | Tea, tomatoes, cherries, grapes, apples,onions | 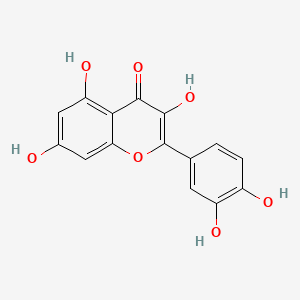 | NPMSC | / | The stabilization of HIF1A mitigated compression-induced oxidative stress and apoptosis in NPMSCs. | [10] |
| 11 | Curculigoside | Phenolic Glycosides | Curculigo sinensis, Degeneria vitiensis | 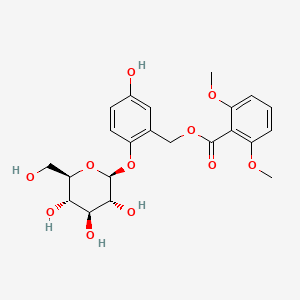 | NPC | BAX, Bcl-2, caspase-3 | By inhibiting STAT3 phosphorylation to upregulate BMAL1 expression, thereby alleviating NPCs apoptosis and enhancing ECM component synthesis | [11] |
| 12 | Aucubin | Terpenoids | Veronica kellereri, Plantago uniflora | 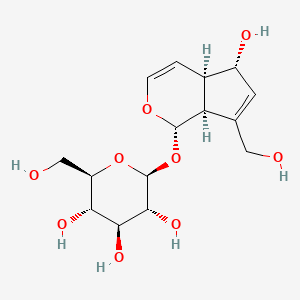 | NPC | BAX, Bcl-2, caspase-3 | By activating the PI3K/mTOR pathway, NPCs apoptosis and ECM degradation are inhibited. | [12] |
| 13 | Andrographolide | Terpenoids | Andrographis paniculata, Cymbopogon schoenanthus | 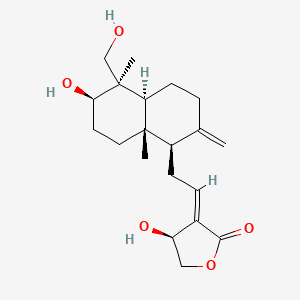 | NPC | / | Activation of the MAPK/Nrf2/HO-1 signaling pathway ameliorates IVDD by suppressing static mechanical stress-induced ROS accumulation in NPCs. | [13] |
| 14 | Ginkgetin | Flavonoids | Selaginella sinensis, Taxus cuspidata | 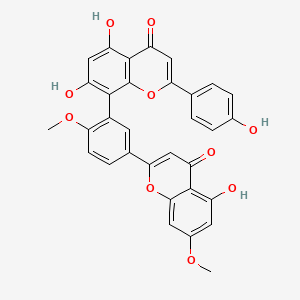 | NPC | BAX, Bcl-2, caspase-3 | Suppression of NLRP3 inflammasome-associated proteins rescues IL-1β-induced dysregulation in NPCs, including aberrant proliferation, apoptosis, inflammatory activation, and ECM catabolism. | [14] |
| 15 | Psoralen | Coumarins | Ficus erecta var. beecheyana、Hoita macrostachya | 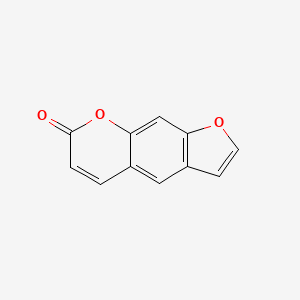 | NPC | Bcl-2、BAX | Coordinated upregulation of aggrecan, COL2A1, Bcl-2 and CDK2 with concomitant downregulation of Bax, p16, p21 and inflammatory cytokines effectively counteracts cellular senescence, cell cycle arrest and apoptotic pathways. | [15] |
| 16 | Procyanidin B2 | Flavonoids | Crataegus monogyna, Vitis vinifera, Litchi chinensis, apple, Ecdysanthera, red wine. | 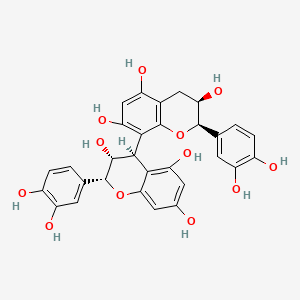 | NPC | caspase-3 | Activation of the PI3K/Akt signaling pathway mitigates oxidative stress-induced apoptosis in rat NPCs through Nrf2 upregulation. | [16] |
| 17 | Emodin | Quinones | Rhubarb, buckthorn | 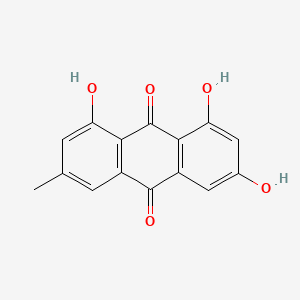 | NPC | BAX, Bcl-2, caspase-3 | Suppression of ROS-mediated NF-κB activation confers cytoprotection in NPCs by attenuating IL-1β-induced apoptotic cascades and pro-inflammatory signaling. | [17] |
| 18 | Sulforaphane | Isothiocyanates | Broccoli, cabbage | 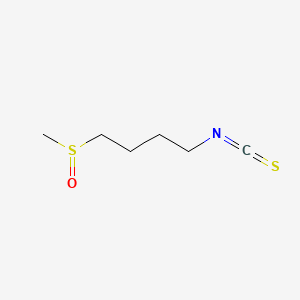 | NPC | GRP78, CHOP, PERK, Eif2α, caspase-3, caspase-12 | Activation of the Nrf2/HO-1 signaling axis ameliorates ERS and suppresses apoptosis by scavenging intracellular ROS accumulation. | [18] |
| 19 | Baicalein | Flavonoids | Scutellaria baicalensis | 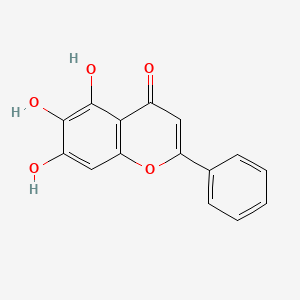 | NPC | BAX, Bcl-2, caspase-3 | Enhancement of the PI3K/Akt signaling axis confers cytoprotection in NPCs by counteracting TNF-α-triggered apoptotic pathways. | [19] |
| 20 | Fisetin | Flavonoids | Strawberries, apples, persimmons, cucumbers, Onions | 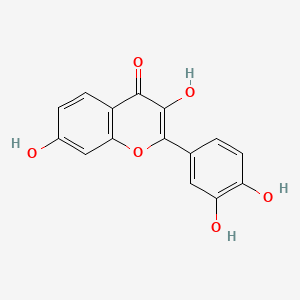 | NPMSC | / | SIRT1-mediated attenuation of H_2_O_2_-induced pathological cascades preserves NPMSCs viability through coordinated suppression of apoptosis, inflammasome activation, and ECM catabolism. | [20] |
| 21 | Sinomenine | Alkaloids | Stephania cephalantha, Sinomenium acutum | 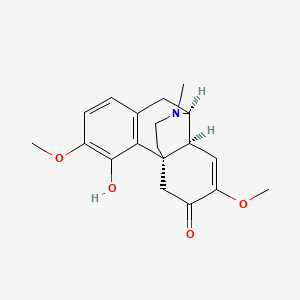 | NPC | / | Coordinated activation of the Keap1/Nrf2 antioxidant axis coupled with suppression of NF-κB inflammatory signaling effectively antagonizes IL-1β-triggered apoptotic execution in PCs. | [21] |
| 22 | Emodin | Quinones | Rhubarb, buckthorn | 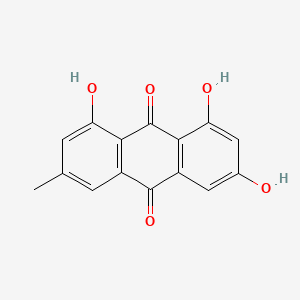 | NPC | Bcl-2, caspase-3 | LRP1-dependent inhibition of NF-κB signaling axis effectively mitigates ECM catabolism and counteracts apoptotic machinery activation. | [22] |
| 23 | Kempferol | Flavonoids | Hydrangea serrata, Caragana frutex | 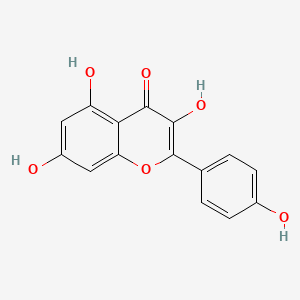 | NPC | BAX, Bcl-2, caspase-3 | Attenuation of IL-1β-driven phosphorylation potentiation in p38, JNK, and ERK1/2 signaling cascades modulates extracellular matrix homeostasis through downregulation of catabolic mediators (MMP3/ADAMTS-4) and upregulation of anabolic components (aggrecan/COL2A1), while enhancing cellular viability via dual suppression of senescence-associated secretory phenotype (SASP) and intrinsic apoptotic pathway activation. | [23] |
| 24 | Astragaloside IV | Terpenoids | Astragalus hoantchy, Astragalus lepsensis | 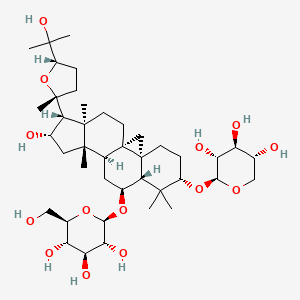 | NPC | BAX, Bcl-2, caspase-3 | Activation of the PI3K/Akt signaling axis counteracts IL-1β-induced pathological cascades in NPCs, effectively suppressing apoptotic progression, inflammatory activation, and ECM catabolism. | [24] |
| 25 | Baicalin | Flavonoids | Scutellaria prostrata, Scutellaria scandens | 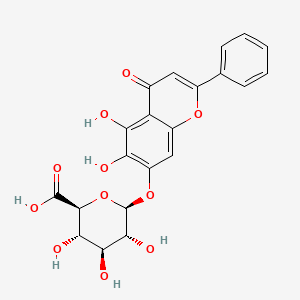 | NPC | Bcl-2, caspase-3 | Counteracting IL-1β-triggered ROS generation while restoring COL2A1 and aggrecan expression levels. | [25] |
| 26 | Polyphyllin I | Saponins | Paris polyphylla var. chinensis, Polygonatum kingianum | 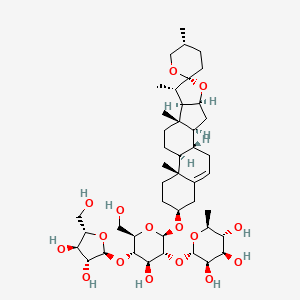 | NPC | BAX, Bcl-2, caspase-3 | Targeting the miR-503-5p/Bcl-2 regulatory axis effectively counteracts IL-1β-driven apoptotic signaling in NPCs of the intervertebral disc. | [26] |
| 27 | Aucubin | Terpenoids | Veronica kellereri, Plantago uniflora | 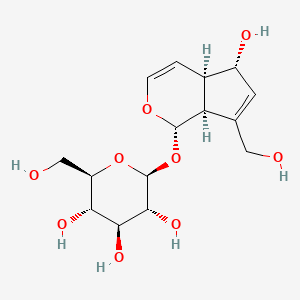 | NPC | / | Targeted suppression of TLR4/NF-κB signaling activation mitigates inflammatory responses and reduces apoptosis and senescence in NPCs. | [27] |
| 28 | Esculetin | Coumarins | [Caragana frutex](https://pubchem.ncbi.nlm.nih.gov/taxonomy/47643#section=Natural-Products)、[Sonchus fruticosus](https://pubchem.ncbi.nlm.nih.gov/taxonomy/50200#section=Natural-Products) | 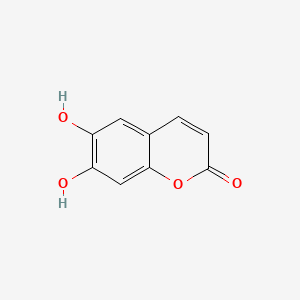 | NPC | caspase-3 | Modulation of the Nrf2/HO-1/NF-κB signaling network orchestrates cytoprotective responses to IL-1β-driven functional impairments in NPCs, mitigating oxidative damage, inflammatory activation, and matrix homeostatic disruption. | [28] |
| 29 | Ginsenoside Rg1 | Terpenoids | Panax ginseng, Panax japonicus | 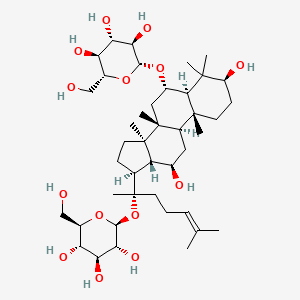 | NPC | / | Suppression of YAP1/TAZ signaling axis activation attenuates the progression of IVDD hrough mechanotransduction modulation and ECM homeostasis preservation. | [29] |
| 30 | Taurine | Amino Acids | marine fish， shellfish | 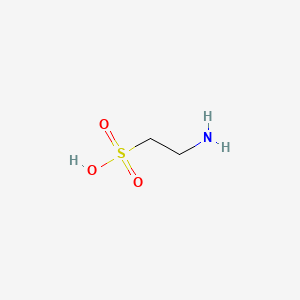 | NPC | GRP78, CHOP, caspase-12 | Suppression of ERS orchestrates cellular proteostasis in NPCs, effectively attenuating apoptotic cascades and ECM catabolic processes. | [30] |
| 31 | Arctigenin | Lignans | Saussurea parviflora、Saussurea salicifolia | 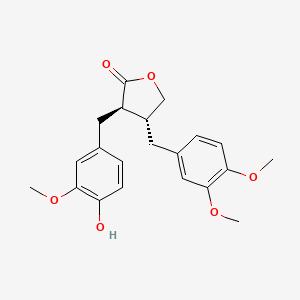 | NPC | BAX, Bcl-2, caspase-3 | Upregulation of miR-483-3p-mediated NF-κB pathway activation orchestrates cytoprotection in HNPCs, counteracting apoptotic signaling, ECM catabolism, and pro-inflammatory cytokine cascades. | [31] |
| 32 | Astragaloside IV | Terpenoids | Astragalus hoantchy, Astragalus lepsensis | 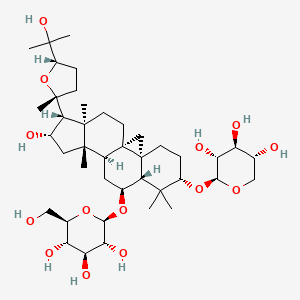 | NPC | BAX, Bcl-2, caspase-3 | Suppression of IL-1β-induced cytoplasmic NF-κB phosphorylation through IKKβ blockade alleviates the pathological triad of inflammation, apoptosis, and ECM catabolism in NPCs. | [32] |
| 33 | Evodiamine | Alkaloids | Tetradium ruticarpum、Spiranthera odoratissima | 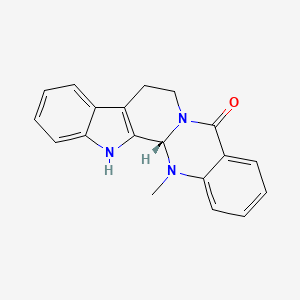 | NPC | BAX, Bcl-2 | Activation of the PI3K/Akt signaling axis upregulates SIRT1 expression, orchestrating a cytoprotective mechanism that counteracts LPS-induced apoptosis, ECM catabolism, and inflammatory activation in NPCs. | [33] |
| 34 | Ganoderic Acid A | Terpenoids | Ganoderma sinense, Ganoderma lucidum, and Wolfiporia cocos | 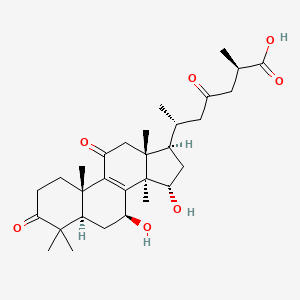 | NPC | BAX, Bcl-2, caspase-3 | Suppression of the TLR4/NLRP3 signaling axis mitigates H₂O₂-induced apoptotic cell death, oxidative stress, and inflammatory responses through coordinated blockade of inflammasome activation and redox imbalance. | [34] |
| 35 | Naringin | Flavonoids | Salvia officinalis, Citrus reticulata | 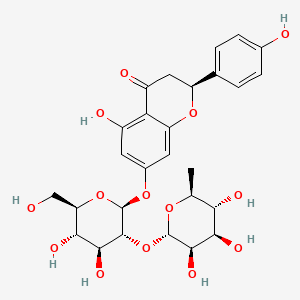 | AFC | Cyt C, | Suppression of NF-κB signaling activation attenuates oxidative stress and restores mitochondrial homeostasis, thereby counteracting cyclic tensile loading-induced apoptosis in AFCs via coordinated modulation of redox imbalance and bioenergetic recovery. | [35] |
| 36 | α-Mangostin | Xanthones | Garcinia cowa，Garcinia merguensis，Garcinia mangostana | 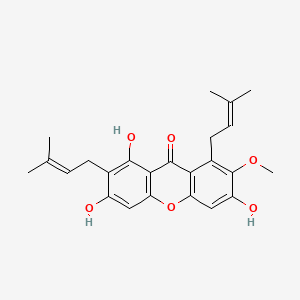 | NPC | BAX, Bcl-2, caspase-3 | Modulation of the NF-κB signaling pathway suppresses NLRP3 inflammasome-mediated apoptosis in LPS-induced NPCs. | [36] |
| 37 | Cyanidin | Flavonoids | Camellia sinensis, Viburnum rafinesquianum | 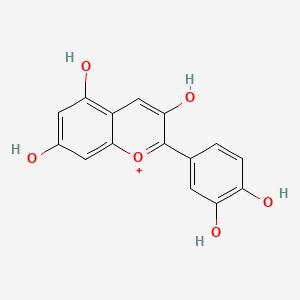 | NPC | BAX, Bcl-2, caspase-3 | Modulation of the JAK2/STAT3 signaling axis orchestrates functional restoration of NPCs in IVDD through coordinated suppression of inflammatory cascades and ECM homeostatic reprogramming. | [37] |
| 38 | Hyperoside | Flavonoids | Camellia sinensis, Geranium carolinianum | 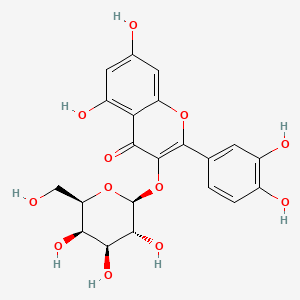 | NPC | BAX, Bcl-2, GPR78, PERK, CHOP, caspase-12 | The amelioration of TNF-α-induced inflammation, ECM degradation, and endoplasmic reticulum stress-mediated apoptosis via the SIRT1/NF-κB and Nrf2 signaling pathways. | [38] |
| 39 | Kukoamine A | Polyamines | Lycium chinense | 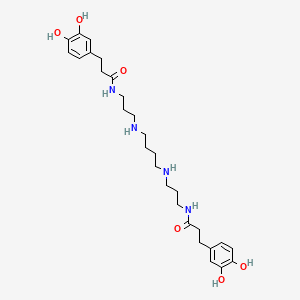 | NPC | BAX, Bcl-2, caspase-3 | Activation of the PI3K/Akt pathway alleviates LPS-induced apoptosis, ECM degradation, and inflammation in NPCs. | [39] |
| 40 | Proanthocyanidins | Flavonoids | Lathyrus laxiflorus, Vitis amurensis | 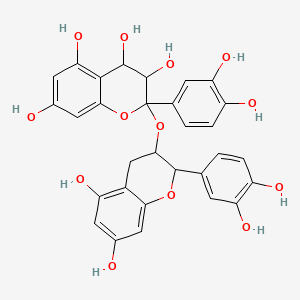 | NPC | BAX, Bcl-2, caspase-3, caspase-9 | Activation of the PI3K/Akt pathway inhibits IL-1β-induced apoptosis and senescence in NPCs. | [40] |
| 41 | Scutellarin | Flavonoids | Perilla frutescens, Scutellaria indica | 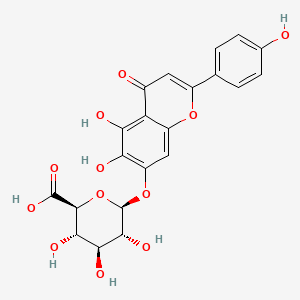 | NPC | BAX, Bcl-2, caspase-3 | Dual suppression of NF-κB and MAPK signaling pathways attenuates TNF-α-mediated NLRP3 inflammasome activation, preventing NPCs death. | [41] |
| 42 | Luteolin | Flavonoids | Camellia sinensis, Codonopsis lanceolata | 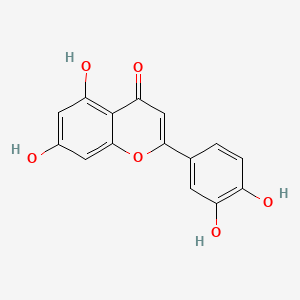 | NPC | BAX, Bcl-2, caspase-3 | argeting the Sirt6/NF-κB signaling axis suppresses TNF-α-induced inflammatory damage and senescence in HNPCs. | [42] |
| 43 | Hydroxysafflor yellow A | Flavonoids | Carthamus tinctorius | 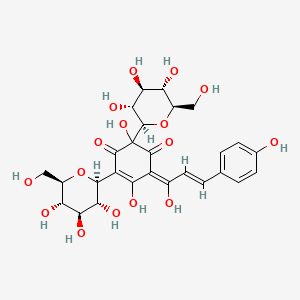 | NPC | BAX, Bcl-2, caspase-3, caspase-9 | Attenuation of TBHP-induced oxidative stress damage and apoptosis in NPCs lines restores ECM homeostasis. | [43] |
| 44 | Aloin | Quinones | Aloe ferox, Aloe africana | 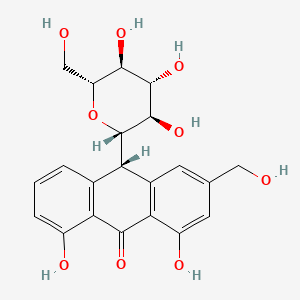 | NPC | BAX, Bcl-2, caspase-3 | Targeting the TAK1/NF-κB/NLRP3 signaling axis reverses TNF-α-induced dysregulated matrix metabolism and NPC apoptosis. | [44] |
| 45 | Cyanidin-3-glucoside | Flavonoids | Saccharomyces cerevisiae | 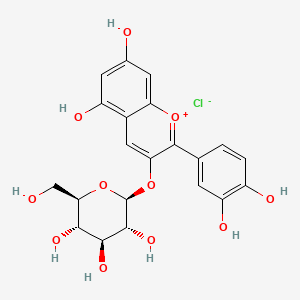 | NPC | BAX, Bim, caspase-3 | Activation of the Nrf2/HO-1 signaling axis orchestrates cytoprotective mechanisms against ROS-mediated cellular damage in HNPCs. | [45] |
| 46 | Panax notoginseng saponin | Terpenoids | Panax ginseng, Panax japonicus | / | NPC | BAX, Bcl-2, caspase-3 | Suppression of miR-222-3p expression enhances IL-1β-challenged proliferation of HNPCs while attenuating apoptosis, inflammatory activation, and ERS responses. | [46] |
| 47 | resveratrol | Stilbenes | Polygonum cuspidatum | 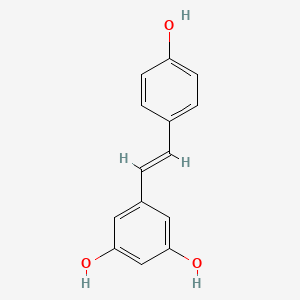 | CEPC | BAX, Bcl-2 | Modulation of TNF-α release and augmentation of IL-10 production significantly attenuate apoptosis in CEPCs through coordinated immunoregulatory mechanisms. | [47] |
| 48 | Higenamine | Alkaloids | [Gnetum montanum](https://pubchem.ncbi.nlm.nih.gov/taxonomy/3381#section=Natural-Products), [Gnetum parvifolium](https://pubchem.ncbi.nlm.nih.gov/taxonomy/33153#section=Natural-Products) | 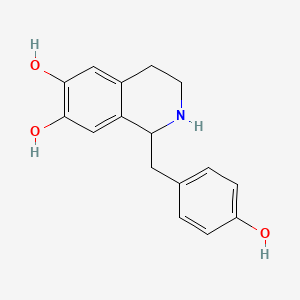 | NPC | BAX, Bcl-2, caspase-3 | Modulation of the ROS-mediated PI3K/Akt pathway attenuates IL-1β-induced apoptosis in HNPCs. | [48] |
| 49 | Salvianolic Acid B | Phenolic Acids | Salvia miltiorrhiza, Celastrus hindsii | 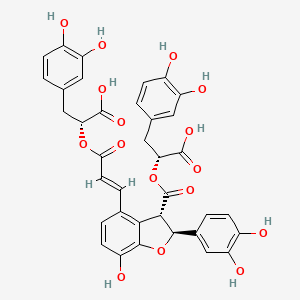 | NPC | BAX, Bcl-2, caspase-3 | Activation of the JAK2/STAT3 signaling axis enhances cellular proliferation and attenuates apoptotic signaling. | [49] |
| 50 | Mangiferin | Xanthones | Rigidella, Polygala tenuifolia | 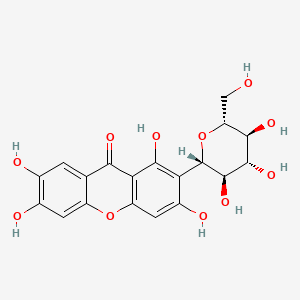 | NPC | BAX, Bcl-2, caspase-3 | Mitochondrial ROS scavenging in NPCs coupled with NF-κB signaling pathway inhibition suppresses apoptotic activation through dual modulation of oxidative stress and inflammatory signaling. | [50] |
| 51 | Tanshinone IIA | Terpenoids | Salvia miltiorrhiza, Salvia glutinosa | 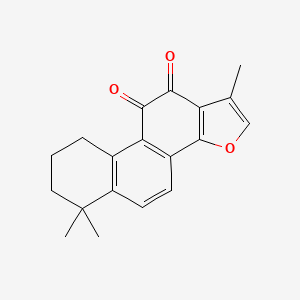 | NPC | caspase-1, caspase-3 | Suppression of the miR-223/JAK2/STAT1 signaling axis in NPCs attenuates inflammatory responses and mitigates apoptotic signaling. | [51] |
| 52 | Epigallocatechin-gallate | Flavonoids | Camellia sinensis, Eschweilera coriacea | 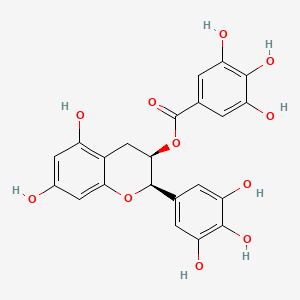 | NPC | BAX, Bcl-2, caspase-3 | Attenuation of H_2_O_2_-induced ECM degradation and suppression of apoptosis in NPCs mitigate oxidative damage through redox homeostasis modulation. | [52] |
| 53 | Cycloastragenol | Terpenoids | Astragalus microcephalus, Astragalus coluteocarpus | 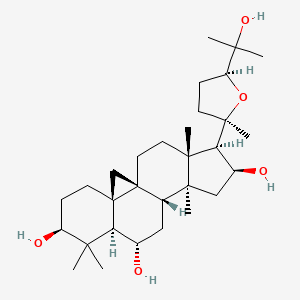 | NPC | BAX, Bcl-2, | Enhancement of telomerase activation and telomere elongation ameliorates high-glucose stress-induced senescence and apoptosis. | [53] |
| 54 | Resveratrol | Stilbenes | Polygonum cuspidatum | 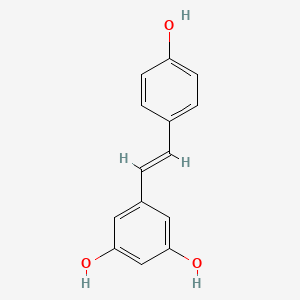 | NPC | caspase-3 | Coordinated suppression of the PI3K/AKT/mTOR and PI3K/AKT/GSK-3β signaling axes mitigates IL-1β-induced apoptotic cascades in NPCs through dual blockade of mitochondrial permeability transition and pro-apoptotic transcriptional programs. | [54] |
| 55 | Bergenin | Coumarins | Mallotus repandus, Peltophorum africanum | 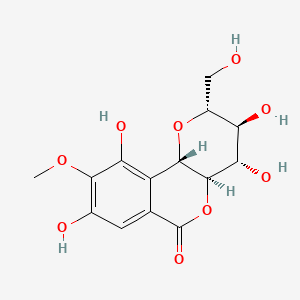 | NPC | caspase-3 | Dual modulation of PPAR-γ activation and NF-κB signaling pathway suppression mitigates H₂O₂-induced oxidative stress and apoptotic responses in HNPCs. | [55] |
| 56 | Shikonin | Quinones | Arnebia decumbens, Arnebia euchroma | 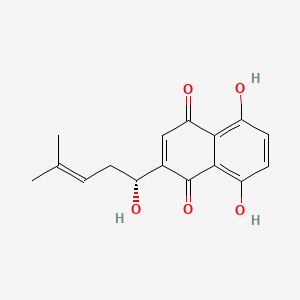 | NPC | BAX, Bcl-2, caspase-3 | Targeted suppression of the NF-κB signaling pathway mitigates inflammatory responses and counteracts apoptotic cascades in human primary NPCs. | [56] |
| 57 | Resveratrol | Stilbenes | Polygonum cuspidatum | 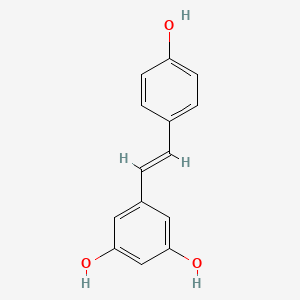 | AFC | BAX, Bcl-2, caspase-3 | Targeted attenuation of oxidative stress response in vitro counteracts TNF-α-mediated apoptotic signaling in AFCs. | [57] |
| 58 | Chlorogenic Acid | Phenolic Acids | Camellia sinensis, Meum athamanticum | 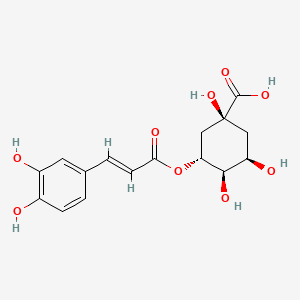 | CEPC | / | Suppression of NF-κB signaling axis in the cartilaginous endplate milieu confers multifaceted cytoprotection by counteracting endplate chondrocyte apoptosis and attenuating dedifferentiation-associated phenotypic regression. | [58] |
| 59 | Oxymatrine | Alkaloids | Sophora macrocarpa, Sophora chrysophylla | 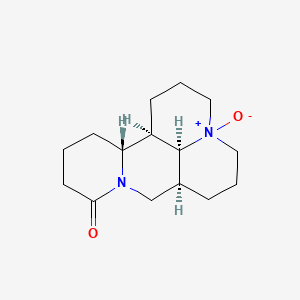 | NPC | / | Dual suppression of matrix metalloproteinase (MMP)-3/9 and interleukin (IL)-6 expression preserves type II collagen integrity and attenuates NPCs apoptosis. | [59] |
| 60 | Allicin | Organosulfur Compounds | Allium ursinum, Allium ampeloprasum | 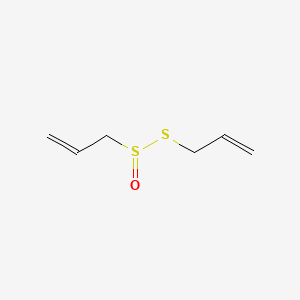 | NPC | BAX, Bcl-2, caspase-3, caspase-9 | Targeted suppression of the p38-MAPK signaling axis confers cytoprotection in NPCs by counteracting AOPP-mediated oxidative stress and rescuing mitochondrial bioenergetic dysfunction. | [60] |
| 61 | Ginsenoside Rg1 | Terpenoids | Panax ginseng, Panax japonicus | 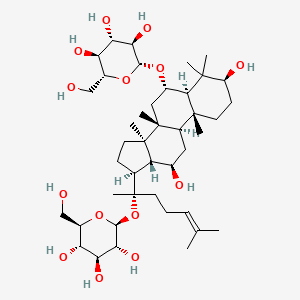 | NPC | / | Targeted suppression of the Wnt/β-catenin signaling axis confers dual therapeutic efficacy in degenerative NPCs by orchestrating ECM anabolic reprogramming and counteracting apoptotic cascades. | [61] |
| 62 | syringic acid | Phenolic Acids | Paeonia obovata, Rhinacanthus nasutus | 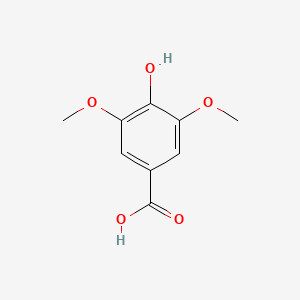 | NPC | Bcl-2, caspase-3 | Coordinated modulation of CACNA2D1 and PLK4 expression dynamics confers cytoprotection by reversing lipopolysaccharide (LPS)-induced cytotoxic cascades and restoring physiological expression patterns of Bcl-2 and caspase-3 apoptotic regulators. | [62] |
| 63 | Icariin | Flavonoids | Epimedium brevicornu, Epimedium truncatum | 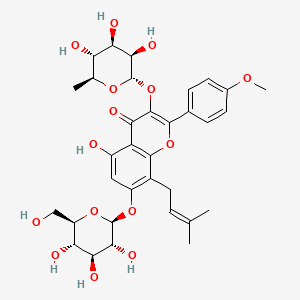 | NPC | BAX, Bcl-2, caspase-3 | Pharmacological inhibition of the PI3K/Akt-mediated Nrf2 signaling axis effectively mitigates H₂O₂-induced mitochondrial-dependent apoptotic cascades through coordinated suppression of oxidative stress executors and restoration of redox homeostasis. | [63] |
| 64 | Quercetin | Flavonoids | Tea, tomatoes, cherries, grapes, apples,onions | 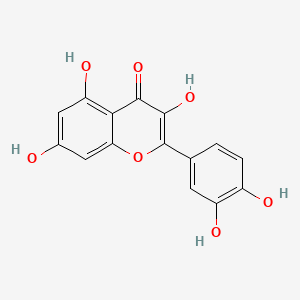 | NPC | / | SIRT1-autophagy axis activation orchestrates dual cytoprotection in NPCs through coordinated suppression of apoptotic machinery and preservation of ECM homeostasis. | [64] |
| 65 | Anisodamine | Alkaloids | Duboisia myoporoides, Hyoscyamus albus | 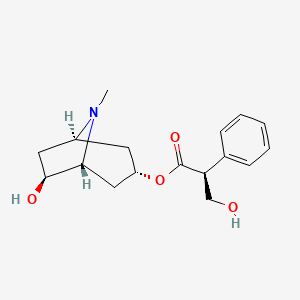 | NPC | BAX, Bcl-2 | IL-6/JAK/STAT3 axis inhibition mitigates NPC senescence and ECM degradation. | [65] |
| 66 | Grape seed extract | / | / | / | NPC | BAX | Prevent and repair punctured intervertebral discs, and prevent the degradation of collagen fibrils within disc tissues. | [66] |
| 67 | Epigallocatechin-gallate | Flavonoids | Camellia sinensis, Eschweilera coriacea | 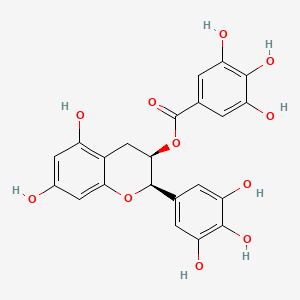 | NPC | BAX, Bcl-2, caspase-3, caspase-9 | Downregulation of the cGAS/Sting/NLRP3 pathway in H_2_O_2_-induced human nucleus pulposus cells (NPCs) exerts anti-apoptotic, anti-inflammatory, and cell viability-promoting effects. | [67] |
| 68 | Dioscin | Saponins | Dioscorea collettii, Dioscorea deltoidea | 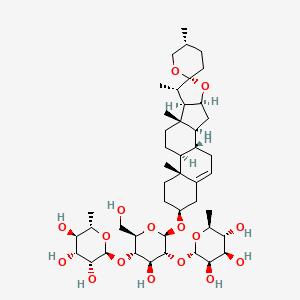 | NPC | Bcl-2, caspase-3, caspase-9 | Suppression of the TLR4/NF-kappaB pathway attenuates IL-1β-activated inflammatory and catabolic activity in human NPCs. | [68] |
| 69 | Naringin | Flavonoids | Salvia officinalis, Citrus reticulata | 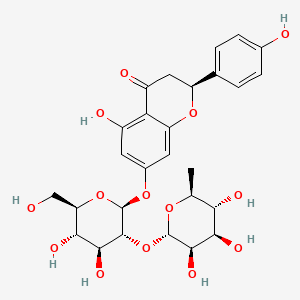 | NPC | Bcl-2, caspase-3, caspase-9 | Regulation of ROS-mediated PI3K/Akt pathway attenuates H_2_O_2_-induced NPMSC apoptosis and mitochondrial dysfunction. | [69] |
| 70 | Puerarin | Flavonoids | Bupleurum chinense, Pueraria calycina | 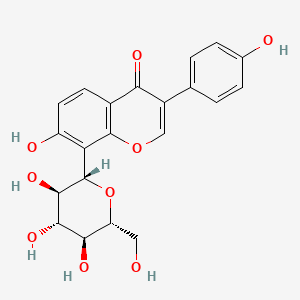 | NPC | Bcl-2, caspase-3, caspase-9 | Activation of the PI3K/Akt pathway stabilizes mitochondrial membrane potential and reduces ROS accumulation, thereby alleviating compression-induced apoptosis in vitro and in rat NPMSCs. | [70] |
| 71 | Sinapic Acid | Phenolic Acids | Poa huecu, Cynanchum thesioides | 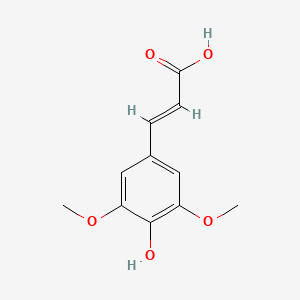 | NPC | Bcl-2, caspase-3, caspase-9 | Inhibition of the Nrf2/NF-κB signaling axis suppresses IL-1β-induced apoptosis in NP cells and reduces inflammatory mediator levels. Upregulation of type II collagen and aggrecan expression, and downregulation of matrix-degrading enzymes MMP13 and ADAMTS5. | [71] |
| 72 | Andrographolide | Terpenoids | Andrographis paniculata, Cymbopogon schoenanthus | 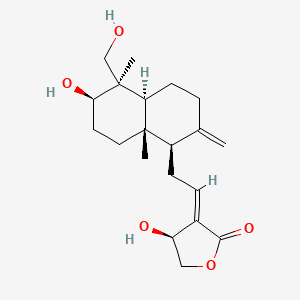 | NPC | / | Inhibition of the NF-κB pathway prevents LPS-induced degeneration of NPCs. | [72] |
| 73 | Berberine | Alkaloids | goldenseal, barberry, Oregon grape | 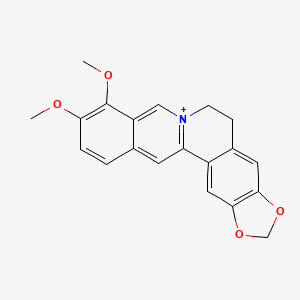 | NPC | Bcl-2, caspase-3, caspase-9, GRP78, caspase-12, CHOP | Modulation of ERS and autophagy prevents oxidative stress-induced apoptosis. | [73] |
| 74 | Kinsenoside | Phenanthrenes | Anoectochilus formosanus, Crocus sativus | 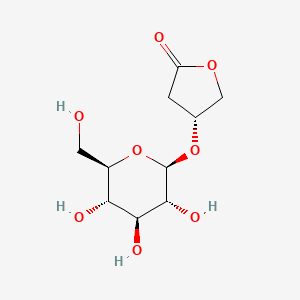 | NPC | caspase-3 | Activation of the AKT-ERK1/2-Nrf2 signaling pathway in NPCs exerts protective effects against apoptosis, senescence, and mitochondrial dysfunction. | [74] |
| 75 | Ligustilide | Terpenoids | Apiaceae, Angelica glauca | 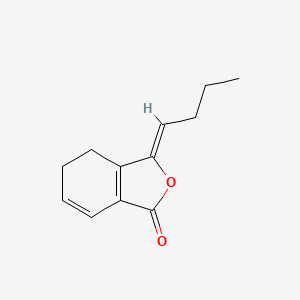 | NPC | BAX, Bcl-2, caspase-3 | Inhibition of the NF-κB signaling pathway suppresses inflammatory responses and IL-1β-induced apoptosis. | [75] |
| 76 | Glycyrrhizin | Terpenoids | Hypomontagnella monticulosa, Glycyrrhiza pallidiflora | 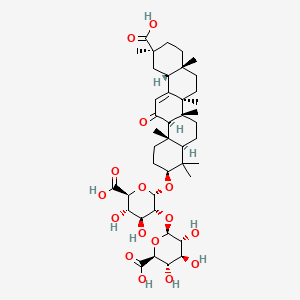 | NPC | caspase-3 | Inhibition of the p38/p-JNK signaling pathway suppresses HMGB1, attenuating IL-1β-induced inflammation and apoptosis, thereby inhibiting NP degradation. | [76] |
| 77 | Genistein | Flavonoids | Salvia hispanica, Glycine soja, | 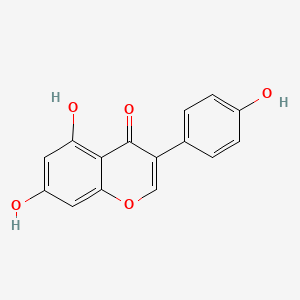 | NPC | BAX, Bcl-2, caspase-3 | Enhancement of nrf2-mediated antioxidant defense system rescues thbp-induced npc degeneration. | [77] |
| 78 | Berberine | Alkaloids | goldenseal, barberry, Oregon grape | 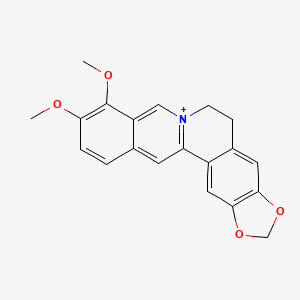 | NPC | BAX, Bcl-2, caspase-3 | Inhibition of the NF-κB pathway protects human NP cells from IL-1β-induced apoptosis. | [78] |
| 79 | ginsenoside Rg3 | Terpenoids | Panax ginseng, Panax japonicus | 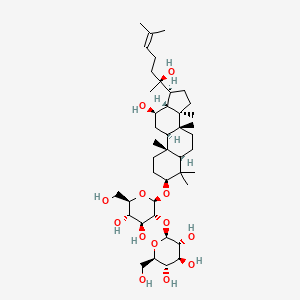 | NPC | BAX, Bcl-2, caspase-3 | Blockade of the NF-κB signaling pathway alleviates TNF-α-induced damage in NPCs. | [79] |
| 80 | Luteoloside | Flavonoids | Coreopsis lanceolata, Sonchus fruticosus | 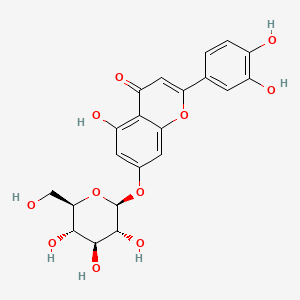 | NPC | BAX, Bcl-2, caspase-3 | Activation of the Nrf2/HO-1 signaling axis inhibits IL-1β-induced apoptosis in NPCs. | [80] |
| 81 | Andrographolide | Terpenoids | Andrographis paniculata, Cymbopogon schoenanthus | 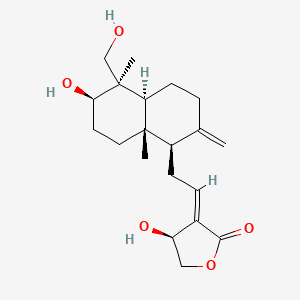 | NPC | BAX, Bcl-2, caspase-3 | Modulation of the TLR4/MyD88/NF-κB signaling pathway alleviates IL-1β-induced degeneration of NPCs. | [81] |
| 82 | Honokiol | Lignans | Magnolia officinalis, Illicium simonsii | 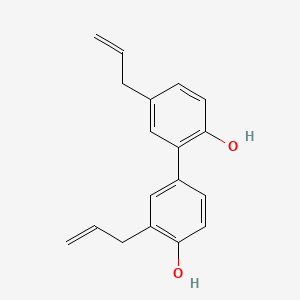 | NPC | BAX, caspase-3, caspase-9 | Inhibition of the TXNIP/NLRP3/caspase-1/IL-1β signaling axis and suppression of NF-κB/JNK activation mitigate H_2_O_2_-induced apoptosis, oxidative stress, and inflammatory responses. | [82] |
| 83 | Lupeol | Terpenoids | Camellia sinensis, Acanthus ilicifolius | 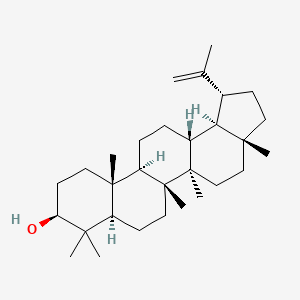 | NPC | BAX, Bcl-2, caspase-3, caspase-9 | Enhancement of mitochondrial antioxidant stress inhibits high glucose-induced apoptosis in NPCs. | [83] |
| 84 | Resveratrol | Stilbenes | Polygonum cuspidatum | 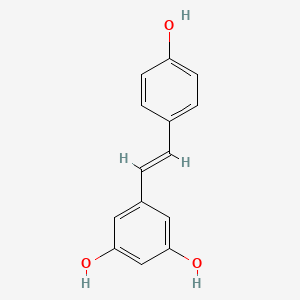 | NPC | / | Scavenging ROS inhibits SNP-induced apoptosis in NPCs. | [84] |
| 85 | Gallic acid | Phenolic Acids | Gallnuts, tea, grape | 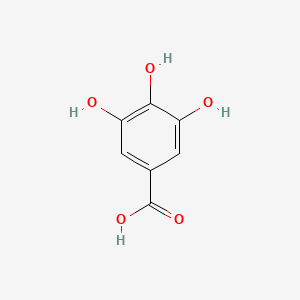 | NPC | / | Modulation of p65 phosphorylation and acetylation in the NF-κB signaling pathway reduces ADAMTS-4 expression and inhibits apoptosis in NPCs. | [85] |
| 86 | Icariin | Flavonoids | Epimedium brevicornu, Epimedium truncatum | 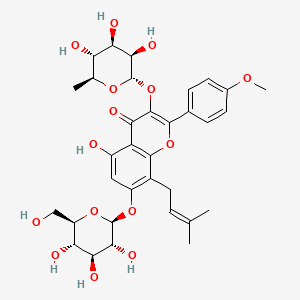 | NPC | BAX, Bcl-2, caspase-3 | Activation of the PI3K/AKT pathway alleviates H_2_O_2_-induced apoptosis in rat NPCs. | [86] |
| 87 | Icariin | Flavonoids | Epimedium brevicornu, Epimedium truncatum | 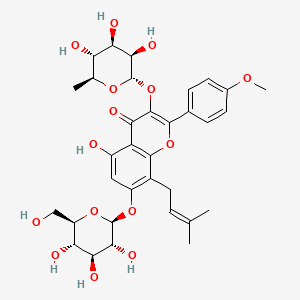 | NPC | BAX, Bcl-2, caspase-3 | Activation of the PI3K/AKT pathway alleviates IL-1β-induced apoptosis in rat NPCs. | [87] |
| 88 | Baicalin | Flavonoids | Scutellaria prostrata, Scutellaria scandens | 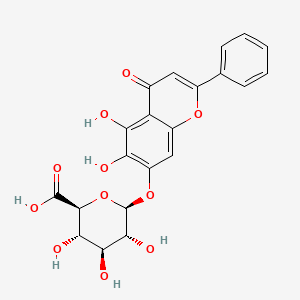 | CEPC | BAX, caspase-3 | Reduction of MDA levels and elevation of SOD/NO levels inhibits H2O2-induced oxidative stress in endplate chondrocytes and reduces apoptosis. | [88] |
| 89 | Plumbagin | Quinones | Ancistrocladus cochinchinensis, Ceratostigma willmottianum | 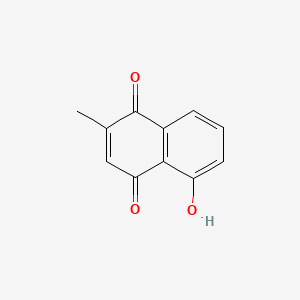 | NPC | caspase-3, caspase-9 | Modulation of NF-κB and Nrf-2 expression attenuates H2O2-induced oxidative stress, inflammation, and apoptosis in NPCs. | [89] |
| 90 | cannabidiol | Terpenoids | Cannabis | 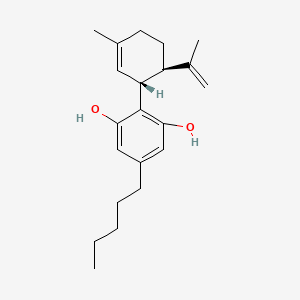 | NPC | Bcl-2, caspase-3 | Downregulation of caspase-3 gene expression and upregulation of Bcl-2 protein expression levels inhibits H2O2-induced apoptosis and inflammation in NPCs. | [90] |
| 91 | Paeoniflorin | Terpenoids | Paeonia emodi, Paeonia obovata | 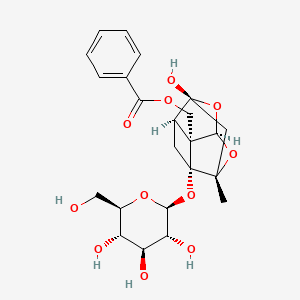 | NPC | BAX, Bcl-2, caspase-9 | Modulation of Bcl-2 family proteins and caspase-9 expression suppresses NPCs apoptosis. | [91] |
| 92 | Paeoniflorin | Terpenoids | Paeonia emodi, Paeonia obovata | 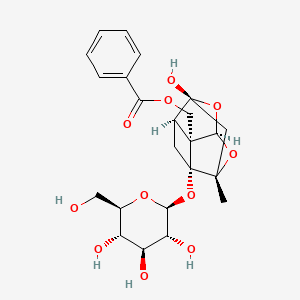 | AFC | Fas, caspase-3, | Inhibition of the Fasl - FasR signaling pathway activation reduces fasl-induced apoptosis in annulus fibrosus cells. | [92] |
| 93 | Aygdalin | Glycosides | Malus, Prunus salicina | 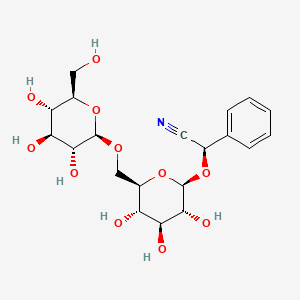 | CEPC | / | Combined with hydroxysafflor yellow inhibits IL-1β-induced apoptosis in NPCs | [93] |
| 94 | Orientin | Flavonoids | Cecropia hololeuca, Gentiana algid | 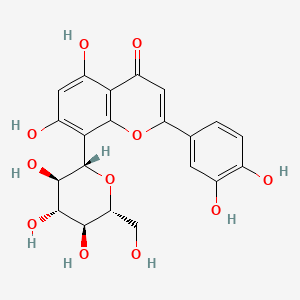 | NPC | BAX, Bcl-2, caspase-3, GRP78, CHOP | Upregulation of AMPK/SIRT1 restores ECM and ER homeostasis, thereby attenuating oxidative stress. | [94] |
| 95 | Lycopene | Terpenoids | Pyracantha angustifolia, Allomyces javanicus | 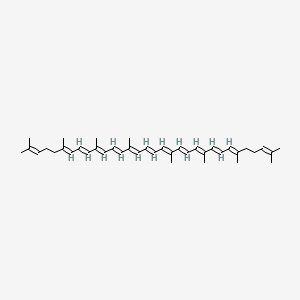 | NPC | BAX, Bcl-2 | Activation of Nrf2 attenuates H₂O₂-induced apoptosis in human degenerative NPCs. | [95] |

Table 2 Natural Products Targeting Inflammation and Oxidative Stress for IVDD

|  | Natural products | Chemical Class | Sources | Structure | Target cell types | Inflammation and oxidative stress-related mode of action | Key Regulatory Mechanisms | References |
| --- | --- | --- | --- | --- | --- | --- | --- | --- |
| 1 | Hyperforin | Terpenoids | Hypericum henryi, Hypericaceae | 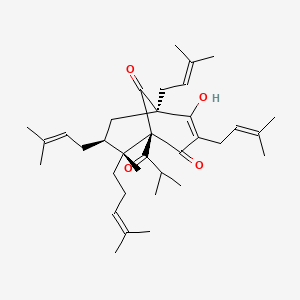 | NPC | P65, IL-17 | Activation of TRPC6 triggers mitochondrial fission-mediated suppression of PFKFB3 activity, thereby attenuating pro-inflammatory responses. | [96] |
| 2 | Myricetin | Flavonoids | Caragana frutex | 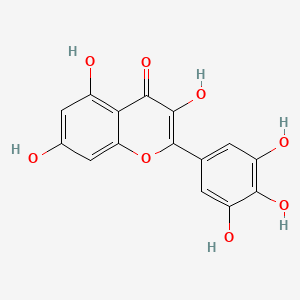 | NPC | iNOS, COX-2,P65, TNF-α, IL-6, PGE2 | Nrf2 activation inhibits NF-κB signaling to attenuate IL-1β-induced pro-inflammatory cytokine production in NPCs. | [97] |
| 3 | Evodiamine | alkaloids | Tetradium ruticarpum, Spiranthera odoratissima | 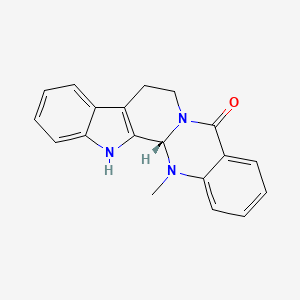 | NPC | iNOS, COX-2, | Dual modulation of the Nrf2/HO-1 and MAPK signaling pathways attenuates mitochondrial dysfunction, ECM degradation, and inflammatory responses. | [98] |
| 4 | Mulberroside A | Stilbenoids | Morus lhou, Veratrum dahuricum | 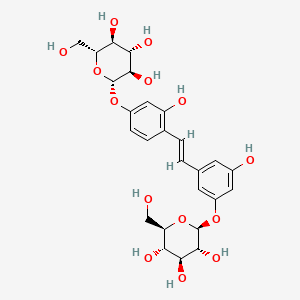 | NPC | iNOS, COX-2, IL-6 | Blockade of IL-1β-stimulated MAPK/NF-κB signaling transduction in NPCs attenuates inflammatory responses. | [99] |
| 5 | Vanillin | Benzoic Acid Derivatives | Vanilla planifolia | 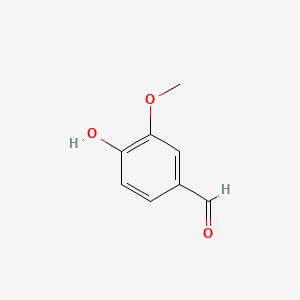 | NPC | IL-1β, IL-6, PTGS2, TNF-α | Dual modulation of the PI3K/Akt signaling pathway suppresses LPS-induced inflammatory responses and augments ECM anabolism in NPCs. | [100] |
| 6 | Demethoxycurcumin | Curcuminoids | Curcuma xanthorrhiza, Curcuma kwangsiensis | 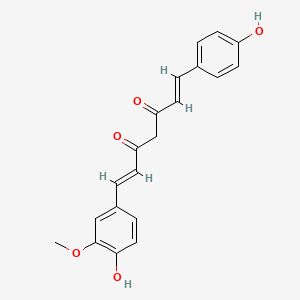 | NPC | IL-1β, IL-4, IL-6 | Suppression of TNF-α-induced inflammatory responses in NPCs downregulates the production of IL-1β, IL-4, and IL-6. | [101] |
| 7 | Ganoderic Acid A | Terpenoids | Ganoderma sinense, Ganoderma lucidum, and Wolfiporia cocos | 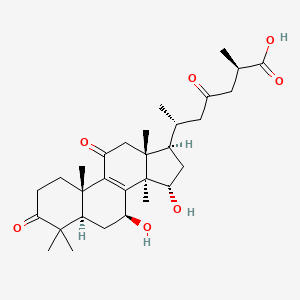 | NPC | iNOS, COX-2 | Targeted inhibition of the NF-κB signaling pathway attenuates IL-1β-induced inflammatory responses and ECM degradation in NPCs. | [102] |
| 8 | Curcumenol | Terpenoids | Curcuma xanthorrhiza, Artemisia annua |  | NPC | IL-1β, TNF-α | Targeted blockade of the TNF-α/NF-κB signaling pathway suppresses inflammatory responses in NPCs. | [103] |
| 9 | Rhizoma drynariae total flavonoids | / | / | / | NPC | IL-1β, TNF-α | Targeted inhibition of the MAPK signaling pathway downregulates the expression of matrix metalloproteinases (MMPs; e.g., MMP-3 and MMP-13) and pro-inflammatory cytokines such as IL-1β. | [104] |
| 10 | Curcumol | Terpenoids | Curcuma aromatica, Curcuma wenyujin, Cunninghamella blakesleeana |  | NPC | IL-1β, IL-6, TNF-α | Targeted modulation of the PI3K/Akt/NF-κB signaling axis attenuates inflammatory cascades and ameliorates IVDD progression. | [105] |
| 11 | Sodium Tanshinone IIA Sulfonate | Terpenoids | Salvia miltiorrhiza |  | NPC | IL-1β, IL-6, TNF-α | Inhibition of the p38 MAPK signaling pathway attenuates acupuncture-induced oxidative stress and concurrently elicits anti-inflammatory and antioxidant effects. | [106] |
| 12 | Cardamonin | Flavonoids | Cedrelopsis grevei, Boesenbergia rotunda |  | NPC | iNOS, COX-2, PGE2, NO, IL-6, TNF-α | Activation of the Nrf2/HO-1 signaling axis attenuates inflammatory responses through redox homeostasis modulation. | [107] |
| 13 | Oxymatrine | Alkaloids | Sophora macrocarpa, Sophora chrysophylla |  | NPC | IL-1β, TNF-α | Targeted inhibition of the TLR4/NF-κB signaling axis attenuates inflammatory responses in NPCs and mitigates ECM degradation in nucleus pulposus tissues. | [108] |
| 14 | Acacetin | Flavonoids | Caragana frutex, Crocus heuffelianus |  | NPC | COX-2, iNOS | Activation of the Nrf2 signaling pathway suppresses phosphorylation of p38, JNK, and ERK1/2, thereby attenuating THBP-induced ROS generation and inflammatory mediator production, while suppressing ECM degradation. | [109] |
| 15 | Procyanidin B3 | Flavonoids | Camellia sinensis, Paeonia obovata |  | NPC | TNF-α, IL-6, NO, PEG2 | Suppression of LPS-induced pro-inflammatory cytokine production via blockade of NF-κB/TLR4 signaling pathway activation. | [110] |
| 16 | Stachydrine | alkaloids | Leonurus japonicus, Achillea setacea |  | NPC | IL-1β, iNOS, COX-2 | Targeted blockade of IL-1β-induced PI3K/Akt/NF-κB signaling cascade activation downregulates pro-inflammatory cytokine expression. | [111] |
| 17 | Higenamine | alkaloids | [Gnetum montanum](https://pubchem.ncbi.nlm.nih.gov/taxonomy/3381#section=Natural-Products), [Gnetum parvifolium](https://pubchem.ncbi.nlm.nih.gov/taxonomy/33153#section=Natural-Products) |  | NPC | iNOS, COX-2, PGE2, NO, IL-6, TNF-α | Targeted inhibition of the NF-κB signaling pathway attenuates IL-1β-induced inflammatory responses in NPCs. | [112] |
| 18 | Baicalein | Flavonoids | Scutellaria baicalensis |  | NPC | iNOS, COX-2, PGE2, NO, IL-6, TNF-α | Pharmacological intervention reverses IL-1β-induced overexpression of MMP-13 and ADAMTS5, ameliorates aggrecan and type II collagen degradation, and concurrently suppresses IL-1β-triggered activation of NF-κB and MAPK signaling pathways. | [113] |
| 19 | Aucubin | Terpenoids | Veronica kellereri, Plantago uniflora |  | NPC | TNF-α, IL-1β | Regulation of miR-140/CREB1 improves IL-1β- or TNF-α-induced ECM degradation in HNPCs. | [114] |
| 20 | Naringin | Flavonoids | Salvia officinalis, Citrus reticulata |  | NPC | TNF-α, IL-6, ikappabα | Suppression of NF-κB signaling and p53 expression in IL-1β-treated HNPCs attenuates MMP-mediated catabolic remodeling and inflammatory responses. | [115] |
| 21 | Moracin | Flavonoids | Morus lhou, Artocarpus tonkinensis |  | NPC | TNF-α, IL-6, IL-1β | Partial modulation of the Nrf2/HO-1 and NF-κB/TGF-β pathways counteracts LPS-mediated inflammatory responses in NPCs. | [116] |
| 22 | Halofuginone | alkaloids | Dichroa febrifuga |  | NPC | TNF-α, IL-6,IL-8 | Targeted suppression of type I collagen biosynthesis and TGF-β/NF-κB signaling activation in degenerative nucleus pulposus attenuates IVDD progression. | [117] |
| 23 | Wogonin | Flavonoids | Trichoderma virens, Rhinacanthus nasutus |  | NPC | iNOS, COX-2, IL-6 | Through activation of the Nrf2/HO-1-SOD2-NQO1-GCLC signaling axis, it exerts anti-inflammatory effects. | [118] |
| 24 | Icariin | Flavonoids | Epimedium brevicornu, Epimedium truncatum |  | NPC | iNOS, COX-2, PGE2, NO | By inhibiting IL-1β-induced activation of MAPK and NF-κB-related signaling pathways, it exerts anti-inflammatory effects. | [119] |
| 25 | Pilose antler peptide | / | Pilose Antler | / | NPC | TNF-α, IL-6, IL-1β | Counteracting LPS-induced inflammatory responses in nucleus pulposus cells. | [120] |
| 26 | Celastrol | Terpenoids | Celastrus paniculatus, Tripterygium wilfordii |  | NPC | TNF-α, IL-6, iNOS, COX-2 | Suppression of the NF-κB signaling pathway attenuates IL-1β-induced ECM catabolism, oxidative stress, and inflammatory responses in HNPCs. | [121] |
| 27 | Carthamin yellow | Flavonoids | Carthamus tinctorius |  | NPC | TNF-α, | Inhibition of the MAPK signaling pathway potentiates anti-inflammatory effects in LPS-induced NPCs. | [122] |
| 28 | Sesamin | Lignans | Otanthus maritimus, Apis |  | NPC | IL-1β, TNF-α, iNOS, NO, COX-2, PGE2 | Suppression of JNK phosphorylation, a common downstream signaling event of LPS and IL-1β, inhibits LPS-induced MAPK signaling pathway activation, thereby protecting IVDs from inflammatory damage and ECM catabolism. | [123] |
| 29 | Cordycepin | nucleosides | Cordyceps militaris, Streptomyces sparsogenes |  | NPC | iNOS, PGE2 | Inhibition of NF-κB signaling pathway activation exerts anti-inflammatory and anti-degenerative effects on NPCs and IVDs. | [124] |
| 30 | curcumin | Diarylheptanoids | Curcuma longa |  | NPC | TNF-α | Suppression of NF-κB p65 nuclear translocation inhibits NF-κB activation by reducing pro-inflammatory cytokine release, thereby attenuating IVDD progression. | [125] |
| 31 | Piperine | alkaloids | Periconia, Piper khasianum |  | NPC | TNF-α, IL-6, IL-1β, iNOS | Inhibition of JNK phosphorylation and NF-κB activation counteracts LPS -induced inflammatory responses in NPCs. | [126] |
| 32 | Tanshinone IIA | Terpenoids | Salvia miltiorrhiza, Salvia glutinosa |  | NPC | IL-6, IL-8, iNOS, COX-2, TLR2 | By modulating the activity of IRAK-1 and its downstream effectors p38, JNK, and NF-κB, it significantly inhibits the expression of pro-inflammatory mediators and matrix metalloproteinases . | [127] |
| 33 | Crocin | Terpenoids | Gardenia jasminoides, Crocus sativus |  | NPC | TNF-α, IL-6, IL-1β, iNOS,TLR2 | By inhibiting the JNK/MAPK pathway, it suppresses degenerative inflammation in nucleus pulposus cells. | [128] |
| 34 | Epigallocatechin 3-gallate | Flavonoids | Camellia sinensis, Eschweilera coriacea |  | NPC | IL-1β | Targeting IRAK-1 and its downstream effectors p38, JNK, and NF-κB leads to suppression of pro-inflammatory mediators and matrix metalloproteinase expression. | [129] |
| 35 | Lycorine | alkaloids | Crinum moorei, Clivia nobilis |  | CEPC | IL-1β | Suppression of NF-κB signaling attenuates IL-1β-induced endplate cell degeneration by downregulating pro-inflammatory cytokine expression and reducing ECM catabolic remodeling. | [130] |
| 36 | Muscone | Terpenoids | Moschus moschiferus |  | CEPC | IL-1β, TNF-α, PGE2 | Inhibition of the expression of PGE2, IL-1β, and TNF-α. | [131] |
| 37 | kaempferol | Flavonoids | Hydrangea serrata, Caragana frutex |  | NPC | NLRP3, IL-1β | Suppression of LPS-induced inflammatory response in NPCs. | [132] |

Table 3 Natural Products Targeting cellular senescence for IVDD

|  | Natural products | Chemical Class | Sources | Structure | Target cell types | Cellular senescence-related mode of action | Key Regulatory Mechanisms | References |
| --- | --- | --- | --- | --- | --- | --- | --- | --- |
| 1 | Maslinic acid | Terpenoids | Salvia miltiorrhiza, Sideritis candicans |  | NPC | P16, P21 | It upregulates aggrecan and Collagen II synthesis while downregulating MMP-3/ADAMTS-5 expression in NPCs, restoring ECM homeostasis and attenuating cellular senescence. | [133] |
| 2 | Quercetin | Flavonoids | Tea, tomatoes, cherries, grapes, apples,onions |  | NPMSC | P16, P21, P53 | The miR-34a/SIRT1 signaling axis mitigates oxidative stress-induced senescence in NPMSCs by enhancing antioxidant defense and suppressing p53/p21-mediated cell cycle arrest. | [134] |
| 3 | Myricetin | Flavonoids | Caragana frutex |  | NPC | SA-β-Gal, P21, P16, IL-6, IL-8 | Modulation of SERPINE1 attenuates HO-induced cellular senescence in NPCs by suppressing oxidative stress and senescence-associated β-galactosidase (SA-β-gal) activity. | [135] |
| 4 | Dihydroartemisinin | Terpenoids | Ganoderma colossus，Acronychia pubescens | / | NPC | P16, P21 | Suppression of PI3K/AKT and NF-κB signaling pathways restores ECM anabolic-catabolic balance and mitigates TNF-α-induced cellular senescence inNPCs. | [136] |
| 5 | p-Coumaric acid | phenolic acids | Camellia sinensis, Camellia reticulata |  | NPC | P16, P53, SA-β-Gal | Downregulation of p16 and p53 protein expression, combined with suppression of (SA-β-gal activity, attenuates H₂O₂-induced oxidative stress and cellular senescence in NPCs. | [137] |
| 6 | Quercetin | Flavonoids | Tea, tomatoes, cherries, grapes, apples,onions |  | NPC | P16, P21, IL-6, IL-8 | Inhibition of SASP factor expression in IL-1β-treated NPCs attenuates cellular senescence , while modulation of the Nrf2/NF-κB signaling axis mitigates IVDD. progression via ECM homeostasis restoration. | [138] |
| 7 | o-vanillin | Benzoic Acid Derivatives | Strychnos cathayensis, Hyssopus officinalis, Panax ginseng |  | NPC | P16, P21 | Reduction of SASP factors and TLR-2 gene and protein expression. | [139] |
| 8 | Dehydrocostus Lactone | Terpenoids | Ainsliaea uniflora, Costus |  | NPC | P53, P21 | Inhibition of NF-κB and MAPK inflammatory signaling pathway activation alleviates TNF-α-induced STING-TBK1/NF-κB signaling hyperactivation-mediated senescence in NPCs. | [140] |
| 9 | Eupatilin | Flavonoids | Artemisia princeps, Achillea setacea |  | NPC | P21, P53 | Suppression of MAPK/NF-κB signaling pathway activation attenuates ECM degradation and ameliorates cellular senescence. | [141] |

Table 4 Natural Products Targeting Other Cell Phenotypes for IVDD

|  | Natural products | Chemical Class | Sources | Structure | Target cell types | Other Cell Phenotypes-related mode of action | Key Regulatory Mechanisms | References |
| --- | --- | --- | --- | --- | --- | --- | --- | --- |
| 1 | crocetin | Terpenoids | Gardenia jasminoides, Perilla frutescens |  | NPC | ROS | Suppression of oxidative stress damage in NPCs. | [142] |
| 2 | Ascorbic acid | Organic Acids | Oranges, lemons, grapefruits, limes, Broccoli, kale, Brussels sprouts |  | NPC | SLC23A2, ALDH1A3 | Upregulation of ALDH1A3 expression counteracts time-dependent degeneration of NPCs and restores the maintenance of NPC proliferation. | [143] |
| 3 | Cyanidin | Flavonoids | Camellia sinensis, Viburnum rafinesquianum |  | NPC | / | Pharmacological blockade of the Wnt/β-catenin signaling pathway attenuates high hydrostatic pressure (HHP)-induced ECM degradation in HNPCs by suppressing matrix-degrading enzyme activity and preserving collagen II/aggrecan biosynthesis. | [144] |
| 4 | Resveratrol | Stilbenes | Polygonum cuspidatum |  | NPC | / | Regulation of ECM expression in NPCs through the Wnt/β-catenin signaling pathway. | [145] |
| 5 | naringin | Flavonoids | Salvia officinalis, Citrus reticulata |  | NPC | TNF-α, BMP-2 | Upregulation of aggrecan, BMP-2, and Sox6 expression combined with downregulation of TNF-α and MMP-3 expression effectively promotes the proliferation of degenerative NPCs. | [146] |
| 6 | Epigallocatechin | Flavonoids | Camellia sinensis, Eschweilera coriacea |  | NPC | P53, P21 | Activation of the pro-survival PI3K/AKT pathway and preservation of mitochondrial integrity enhance the survival rate of IVD cells under lethal oxidative stress. | [147] |
| 7 | resveratrol | Stilbenes | Polygonum cuspidatum |  | NPC | SIRT1 | Upregulation of SIRT1 expression promotes ECM synthesis in NPCs | [148] |
| 8 | Salvianolic acid A | Phenolic Acids | Salvia miltiorrhiza, Celastrus hindsii |  | CEBP | / | Targeted modulation of miR-940 and miR-576-5p attenuates IL-1β-induced extracellular matrix (ECM) degradation in CEPCs. | [149] |
| 9 | hesperidin | Flavonoids | Humulus lupulus, Ficus erecta var. beecheyana |  | NPC | Inos, cox2 | By reducing the expression of CRISP2, iNOS, and COX2, decreasing reactive oxygen species (ROS) and apoptosis, and lowering inflammatory markers. | [150] |
| 10 | Violina pumpkin leaf | / | / | / | NPC | SOD2, OCT2, SOX2 | The treatment markedly upregulated chondrogenic transcription factors (SOX9, TRPS1), ECM components (aggrecan, collagen II), and regulators of cellular homeostasis/stress response (FOXO3a, NRF2, SOD2, SIRT1). It effectively mitigated H₂O₂-induced cellular damage and suppressed the pro-inflammatory/anti-chondrogenic microRNA, miR-221. | [151] |
| 11 | Naringin | Flavonoids | Salvia officinalis, Citrus reticulata |  | NPC | IL-6, IL-10, caspase-3, ACAN, COL1A1, COL11A1, IGF1R, SPARC,Parkin | Upregulation of extracellular matrix components , anti-inflammatory mediators , interleukin receptor complexes, matrix remodeling enzymes, growth factor signaling , matricellular proteins , mitophagy regulators , vitamin D metabolism, and anti-apoptotic factors (BCL2); concurrent downregulation of pro-inflammatory cytokines and apoptotic executors. | [152] |
| 12 | Ligustrazine | alkaloids | [Francisella tularensis](https://pubchem.ncbi.nlm.nih.gov/taxonomy/263#section=Natural-Products), [Camellia sinensis](https://pubchem.ncbi.nlm.nih.gov/taxonomy/4442#section=Natural-Products) |  | NPC | CCN2, ACAN | Targeted suppression of TGF-β signaling hyperactivation in NPCs mitigates IVDD progression by preserving matrix homeostasis. | [153] |
| 13 | curcumin | Diarylheptanoids | Curcuma longa |  | NPC | iNOS, COX-2, IL-1β | Modulation of iNOS, COX-2, TGF-β1/2, MMP-9, and BDNF exerts protective effects against IVDD development. | [154] |
| 14 | curcumin | Diarylheptanoids | Curcuma longa |  | NPC | iNOS, COX-2, IL-1β | Modulation of iNOS, COX-2, TGF-β1/2, MMP-9, and BDNF exerts protective effects against IVDD development. | [154] |

**References：**

[1] CHEN J, ZHANG B, WU L, et al. Ginsenoside Rg3 exhibits anti-catabolic and anti-apoptotic effects in IL-1β treated human disc nucleus pulposus cells and in a rat model of disc degeneration by inactivating the MAPK pathway [J]. Cell Mol Biol (Noisy-le-grand), 2024, 70(1): 233-8.

[2] YU L, HAO Y-J, REN Z-N, et al. Ginsenoside Rg1 relieves rat intervertebral disc degeneration and inhibits IL-1β-induced nucleus pulposus cell apoptosis and inflammation via NF-κB signaling pathway [J]. In Vitro Cell Dev Biol Anim, 2024, 60(3): 287-99.

[3] WANG C-M-J, WU Y-D, LIANG S-L, et al. [Mechanism of kaempferol on intervertebral disc degeneration based on p38 MAPK signaling pathway] [J]. Zhongguo Zhong Yao Za Zhi, 2024, 49(21): 5721-9.

[4] CHEN E, LI M, LIAO Z, et al. Phillyrin reduces ROS production to alleviate the progression of intervertebral disc degeneration by inhibiting NF-κB pathway [J]. Journal of Orthopaedic Surgery and Research, 2024, 19(1): 308.

[5] HUANG Y, SUN J, LI S, et al. Isoliquiritigenin mitigates intervertebral disc degeneration induced by oxidative stress and mitochondrial impairment through a PPARγ-dependent pathway [J]. Free Radic Biol Med, 2024, 225.

[6] XIA J, JIA D, WU J. Protective effects of alpinetin against interleukin-1β-exposed nucleus pulposus cells: Involvement of the TLR4/MyD88 pathway in a cellular model of intervertebral disc degeneration [J]. Toxicol Appl Pharmacol, 2024, 492: 117110.

[7] HUANG Y, LEI L, ZHAO Z, et al. Acetylshikonin promoting PI3K/Akt pathway and inhibiting SOX4 expression to delay intervertebral disc degeneration and low back pain [J]. Journal of orthopaedic research : official publication of the Orthopaedic Research Society, 2024, 42(1): 172-82.

[8] TENG C, WU J, ZHANG Z, et al. Fucoxanthin ameliorates endoplasmic reticulum stress and inhibits apoptosis and alleviates intervertebral disc degeneration in rats by upregulating Sirt1 [J]. Phytother Res, 2024, 38(5): 2114-27.

[9] WANG X, SONG C, ZHOU D, et al. Exploring the therapeutic potential of puerarin on intervertebral disc degeneration by regulating apoptosis of nucleus pulposus cells [J]. JOR Spine, 2024, 7(4): e70020.

[10] REN J, XIN R, CUI X, et al. Quercetin relieves compression-induced cell death and lumbar disc degeneration by stabilizing HIF1A protein [J]. Heliyon, 2024, 10(17): e37349.

[11] LEI L, WANG H, ZHAO Z, et al. Curculigoside upregulates BMAL1 to decrease nucleus pulposus cell apoptosis by inhibiting the JAK/STAT3 pathway [J]. Osteoarthritis Cartilage, 2024.

[12] LI L-H, WANG S-Q, SUN K, et al. [Mechanism of aucubin in regulating ribosome biogenesis and inhibiting injury of nucleus pulposus cells and extracellular matrix degradation] [J]. Zhongguo Zhong Yao Za Zhi, 2024, 49(21): 5713-20.

[13] ZHANG C, LU Z, LYU C, et al. Andrographolide Inhibits Static Mechanical Pressure-Induced Intervertebral Disc Degeneration via the MAPK/Nrf2/HO-1 Pathway [J]. Drug Des Devel Ther, 2023, 17: 535-50.

[14] HU B, LIN S, LIN S, et al. Ginkgetin Alleviates Intervertebral Disc Degeneration by Inhibiting Apoptosis, Inflammation, and Disturbance of Extracellular Matrix Synthesis and Catabolism via Inactivation of NLRP3 Inflammasome [J]. Immunological Investigations, 2023, 52(5): 546-60.

[15] YANG L, LI Z, ZHANG C, et al. Psoralen synergizes with exosome-loaded SPC25 to alleviate senescence of nucleus pulposus cells in intervertebral disc degeneration [J]. Journal of Orthopaedic Surgery and Research, 2023, 18(1): 622.

[16] ZOU Y-P, ZHANG Q-C, ZHANG Q-Y, et al. Procyanidin B2 alleviates oxidative stress-induced nucleus pulposus cells apoptosis through upregulating Nrf2 via PI3K-Akt pathway [J]. Journal of orthopaedic research : official publication of the Orthopaedic Research Society, 2023, 41(7): 1555-64.

[17] ZHU X, GUO S, ZHANG M, et al. Emodin protects against apoptosis and inflammation by regulating reactive oxygen species-mediated NF-κB signaling in interleukin-1β-stimulated human nucleus pulposus cells [J]. Hum Exp Toxicol, 2023, 42: 9603271221138552.

[18] LU X, XU G, LIN Z, et al. Sulforaphane Delays Intervertebral Disc Degeneration by Alleviating Endoplasmic Reticulum Stress in Nucleus Pulposus Cells via Activating Nrf-2/HO-1 [J]. Oxidative Medicine and Cellular Longevity, 2023, 2023: 3626091.

[19] LIU Y, LIU D-K, WANG Z-W, et al. Baicalein alleviates TNF-α-induced apoptosis of human nucleus pulposus cells through PI3K/AKT signaling pathway [J]. Journal of Orthopaedic Surgery and Research, 2023, 18(1): 292.

[20] ZHOU Q, ZHU C, XUAN A, et al. Fisetin regulates the biological effects of rat nucleus pulposus mesenchymal stem cells under oxidative stress by sirtuin-1 pathway [J]. Immun Inflamm Dis, 2023, 11(5): e865.

[21] LU G, ZHANG C, LI K, et al. Sinomenine Ameliorates IL-1β-Induced Intervertebral Disc Degeneration in Rats Through Suppressing Inflammation and Oxidative Stress via Keap1/Nrf2/NF-κB Signaling Pathways [J]. J Inflamm Res, 2023, 16: 4777-91.

[22] YAO D, LI M, WANG K, et al. Emodin ameliorates matrix degradation and apoptosis in nucleus pulposus cells and attenuates intervertebral disc degeneration through LRP1 in vitro and in vivo [J]. Exp Cell Res, 2023, 432(2): 113794.

[23] WANG X, TAN Y, LIU F, et al. Pharmacological network analysis of the functions and mechanism of kaempferol from Du Zhong in intervertebral disc degeneration (IDD) [J]. J Orthop Translat, 2023, 39: 135-46.

[24] ZHANG L, GAO J, LI Z, et al. Astragaloside IV relieves IL-1β-induced human nucleus pulposus cells degeneration through modulating PI3K/Akt signaling pathway [J]. Medicine, 2023, 102(33): e34815.

[25] BAI X, YAO M, ZHU X, et al. Baicalin suppresses interleukin-1β-induced apoptosis, inflammatory response, oxidative stress, and extracellular matrix degradation in human nucleus pulposus cells [J]. Immunopharmacol Immunotoxicol, 2023, 45(4): 433-42.

[26] YUAN L, MIAO H, DING H, et al. Polyphyllin I suppressed the apoptosis of intervertebral disc nucleus pulposus cells induced by IL-1β by miR-503-5p/Bcl-2 axis [J]. Journal of Orthopaedic Surgery and Research, 2023, 18(1): 466.

[27] YANG L, LI Z-Y, MA L, et al. [Aucubin combined with ADSCs-exos protects TBHP-induced nucleus pulposus cells via TLR4/NF-κB pathway] [J]. Zhongguo Zhong Yao Za Zhi, 2023, 48(19): 5294-303.

[28] HUANG C, ZOU K, WANG Y, et al. Esculetin Alleviates IL-1β-Evoked Nucleus Pulposus Cell Death, Extracellular Matrix Remodeling, and Inflammation by Activating Nrf2/HO-1/NF-kb [J]. ACS Omega, 2024, 9(1): 817-27.

[29] YANG Y-H, GU X-P, HU H, et al. Ginsenoside Rg1 inhibits nucleus pulposus cell apoptosis, inflammation and extracellular matrix degradation via the YAP1/TAZ pathway in rats with intervertebral disc degeneration [J]. Journal of Orthopaedic Surgery and Research, 2022, 17(1): 555.

[30] YANG L, LI Z, OUYANG Y. Taurine attenuates ER stress‑associated apoptosis and catabolism in nucleus pulposus cells [J]. Molecular Medicine Reports, 2022, 25(5).

[31] JI Z, GUO R, MA Z, et al. Arctigenin inhibits apoptosis, extracellular matrix degradation, and inflammation in human nucleus pulposus cells by up-regulating miR-483-3p [J]. Journal of clinical laboratory analysis, 2022, 36(7): e24508.

[32] TIAN Y, CHU X, HUANG Q, et al. Astragaloside IV attenuates IL-1β-induced intervertebral disc degeneration through inhibition of the NF-κB pathway [J]. Journal of Orthopaedic Surgery and Research, 2022, 17(1): 545.

[33] KUAI J, ZHANG N. Upregulation of SIRT1 by Evodiamine activates PI3K/AKT pathway and blocks intervertebral disc degeneration [J]. Molecular Medicine Reports, 2022, 26(2).

[34] WANG D, CAI X, XU F, et al. Ganoderic Acid A alleviates the degeneration of intervertebral disc via suppressing the activation of TLR4/NLRP3 signaling pathway [J]. Bioengineered, 2022, 13(5): 11684-93.

[35] ZHANG Y-H, SHANGGUAN W-J, ZHAO Z-J, et al. Naringin Inhibits Apoptosis Induced by Cyclic Stretch in Rat Annular Cells and Partially Attenuates Disc Degeneration by Inhibiting the ROS/NF-κB Pathway [J]. Oxidative Medicine and Cellular Longevity, 2022, 2022: 6179444.

[36] CHEN J, BIAN M, PAN L, et al. α-Mangostin protects lipopolysaccharide-stimulated nucleus pulposus cells against NLRP3 inflammasome-mediated apoptosis via the NF-κB pathway [J]. J Appl Toxicol, 2022, 42(9): 1467-76.

[37] BAI X, JIANG M, WANG J, et al. Cyanidin attenuates the apoptosis of rat nucleus pulposus cells and the degeneration of intervertebral disc via the JAK2/STAT3 signal pathway in vitro and in vivo [J]. Pharm Biol, 2022, 60(1): 427-36.

[38] XIE T, YUAN J, MEI L, et al. Hyperoside ameliorates TNF‑α‑induced inflammation, ECM degradation and ER stress‑mediated apoptosis via the SIRT1/NF‑κB and Nrf2/ARE signaling pathways in vitro [J]. Molecular Medicine Reports, 2022, 26(2).

[39] WANG D, QU H, KANG H, et al. Kukoamine A attenuates lipopolysaccharide-induced apoptosis, extracellular matrix degradation, and inflammation in nucleus pulposus cells by activating the P13K/Akt pathway [J]. Bioengineered, 2022, 13(4): 8772-84.

[40] CHEN H-W, LIU M-Q, ZHANG G-Z, et al. Proanthocyanidins inhibit the apoptosis and aging of nucleus pulposus cells through the PI3K/Akt pathway delaying intervertebral disc degeneration [J]. Connect Tissue Res, 2022, 63(6): 650-62.

[41] WANG Z, ZHANG P, ZHAO Y, et al. Scutellarin Protects Against Mitochondrial Reactive Oxygen Species-Dependent NLRP3 Inflammasome Activation to Attenuate Intervertebral Disc Degeneration [J]. Front Bioeng Biotechnol, 2022, 10: 883118.

[42] XIE T, YUAN J, MEI L, et al. Luteolin suppresses TNF-α-induced inflammatory injury and senescence of nucleus pulposus cells via the Sirt6/NF-κB pathway [J]. Experimental and Therapeutic Medicine, 2022, 24(1): 469.

[43] YANG S, LIAO W. Hydroxysafflor yellow A attenuates oxidative stress injury-induced apoptosis in the nucleus pulposus cell line and regulates extracellular matrix balance via CA XII [J]. Experimental and Therapeutic Medicine, 2022, 23(2): 182.

[44] CHEN T, LI P, QIU J, et al. Aloin Regulates Matrix Metabolism and Apoptosis in Human Nucleus Pulposus Cells via the TAK1/NF-κB/NLRP3 Signaling Pathway [J]. Stem Cells Int, 2022, 2022: 5865011.

[45] BAI X, LIAN Y, HU C, et al. Cyanidin-3-glucoside protects against high glucose-induced injury in human nucleus pulposus cells by regulating the Nrf2/HO-1 signaling [J]. J Appl Toxicol, 2022, 42(7): 1137-45.

[46] ZHENG Y, CHEN X, LAN T, et al. Panax notoginseng saponin reduces IL-1β-stimulated apoptosis and endoplasmic reticulum stress of nucleus pulposus cells by suppressing miR-222-3p [J]. Ann Transl Med, 2022, 10(13): 748.

[47] HU H, LI L, LIU Y, et al. [Effect of resveratrol on high mobility group box-1 protein signaling pathway in cartilage endplate degeneration caused by inflammation] [J]. Zhongguo Xiu Fu Chong Jian Wai Ke Za Zhi, 2022, 36(4): 461-9.

[48] ZHU X, LIU S, CAO Z, et al. Higenamine mitigates interleukin-1β-induced human nucleus pulposus cell apoptosis by ROS-mediated PI3K/Akt signaling [J]. Mol Cell Biochem, 2021, 476(11): 3889-97.

[49] DAI S, LIANG T, SHI X, et al. Salvianolic Acid B Protects Intervertebral Discs from Oxidative Stress-Induced Degeneration via Activation of the JAK2/STAT3 Signaling Pathway [J]. Oxidative Medicine and Cellular Longevity, 2021, 2021: 6672978.

[50] YU H, HOU G, CAO J, et al. Mangiferin Alleviates Mitochondrial ROS in Nucleus Pulposus Cells and Protects against Intervertebral Disc Degeneration via Suppression of NF-κB Signaling Pathway [J]. Oxidative Medicine and Cellular Longevity, 2021, 2021: 6632786.

[51] DU X, WANG X, CUI K, et al. Tanshinone IIA and Astragaloside IV Inhibit miR-223/JAK2/STAT1 Signalling Pathway to Alleviate Lipopolysaccharide-Induced Damage in Nucleus Pulposus Cells [J]. Dis Markers, 2021, 2021: 6554480.

[52] MEI L, ZHENG Y, MA T, et al. (-)-Epigallocatechin-3-gallate Ameliorates Intervertebral Disc Degeneration Through Reprogramming of the Circadian Clock [J]. Front Pharmacol, 2021, 12: 753548.

[53] HONG H, XIAO J, GUO Q, et al. Cycloastragenol and Astragaloside IV activate telomerase and protect nucleus pulposus cells against high glucose-induced senescence and apoptosis [J]. Experimental and Therapeutic Medicine, 2021, 22(5): 1326.

[54] BAI X, GUO X, ZHANG F, et al. Resveratrol Combined with 17β-Estradiol Prevents IL-1β Induced Apoptosis in Human Nucleus Pulposus Via The PI3K/AKT/Mtor and PI3K/AKT/GSK-3β Pathway [J]. J Invest Surg, 2021, 34(8): 904-11.

[55] ZHANG G, WANG H, ZHANG Q, et al. Bergenin alleviates H2 O2 -induced oxidative stress and apoptosis in nucleus pulposus cells: Involvement of the PPAR-γ/NF-κB pathway [J]. Environ Toxicol, 2021, 36(12): 2541-50.

[56] LIU Y, ZHENG J, CHEN Y, et al. Shikonin protects against lipopolysaccharide-induced inflammation and apoptosis in human nucleus pulposus cells through the nuclear factor-kappa B pathway [J]. Food Sci Nutr, 2021, 9(10): 5583-9.

[57] SHAN Q, LI N, ZHANG F, et al. Resveratrol Suppresses Annulus Fibrosus Cell Apoptosis through Regulating Oxidative Stress Reaction in an Inflammatory Environment [J]. BioMed Research International, 2021, 2021: 9100444.

[58] GE Q, YING J, SHI Z, et al. Chlorogenic Acid retards cartilaginous endplate degeneration and ameliorates intervertebral disc degeneration via suppressing NF-κB signaling [J]. Life Sciences, 2021, 274: 119324.

[59] WANG H, DING Y, ZHANG W, et al. Oxymatrine Liposomes for Intervertebral Disc Treatment: Formulation, in vitro and vivo Assessments [J]. Drug Des Devel Ther, 2020, 14: 921-31.

[60] XIANG Q, CHENG Z, WANG J, et al. Allicin Attenuated Advanced Oxidation Protein Product-Induced Oxidative Stress and Mitochondrial Apoptosis in Human Nucleus Pulposus Cells [J]. Oxidative Medicine and Cellular Longevity, 2020, 2020: 6685043.

[61] YU L, HAO Y, PENG C, et al. Effect of Ginsenoside Rg1 on the intervertebral disc degeneration rats and the degenerative pulposus cells and its mechanism [J]. Biomedicine & Pharmacotherapy = Biomedecine & Pharmacotherapie, 2020, 123: 109738.

[62] ZHANG H, QIN H, ZHOU C, et al. Gene expression profile of lipopolysaccharide‑induced apoptosis of nucleus pulposus cells reversed by syringic acid [J]. Molecular Medicine Reports, 2020, 22(6): 5012-22.

[63] HUA W, LI S, LUO R, et al. Icariin protects human nucleus pulposus cells from hydrogen peroxide-induced mitochondria-mediated apoptosis by activating nuclear factor erythroid 2-related factor 2 [J]. Biochim Biophys Acta Mol Basis Dis, 2020, 1866(1): 165575.

[64] WANG D, HE X, WANG D, et al. Quercetin Suppresses Apoptosis and Attenuates Intervertebral Disc Degeneration via the SIRT1-Autophagy Pathway [J]. Frontiers In Cell and Developmental Biology, 2020, 8: 613006.

[65] TANG N, DONG Y, CHEN C, et al. Anisodamine Maintains the Stability of Intervertebral Disc Tissue by Inhibiting the Senescence of Nucleus Pulposus Cells and Degradation of Extracellular Matrix via Interleukin-6/Janus Kinases/Signal Transducer and Activator of Transcription 3 Pathway [J]. Front Pharmacol, 2020, 11: 519172.

[66] OGUNLADE B, FIDELIS O P, ADELAKUN S A, et al. Grape seed extract inhibits nucleus pulposus cell apoptosis and attenuates annular puncture induced intervertebral disc degeneration in rabbit model [J]. Anat Cell Biol, 2020, 53(3): 313-24.

[67] TIAN Y, BAO Z, JI Y, et al. Epigallocatechin-3-Gallate Protects H2O2-Induced Nucleus Pulposus Cell Apoptosis and Inflammation by Inhibiting cGAS/Sting/NLRP3 Activation [J]. Drug Des Devel Ther, 2020, 14: 2113-22.

[68] WANG L, GU Y, ZHAO H, et al. Dioscin Attenuates Interleukin 1β (IL-1β)-Induced Catabolism and Apoptosis via Modulating the Toll-Like Receptor 4 (TLR4)/Nuclear Factor kappa B (NF-κB) Signaling in Human Nucleus Pulposus Cells [J]. Med Sci Monit, 2020, 26: e923386.

[69] NAN L-P, WANG F, RAN D, et al. Naringin alleviates H2O2-induced apoptosis via the PI3K/Akt pathway in rat nucleus pulposus-derived mesenchymal stem cells [J]. Connect Tissue Res, 2020, 61(6): 554-67.

[70] HUANG D, PENG Y, MA K, et al. Puerarin Relieved Compression-Induced Apoptosis and Mitochondrial Dysfunction in Human Nucleus Pulposus Mesenchymal Stem Cells via the PI3K/Akt Pathway [J]. Stem Cells Int, 2020, 2020: 7126914.

[71] HUANG J-F, ZHENG X-Q, LIN J-L, et al. Sinapic Acid Inhibits IL-1β-Induced Apoptosis and Catabolism in Nucleus Pulposus Cells and Ameliorates Intervertebral Disk Degeneration [J]. J Inflamm Res, 2020, 13: 883-95.

[72] LIU J, JIANG T, HE M, et al. Andrographolide prevents human nucleus pulposus cells against degeneration by inhibiting the NF-κB pathway [J]. Journal of Cellular Physiology, 2019, 234(6): 9631-9.

[73] LUO R, LIAO Z, SONG Y, et al. Berberine ameliorates oxidative stress-induced apoptosis by modulating ER stress and autophagy in human nucleus pulposus cells [J]. Life Sciences, 2019, 228: 85-97.

[74] WANG Y, ZUO R, WANG Z, et al. Kinsenoside ameliorates intervertebral disc degeneration through the activation of AKT-ERK1/2-Nrf2 signaling pathway [J]. Aging, 2019, 11(18): 7961-77.

[75] WANG K, CHEN T, YING X, et al. Ligustilide alleviated IL-1β induced apoptosis and extracellular matrix degradation of nucleus pulposus cells and attenuates intervertebral disc degeneration in vivo [J]. International Immunopharmacology, 2019, 69: 398-407.

[76] LIU X, ZHUANG J, WANG D, et al. Glycyrrhizin suppresses inflammation and cell apoptosis by inhibition of HMGB1 via p38/p-JUK signaling pathway in attenuating intervertebral disc degeneration [J]. Am J Transl Res, 2019, 11(8): 5105-13.

[77] WANG K, HU S, WANG B, et al. Genistein protects intervertebral discs from degeneration via Nrf2-mediated antioxidant defense system: An in vitro and in vivo study [J]. Journal of Cellular Physiology, 2019, 234(9): 16348-56.

[78] LU L, HU J, WU Q, et al. Berberine prevents human nucleus pulposus cells from IL‑1β‑induced extracellular matrix degradation and apoptosis by inhibiting the NF‑κB pathway [J]. Int J Mol Med, 2019, 43(4): 1679-86.

[79] CHEN J, LIU G-Z, SUN Q, et al. Protective effects of ginsenoside Rg3 on TNF-α-induced human nucleus pulposus cells through inhibiting NF-κB signaling pathway [J]. Life Sciences, 2019, 216: 1-9.

[80] LIN J, CHEN J, ZHANG Z, et al. Luteoloside Inhibits IL-1β-Induced Apoptosis and Catabolism in Nucleus Pulposus Cells and Ameliorates Intervertebral Disk Degeneration [J]. Front Pharmacol, 2019, 10: 868.

[81] ZHANG L, CHEN Q, WANG H, et al. Andrographolide mitigates IL‑1β‑induced human nucleus pulposus cells degeneration through the TLR4/MyD88/NF‑κB signaling pathway [J]. Molecular Medicine Reports, 2018, 18(6): 5427-36.

[82] TANG P, GU J-M, XIE Z-A, et al. Honokiol alleviates the degeneration of intervertebral disc via suppressing the activation of TXNIP-NLRP3 inflammasome signal pathway [J]. Free Radic Biol Med, 2018, 120: 368-79.

[83] GUO M-B, WANG D-C, LIU H-F, et al. Lupeol against high-glucose-induced apoptosis via enhancing the anti-oxidative stress in rabbit nucleus pulposus cells [J]. Eur Spine J, 2018, 27(10): 2609-20.

[84] LI K, LI Y, MI J, et al. Resveratrol protects against sodium nitroprusside induced nucleus pulposus cell apoptosis by scavenging ROS [J]. Int J Mol Med, 2018, 41(5): 2485-92.

[85] HUANG Y, CHEN J, JIANG T, et al. Gallic acid inhibits the release of ADAMTS4 in nucleus pulposus cells by inhibiting p65 phosphorylation and acetylation of the NF-κB signaling pathway [J]. Oncotarget, 2017, 8(29): 47665-74.

[86] DENG X, CHEN S, ZHENG D, et al. Icariin Prevents H2O2-Induced Apoptosis via the PI3K/Akt Pathway in Rat Nucleus Pulposus Intervertebral Disc Cells [J]. Evid Based Complement Alternat Med, 2017, 2017: 2694261.

[87] DENG X, WU W, LIANG H, et al. Icariin Prevents IL-1β-Induced Apoptosis in Human Nucleus Pulposus via the PI3K/AKT Pathway [J]. Evid Based Complement Alternat Med, 2017, 2017: 2198323.

[88] PAN Y, CHEN D, LU Q, et al. Baicalin prevents the apoptosis of endplate chondrocytes by inhibiting the oxidative stress induced by H2O2 [J]. Molecular Medicine Reports, 2017, 16(3): 2985-91.

[89] CHU H, YU H, REN D, et al. Plumbagin exerts protective effects in nucleus pulposus cells by attenuating hydrogen peroxide-induced oxidative stress, inflammation and apoptosis through NF-κB and Nrf-2 [J]. Int J Mol Med, 2016, 37(6): 1669-76.

[90] CHEN J, HOU C, CHEN X, et al. Protective effect of cannabidiol on hydrogen peroxide‑induced apoptosis, inflammation and oxidative stress in nucleus pulposus cells [J]. Molecular Medicine Reports, 2016, 14(3): 2321-7.

[91] SHI L, TENG H, ZHU M, et al. Paeoniflorin inhibits nucleus pulposus cell apoptosis by regulating the expression of Bcl-2 family proteins and caspase-9 in a rabbit model of intervertebral disc degeneration [J]. Experimental and Therapeutic Medicine, 2015, 10(1): 257-62.

[92] CHEN S-Q, LIN J-P, ZHENG Q-K, et al. Protective effects of paeoniflorin against FasL-induced apoptosis of intervertebral disc annulus fibrosus cells via Fas-FasL signalling pathway [J]. Experimental and Therapeutic Medicine, 2015, 10(6): 2351-5.

[93] NIU K, ZHAO Y-J, ZHANG L, et al. [The synergistic effect of amygdalin and HSYA on the IL-1beta induced endplate chondrocytes of rat intervertebral discs] [J]. Yao Xue Xue Bao, 2014, 49(8): 1136-42.

[94] ZHANG Z, WU J, TENG C, et al. Orientin downregulating oxidative stress-mediated endoplasmic reticulum stress and mitochondrial dysfunction through AMPK/SIRT1 pathway in rat nucleus pulposus cells in vitro and attenuated intervertebral disc degeneration in vivo [J]. Apoptosis : an International Journal On Programmed Cell Death, 2022, 27(11-12): 1031-48.

[95] LU Y, ZHOU L, HE S, et al. Lycopene alleviates disc degeneration under oxidative stress through the Nrf2 signaling pathway [J]. Mol Cell Probes, 2020, 51: 101559.

[96] SHAO T, GAO Q, MA Y, et al. Hyperforin improves matrix stiffness induced nucleus pulposus inflammatory degeneration by activating mitochondrial fission [J]. International Immunopharmacology, 2024, 137: 112444.

[97] MAO T, FAN J. Myricetin Protects Against Rat Intervertebral Disc Degeneration Partly Through the Nrf2/HO-1/NF-κB Signaling Pathway [J]. Biochem Genet, 2024, 62(2): 950-67.

[98] XIE T, GU X, PAN R, et al. Evodiamine ameliorates intervertebral disc degeneration through the Nrf2 and MAPK pathways [J]. Cytotechnology, 2024, 76(2): 153-66.

[99] XU T, ZHAO H, FANG X, et al. Mulberroside A mitigates intervertebral disc degeneration by inhibiting MAPK and modulating Ppar-γ/NF-κB pathways [J]. J Inflamm (Lond), 2024, 21(1): 32.

[100] ZHU Z, YU Q, LI H, et al. Vanillin-based functionalization strategy to construct multifunctional microspheres for treating inflammation and regenerating intervertebral disc [J]. Bioact Mater, 2023, 28: 167-82.

[101] LU B, CHEN X, CHEN H, et al. Demethoxycurcumin mitigates inflammatory responses in lumbar disc herniation via MAPK and NF-κB pathways in vivo and in vitro [J]. International Immunopharmacology, 2022, 108: 108914.

[102] ZHENG S, MA J, ZHAO X, et al. Ganoderic Acid A Attenuates IL-1β-Induced Inflammation in Human Nucleus Pulposus Cells Through Inhibiting the NF-κB Pathway [J]. Inflammation, 2022, 45(2): 851-62.

[103] YANG X, LI B, TIAN H, et al. Curcumenol Mitigates the Inflammation and Ameliorates the Catabolism Status of the Intervertebral Discs In Vivo and In Vitro via Inhibiting the TNFα/NFκB Pathway [J]. Front Pharmacol, 2022, 13: 905966.

[104] ZHAO K, CHEN M, LIU T, et al. Rhizoma drynariae total flavonoids inhibit the inflammatory response and matrix degeneration via MAPK pathway in a rat degenerative cervical intervertebral disc model [J]. Biomedicine & Pharmacotherapy = Biomedecine & Pharmacotherapie, 2021, 138: 111466.

[105] HE S, FU Y, YAN B, et al. Curcumol Alleviates the Inflammation of Nucleus Pulposus Cells via the PI3K/Akt/NF-κB Signaling Pathway and Delays Intervertebral Disk Degeneration [J]. World Neurosurg, 2021, 155: e402-e11.

[106] DAI S, SHI X, QIN R, et al. Sodium Tanshinone IIA Sulfonate Ameliorates Injury-Induced Oxidative Stress and Intervertebral Disc Degeneration in Rats by Inhibiting p38 MAPK Signaling Pathway [J]. Oxidative Medicine and Cellular Longevity, 2021, 2021: 5556122.

[107] XIE C, MA H, SHI Y, et al. Cardamonin protects nucleus pulposus cells against IL-1β-induced inflammation and catabolism via Nrf2/NF-κB axis [J]. Food Funct, 2021, 12(6): 2703-14.

[108] WEI K, DAI J, WANG Z, et al. Oxymatrine suppresses IL-1β-induced degradation of the nucleus pulposus cell and extracellular matrix through the TLR4/NF-κB signaling pathway [J]. Exp Biol Med (Maywood), 2020, 245(6): 532-41.

[109] WANG H, JIANG Z, PANG Z, et al. Acacetin Alleviates Inflammation and Matrix Degradation in Nucleus Pulposus Cells and Ameliorates Intervertebral Disc Degeneration in vivo [J]. Drug Des Devel Ther, 2020, 14: 4801-13.

[110] SHANG P, TANG Q, HU Z, et al. Procyanidin B3 alleviates intervertebral disc degeneration via interaction with the TLR4/MD-2 complex [J]. J Cell Mol Med, 2020, 24(6): 3701-11.

[111] SHAO Z, LU J, ZHANG C, et al. Stachydrine ameliorates the progression of intervertebral disc degeneration via the PI3K/Akt/NF-κB signaling pathway: in vitro and in vivo studies [J]. Food Funct, 2020, 11(12): 10864-75.

[112] BAI X, DING W, YANG S, et al. Higenamine inhibits IL-1β-induced inflammation in human nucleus pulposus cells [J]. Biosci Rep, 2019, 39(6).

[113] JIN H, WANG Q, WU J, et al. Baicalein Inhibits the IL-1β-Induced Inflammatory Response in Nucleus Pulposus Cells and Attenuates Disc Degeneration In vivo [J]. Inflammation, 2019, 42(3): 1032-44.

[114] YANG S, LI L, ZHU L, et al. Aucubin inhibits IL-1β- or TNF-α-induced extracellular matrix degradation in nucleus pulposus cell through blocking the miR-140-5p/CREB1 axis [J]. Journal of Cellular Physiology, 2019, 234(8): 13639-48.

[115] GAO G, CHANG F, ZHANG T, et al. Naringin Protects Against Interleukin 1β (IL-1β)-Induced Human Nucleus Pulposus Cells Degeneration via Downregulation Nuclear Factor kappa B (NF-κB) Pathway and p53 Expression [J]. Med Sci Monit, 2019, 25: 9963-72.

[116] GU R, HUANG Z, LIU H, et al. Moracin attenuates LPS-induced inflammation in nucleus pulposus cells via Nrf2/HO-1 and NF-κB/TGF-β pathway [J]. Biosci Rep, 2019, 39(12).

[117] LUO L, GAO Y, YANG C, et al. Halofuginone attenuates intervertebral discs degeneration by suppressing collagen I production and inactivating TGFβ and NF-кB pathway [J]. Biomedicine & Pharmacotherapy = Biomedecine & Pharmacotherapie, 2018, 101: 745-53.

[118] FANG W, ZHOU X, WANG J, et al. Wogonin mitigates intervertebral disc degeneration through the Nrf2/ARE and MAPK signaling pathways [J]. International Immunopharmacology, 2018, 65: 539-49.

[119] HUA W, ZHANG Y, WU X, et al. Icariin Attenuates Interleukin-1β-Induced Inflammatory Response in Human Nucleus Pulposus Cells [J]. Curr Pharm Des, 2018, 23(39): 6071-8.

[120] DONG Y, LIU L, SHAN X, et al. Pilose antler peptide attenuates LPS-induced inflammatory reaction [J]. Int J Biol Macromol, 2018, 108: 272-6.

[121] CHEN J, XUAN J, GU Y-T, et al. Celastrol reduces IL-1β induced matrix catabolism, oxidative stress and inflammation in human nucleus pulposus cells and attenuates rat intervertebral disc degeneration in vivo [J]. Biomedicine & Pharmacotherapy = Biomedecine & Pharmacotherapie, 2017, 91: 208-19.

[122] CHEN B, WANG H-T, YU B, et al. Carthamin yellow inhibits matrix degradation and inflammation induced by LPS in the intervertebral disc via suppression of MAPK pathway activation [J]. Experimental and Therapeutic Medicine, 2017, 14(2): 1614-20.

[123] LI K, LI Y, XU B, et al. Sesamin inhibits lipopolysaccharide-induced inflammation and extracellular matrix catabolism in rat intervertebral disc [J]. Connect Tissue Res, 2016, 57(5): 347-59.

[124] LI Y, LI K, MAO L, et al. Cordycepin inhibits LPS-induced inflammatory and matrix degradation in the intervertebral disc [J]. PeerJ, 2016, 4: e1992.

[125] MA T, GUO C J, ZHAO X, et al. The effect of curcumin on NF-κB expression in rat with lumbar intervertebral disc degeneration [J]. European review for medical and pharmacological sciences, 2015, 19(7): 1305-14.

[126] LI Y, LI K, HU Y, et al. Piperine mediates LPS induced inflammatory and catabolic effects in rat intervertebral disc [J]. Int J Clin Exp Pathol, 2015, 8(6): 6203-13.

[127] LI W, ZHANG Y, XING C, et al. Tanshinone IIA represses inflammatory response and reduces radiculopathic pain by inhibiting IRAK-1 and NF-κB/p38/JNK signaling [J]. International Immunopharmacology, 2015, 28(1): 382-9.

[128] LI K, LI Y, MA Z, et al. Crocin exerts anti-inflammatory and anti-catabolic effects on rat intervertebral discs by suppressing the activation of JNK [J]. Int J Mol Med, 2015, 36(5): 1291-9.

[129] KRUPKOVA O, SEKIGUCHI M, KLASEN J, et al. Epigallocatechin 3-gallate suppresses interleukin-1β-induced inflammatory responses in intervertebral disc cells in vitro and reduces radiculopathic pain in rats [J]. Eur Cell Mater, 2014, 28: 372-86.

[130] WANG G, HUANG K, DONG Y, et al. Lycorine Suppresses Endplate-Chondrocyte Degeneration and Prevents Intervertebral Disc Degeneration by Inhibiting NF-κB Signalling Pathway [J]. Cell Physiol Biochem, 2018, 45(3): 1252-69.

[131] LIANG Q-Q, ZHANG M, ZHOU Q, et al. Muscone protects vertebral end-plate degeneration by antiinflammatory property [J]. Clin Orthop Relat Res, 2010, 468(6): 1600-10.

[132] GAO W, BAO J, ZHANG Y, et al. Injectable kaempferol-loaded fibrin glue regulates the metabolic balance and inhibits inflammation in intervertebral disc degeneration [J]. Scientific Reports, 2023, 13(1): 20001.

[133] QUE Y, WONG C, QIU J, et al. Maslinic acid alleviates intervertebral disc degeneration by inhibiting the PI3K/AKT and NF-κB signaling pathways [J]. Acta Biochimica Et Biophysica Sinica, 2024, 56(5): 776-88.

[134] ZHAO W-J, LIU X, HU M, et al. Quercetin ameliorates oxidative stress-induced senescence in rat nucleus pulposus-derived mesenchymal stem cells via the miR-34a-5p/SIRT1 axis [J]. World J Stem Cells, 2023, 15(8): 842-65.

[135] CHEN R, ZHANG X, ZHU X, et al. Myricetin alleviated hydrogen peroxide-induced cellular senescence of nucleus pulposus cell through regulating SERPINE1 [J]. Journal of Orthopaedic Surgery and Research, 2023, 18(1): 143.

[136] LIAO Z, SU D, LIU H, et al. Dihydroartemisinin Attenuated Intervertebral Disc Degeneration via Inhibiting PI3K/AKT and NF-κB Signaling Pathways [J]. Oxidative Medicine and Cellular Longevity, 2022, 2022: 8672969.

[137] SHENG K, LI Y, WANG Z, et al. p-Coumaric acid suppresses reactive oxygen species-induced senescence in nucleus pulposus cells [J]. Experimental and Therapeutic Medicine, 2022, 23(2): 183.

[138] SHAO Z, WANG B, SHI Y, et al. Senolytic agent Quercetin ameliorates intervertebral disc degeneration via the Nrf2/NF-κB axis [J]. Osteoarthritis Cartilage, 2021, 29(3): 413-22.

[139] MANNARINO M, CHERIF H, LI L, et al. Toll-like receptor 2 induced senescence in intervertebral disc cells of patients with back pain can be attenuated by o-vanillin [J]. Arthritis Res Ther, 2021, 23(1): 117.

[140] CHEN Z, YANG X, ZHOU Y, et al. Dehydrocostus Lactone Attenuates the Senescence of Nucleus Pulposus Cells and Ameliorates Intervertebral Disc Degeneration via Inhibition of STING-TBK1/NF-κB and MAPK Signaling [J]. Front Pharmacol, 2021, 12: 641098.

[141] YANG H, YANG X, RONG K, et al. Eupatilin attenuates the senescence of nucleus pulposus cells and mitigates intervertebral disc degeneration via inhibition of the MAPK/NF-κB signaling pathway [J]. Front Pharmacol, 2022, 13: 940475.

[142] BARI E, PERTEGHELLA S, RASSU G, et al. Sericin/crocetin micro/nanoparticles for nucleus pulposus cells regeneration: An "active" drug delivery system [J]. Front Pharmacol, 2023, 14: 1129882.

[143] YI Y-Y, ZHANG S-B, CHEN H, et al. Ascorbic acid promotes nucleus pulposus cell regeneration by regulating proliferation during intervertebral disc degeneration [J]. J Nutr Biochem, 2022, 108: 109099.

[144] XU Y, HE J, HE J. Cyanidin attenuates the high hydrostatic pressure-induced degradation of cellular matrix of nucleus pulposus cell via blocking the Wnt/β-catenin signaling [J]. Tissue Cell, 2022, 76: 101798.

[145] LIU H, SHEN J, ZHOU H, et al. [Resveratrol regulate the extracellular matrix expression via Wnt/β-catenin pathway in nucleus pulposus cells] [J]. Zhongguo Xiu Fu Chong Jian Wai Ke Za Zhi, 2018, 32(4): 476-83.

[146] LI N, WHITAKER C, XU Z, et al. Therapeutic effects of naringin on degenerative human nucleus pulposus cells for discogenic low back pain [J]. Spine J, 2016, 16(10): 1231-7.

[147] KRUPKOVA O, HANDA J, HLAVNA M, et al. The Natural Polyphenol Epigallocatechin Gallate Protects Intervertebral Disc Cells from Oxidative Stress [J]. Oxidative Medicine and Cellular Longevity, 2016, 2016: 7031397.

[148] SHEN J-L, HU Z-M, ZHONG X-M, et al. [Resveratrol stimulates extracellular matrix synthesis in degenerative nucleus pulposus cells via upregulation of SIRT1] [J]. Xi Bao Yu Fen Zi Mian Yi Xue Za Zhi, 2012, 28(11): 1146-50.

[149] ZHAN J-W, WANG S-Q, CHEN M, et al. [Salvianolic acid A contributes to cartilage endplate cell restoration by regulating miR-940 and miR-576-5p] [J]. Zhongguo Gu Shang, 2023, 36(10): 982-9.

[150] ZHANG H, JIANG W, JIANG Y, et al. Investigating the therapeutic potential of hesperidin targeting CRISP2 in intervertebral disc degeneration and cancer risk mitigation [J]. Front Pharmacol, 2024, 15: 1447152.

[151] LAMBERTINI E, PENOLAZZI L, NOTARANGELO M P, et al. Pro‑differentiating compounds for human intervertebral disc cells are present in Violina pumpkin leaf extracts [J]. Int J Mol Med, 2023, 51(5).

[152] DEVRAJ V M, VEMURI S K, BANALA R R, et al. Evaluation of Anti-inflammatory and Regenerative Efficiency of Naringin and Naringenin in Degenerated Human Nucleus Pulposus Cells: Biological and Molecular Modeling Studies [J]. Asian Spine J, 2019, 13(6): 875-89.

[153] LIU S, CHENG Y, TAN Y, et al. Ligustrazine Prevents Intervertebral Disc Degeneration via Suppression of Aberrant TGFβ Activation in Nucleus Pulposus Cells [J]. BioMed Research International, 2019, 2019: 5601734.

[154] HU Y, TANG J-S, HOU S-X, et al. Neuroprotective effects of curcumin alleviate lumbar intervertebral disc degeneration through regulating the expression of iNOS, COX‑2, TGF‑β1/2, MMP‑9 and BDNF in a rat model [J]. Molecular Medicine Reports, 2017, 16(5): 6864-9.
