# Supplementary material for: Natural products targeting programmed cell death: a novel therapeutic strategy for intervertebral disc degeneration
Source: Int J Surg. 2025 Oct 7;112(1):1601–34. doi: 10.1097/JS9.0000000000003380 (PMC12825861; doi:10.1097/JS9.0000000000003380)
Supplement: Supplementary file 2 [file js9-112-1601-002.docx]

**Abbreviations：**IVDD, Intervertebral disc degeneration; IVD, Intervertebral disc; PCD, Programmed cell death; NPCs, Nucleus pulposus cells; NP, Nucleus pulposus; AFCs, Annulus fibrosus cells; CEPCs, Cartilage endplate cells; Caspase, Cysteine-aspartic proteases; ERS, Endoplasmic reticulum stress; MOMP, Mitochondrial outer membrane permeabilization; Cyt C, Cytochrome c; Apaf-1, Apoptotic protease activating factor-1; CARD, Caspase recruitment domain; Bcl-2, B-cell lymphoma-2; BAX, Bcl-2 associated X protein; BAK, Bcl-2 associated K protein; Bcl-xL, B-cell lymphoma-extra large; BH3, Bcl-2 homology region 3; Smac, Second mitochondria-derived activator of caspases; XIAP, X-linked inhibitor of apoptosis protein; CICD, Caspase-independent cell death; AIF, Apoptosis-inducing factor; ER, Endoplasmic reticulum; ; UPR, unfolded protein response; IRE1α, inositol-requiring enzyme 1α, PERK, protein kinase RNA-like ER kinase; ATF6, activating transcription factor 6; GRP78/BiP, Glucose regulated protein 78Kd; XBP1, X-box binding protein 1; TRAF2, Tumor necrosis factor receptor-associated factor 2; TRAF5, Tumor necrosis factor receptor-associated factor 5; ASK1, Apoptosis signal-regulating kinase 1; JNK, c-Jun N-terminal kinase; eIF2α, eukaryotic initiation factor 2α; ATF4, Activating transcription factor 4; CHOP, C/EBP homologous protein; DR5 Death receptor 5; MCL-1,myeloid cell leukemia-1; TNFR, Tumor necrosis factor receptor; DD, Death domain; FasL, Fatty acid synthetase ligand; FasR, Fatty acid synthetase receptor; TNF-α, tumor necrosis factor-alpha; TNFR1, Tumor necrosis factor receptor 1; TRAIL, TNF-related apoptosis-inducing ligand; DR4,Death receptor4; DED, Death effector domain; DISC, death inducing signaling complex; TRADD, TNFR1 associated death domain protein; RIPK1, Receptor interacting protein kinase 1; RIPK3, Receptor interacting protein kinase 3; cIAP1, Cellular inhibitor of apoptosis proteins 1; cIAP2, Cellular inhibitor of apoptosis proteins 2; ECM, extracellular matrix; AGEs, Advanced glycation end products; IL-1β, Interleukin - 1β; IL-1α, Interleukin - 1α; ROS, Reactive oxygen species; IL-2, Interleukin-2; AOPPs, Advanced oxidation protein products; MAPK, Mitogen-activated protein kinase; TGF-β1, Transforming growth factor beta 1; ERK, Extracellular signal-regulated kinase; PI3K, Phosphatidylinositol 3-Kinase; ASC, Apoptosis-associated speck-like protein containing a CARD; GSDM, Gasdermin; GSDMD, Gasdermin D; GSDME, Gasdermin E; PAMPs, Pathogen-associated molecular patterns; DAMPs, Damage-associated molecular patterns; ATP, Adenosine triphosphate; PRRs, Pattern recognition receptors; NLRs,NOD-like receptor proteins; LRRs, Leucine-rich repeats; PYD, pyrin domain; NLRP1, NLR Family Pyrin Domain Containing 1; NLRP3, NLR Family Pyrin Domain Containing 3; NLRC4, NLR Family CARD Domain Containing 4; LPS, Lipopolysaccharide; IL-18, Interleukin - 18; GzmB, granzyme B; PINK1, PTEN induced putative kinase 1; MFG-E8, Milk fat globule-epidermal growth factor 8; Nrf2, Nuclear factor erythroid 2-related factor 2; TXNIP, Thioredoxin Interacting Protein; cGAS, cyclic GMP-AMP synthase; STING, Stimulator of interferon genes; THP1, Tohoku Hospital Pediatrics-1; TLR4, Toll-like receptor 4; TLR3, Toll-like receptor 3; IP6, Inositol hexaphosphate; PIP, Phosphatidylinositol phosphate; HSP90, heat shock protein 90; Drp-1, Dynamin-related protein 1; MyD88, myeloid differentiation primary response protein 88; iNOS, inducible Nitric Oxide Synthase; COX-2, Cyclooxygenase-2; TF, Transferrin; TFRC, Transferrin receptor; PKC, Protein kinase C; HSPB1, Heat shock protein B1; STEAP3,Six-transmembrane epithelial antigen of the prostate 3; SLC11A2, Solute carrier family 11 member 2; CISD1, CDGSH iron-sulfur domain-containing protein 1; CISD2, CDGSH iron-sulfur domain-containing protein 2; PUFAs, Polyunsaturated fatty acids; LA, Linoleic acid; AA, Arachidonic acid; AdA, Adrenic acid; PLA2, Phospholipase A2; ACSL3, Acyl-coenzyme A synthetase long-chain family member 3; ACSL4, Acyl-coenzyme A synthetase long-chain family member 4; LPCAT3, Lysophosphatidylcholine acyltransferase 3; MUFAs, Monounsaturated fatty acids; SCD-1, Stearoyl- coenzyme A desaturase-1; POR, Cytochrome P450 oxidoreductase; FMN, Flavin mononucleotide; FAD, Flavin adenine dinucleotide; NADPH, Nicotinamide adenine dinucleotide phosphate; GPX4, Glutathione peroxidase 4; SLC7A11,Solute carrier family 7 member 11; FTH1, Ferritin heavy chain 1; SelK, selenoprotein K; USP11, Ubiquitin specific peptidase 11; SIRT3, Sirtuin 3; MGST1,Microsomal glutathione s-transferase 1; EGR1, Early growth response 1; HIF-2α, Hypoxia-inducible factor-2α;TFR1, Transferrin receptor 1; NPMSCs, Nucleus pulposus mesenchymal stem cells; Atg1, Autophagy-related gene 1; ULK1, Unc-51-like kinase 1; ULK2, Unc-51-like kinase 2; ATG11, Autophagy-related gene 11; ATG13, Autophagy-related gene 13; ATG101, Autophagy-related gene 101; mTORC1, mechanistic target of rapamycin complex 1; AMPK,AMP-activated protein kinase; ATG14, Autophagy-related gene 14; VPS34, Vacuolar protein sorting 34; VPS15, Vacuolar protein sorting 15; PI3P, Phosphatidylinositol 3-phosphate; ATG9, Autophagy-related gene 9;WIPI, WD-repeat protein Interacting with PhosphoInositides; ATG2A,Autophagy-related protein 2A; ATG2B,Autophagy-related protein 2B; ATG12,Autophagy-related protein 12; ATG5,Autophagy-related protein 5; ATG16L1, Autophagy-related protein 16-like 1; ATG8, Autophagy-related protein 8; PE, Phosphatidylethanolamine; LC3, Microtubule - associated protein 1 light chain 3; NDP52, Nuclear dot protein 52; ADCD, Autophagy-dependent cell death; AMCD, Autophagy-mediated cell death; RCD, Regulated cell death; LAMP-2A, Lysosome-associated membrane glycoprotein 2; mTOR, mechanistic target of rapamycin; NCOA4, nuclear receptor co-activator 4.
